# Assembling a plug-and-play production line for combinatorial biosynthesis of aromatic polyketides in *Escherichia coli*

**DOI:** 10.1371/journal.pbio.3000347

**Published:** 2019-07-18

**Authors:** Matthew Cummings, Anna D. Peters, George F. S. Whitehead, Binuraj R. K. Menon, Jason Micklefield, Simon J. Webb, Eriko Takano

**Affiliations:** 1 Manchester Synthetic Biology Research Centre SYNBIOCHEM, Manchester Institute of Biotechnology, School of Chemistry, The University of Manchester, Manchester, United Kingdom; 2 Warwick Integrative Synthetic Biology Centre, WISB, School of Life Sciences, The University of Warwick, Coventry, United Kingdom; Stanford University, UNITED STATES

## Abstract

Polyketides are a class of specialised metabolites synthesised by both eukaryotes and prokaryotes. These chemically and structurally diverse molecules are heavily used in the clinic and include frontline antimicrobial and anticancer drugs such as erythromycin and doxorubicin. To replenish the clinicians’ diminishing arsenal of bioactive molecules, a promising strategy aims at transferring polyketide biosynthetic pathways from their native producers into the biotechnologically desirable host *Escherichia coli*. This approach has been successful for type I modular polyketide synthases (PKSs); however, despite more than 3 decades of research, the large and important group of type II PKSs has until now been elusive in *E*. *coli*. Here, we report on a versatile polyketide biosynthesis pipeline, based on identification of *E*. *coli*–compatible type II PKSs. We successfully express 5 ketosynthase (KS) and chain length factor (CLF) pairs—e.g., from *Photorhabdus luminescens* TT01, *Streptomyces resistomycificus*, *Streptoccocus* sp. GMD2S, *Pseudoalteromonas luteoviolacea*, and *Ktedonobacter racemifer*—as soluble heterodimeric recombinant proteins in *E*. *coli* for the first time. We define the anthraquinone minimal PKS components and utilise this biosynthetic system to synthesise anthraquinones, dianthrones, and benzoisochromanequinones (BIQs). Furthermore, we demonstrate the tolerance and promiscuity of the anthraquinone heterologous biosynthetic pathway in *E*. *coli* to act as genetically applicable plug-and-play scaffold, showing it to function successfully when combined with enzymes from phylogenetically distant species, endophytic fungi and plants, which resulted in 2 new-to-nature compounds, neomedicamycin and neochaetomycin. This work enables plug-and-play combinatorial biosynthesis of aromatic polyketides using bacterial type II PKSs in *E*. *coli*, providing full access to its many advantages in terms of easy and fast genetic manipulation, accessibility for high-throughput robotics, and convenient biotechnological scale-up. Using the synthetic and systems biology toolbox, this plug-and-play biosynthetic platform can serve as an engine for the production of new and diversified bioactive polyketides in an automated, rapid, and versatile fashion.

## Abstract 2

This study describes a “plug-and-play” synthetic biology platform for producing variants of type II polyketides in Escherichia coli, opening the door for high-throughput discovery of antimicrobials and other active pharmaceutical ingredients.

## Introduction

Natural products and their synthetic derivatives provide important clinically used therapeutic agents, accounting for 73% of antibacterial agents and 83% of anticancer agents approved by the Food and Drug Administration between 1981 and 2014 [1]. Polyketides represent a central class of these natural products, with remarkably targeted and potent pharmacological properties and highly diverse chemical structures. Although their native biological role is still debated, polyketides continue to have significant medical value as potent antitumor agents, antibiotics, immunosuppressants, antiparasitics, and cholesterol-lowering agents, among other applications [2]. The chemistry underpinning polyketide biosynthesis is widely conserved and carried out by biosynthetic machinery of 3 major classes [3]. In almost all cases, the polyketide biosynthesis machinery is highly modular at the genetic, enzymatic, and chemical level [4]. This intrinsic modularity of polyketide synthases (PKSs) was a key motivation behind classical approaches to derivatisation of natural products, and for the same reason PKSs have been favorite targets for the recent pathway engineering and natural product derivatisation renaissance using synthetic biology [5].

**Fig 1:**
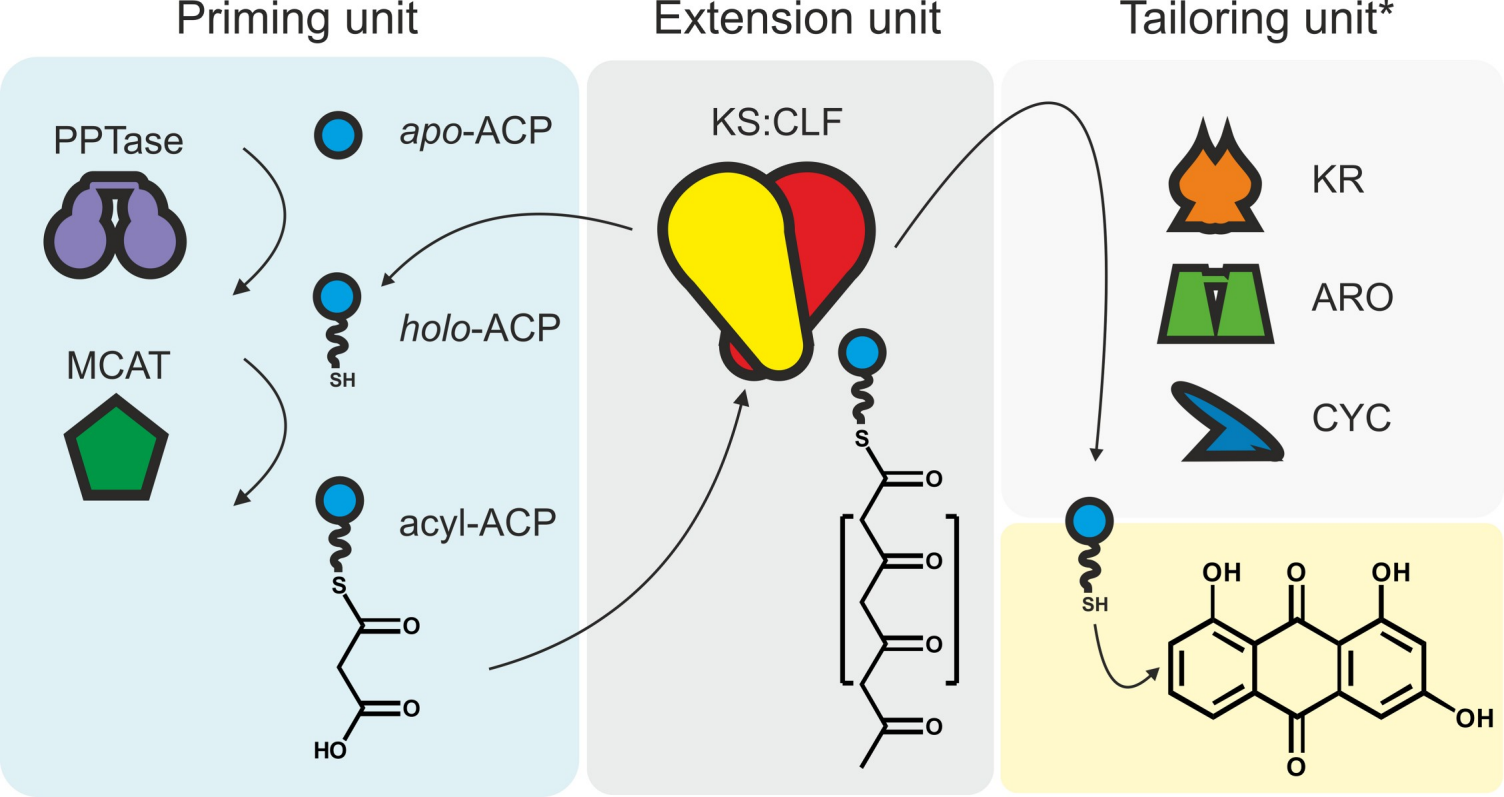
Biosynthesis of archetypal aromatic polyketides. The central dark grey pane (extension units) illustrates biosynthesis of the nascent poly-β-ketide chain by the KS/CLF heterodimer that remains tethered to the ACP. The red moiety represents the starter unit, which can be altered for increased product diversity. The left blue pane shows the enzymatic constituents necessary to prime type II polyketide biosynthesis (priming units), e.g., phosphopantetheinylation of ACP from *apo*-ACP to *holo*-ACP catalyzed by a 4′ PPTase, and acylation of the *holo*-ACP with the starter unit, e.g., via an MCAT, acyl-ACP synthetase, or via self-malonylation. The top right light grey pane (tailoring units) illustrates the typical primary tailoring enzymes. The bottom right yellow pane indicates the point at which the modified polyketide chain is released from the ACP. The free metabolite can be further modified by secondary tailoring enzymes that do not interact with the polyketide-ACP intermediate, e.g., glycosyltransferases, prenyltransferases, or halogenases, to introduce chemical diversity. *Only primary tailoring enzymes are shown schematically. ACP, acyl carrier protein; ARO, aromatase; CLF, chain length factor; CYC, cyclases that are responsible for biosynthesis of the aromatic carbon core scaffold; KR, ketoreductase; KS, ketosynthase; MCAT, malonyl-CoA:ACP transacylase; PPTase, phosphopantetheinyl transferase.

Polyketide biosynthesis is exceptionally diverse within the phylum Actinobacteria, and members of this taxon have been the source of numerous successful therapeutics. The Actinobacteria undergo complex morphological differentiation during different phases of growth, typically grow slowly, are fastidious, are not able to be grown in 96-well microtitre plates, and often are not genetically tractable. Though some Actinobacteria (e.g., *Streptomyces albus*) have more convenient features, these attributes make Actinobacteria unattractive hosts for the synthetic biologist to engineer polyketide biosynthetic gene clusters in large numbers, and alternative heterologous hosts are preferentially utilised, including *E*. *coli*. This is especially pertinent when intending to exploit the advantages of high-throughput pathway assembly using robotics. However, despite major efforts over several decades, heterologous overexpression of aromatic producing bacterial type II PKS machinery—specifically, the minimal polyketide synthase (mPKS) comprising a ketosynthase (KS) and chain length factor (CLF) and acyl carrier protein (ACP) (Fig 1)—in the biotechnologically favourable host species *E*. *coli* has remained elusive. A large number of combinatorial biosynthetic experiments have demonstrated the promiscuity and potential of type II PKS enzymatic components to synthesise new chemical entities; however, almost all have necessitated the use of an actinobacterial expression host.

Considerable effort has been made to circumvent this problematic bottleneck. An engineered nonreducing fungal PKS (NR-PKS) has been expressed in *E*. *coli* together with type II PKS cyclases to produce the nonaketide shunt metabolite SEK26 by Zhang and colleagues (Proceedings of the National Academy of Sciences, 2008) [10]. However, expanding the chemical space is difficult when using poorly understood fungal NR-PKS; e.g., to alter the polyketide chain length, an entirely new NR-PKS would be required [6,7], whilst chain length can be altered easily when using the bacterial type II machinery through introducing as little as 1 amino acid substitution [6]. Furthermore, without a starter unit loading domain, fungal NR-PKSs cannot introduce important non-acetyl starter units into aromatic polyketides, whilst these can be introduced to dissociable bacterial type II PKSs pathways [7, 8] through priming unit substitutions (Fig 1), without a need to engineer the PKS. Interestingly, the aromatic polyketide oxytetracycline (OTC) has been detected in *E*. *coli* overexpressing both the complete native OTC biosynthesis gene cluster (BGC) from *Streptomyces rimosus* and the *E*. *coli* alternative sigma factor σ54; however, the key enzymes, OxyA (KS) and OxyB (CLF), were not detectable among the soluble or insoluble proteins [9]. Other attempts to achieve mPKS expression in *E*. *coli* have either resulted in unobservable expression or inactive inclusion body formation [9–11]. The cause of KS/CLF insolubility has not been experimentally characterised, but inharmonious rates of translation, protein folding, and heterodimerisation have all been suggested as contributing factors [11].

The intractability of this class of bacterial PKSs in *E*. *coli* is limiting next-generation combinatorial approaches for the discovery of new and potent aromatic polyketide therapeutics. Actinobacteria, main natural hosts of type II PKSs, are not currently suitable for automated high-throughput technologies, due to their slow and often unpredictable filamentous growth behavior and their requirement for high aeration. Whilst many important combinatorial studies have taken place in Actinobacteria, translation of type II PKSs into biotechnologically suitable hosts will undoubtedly expedite automated generation of chemically diverse aromatic polyketide libraries.

**Fig 2:**
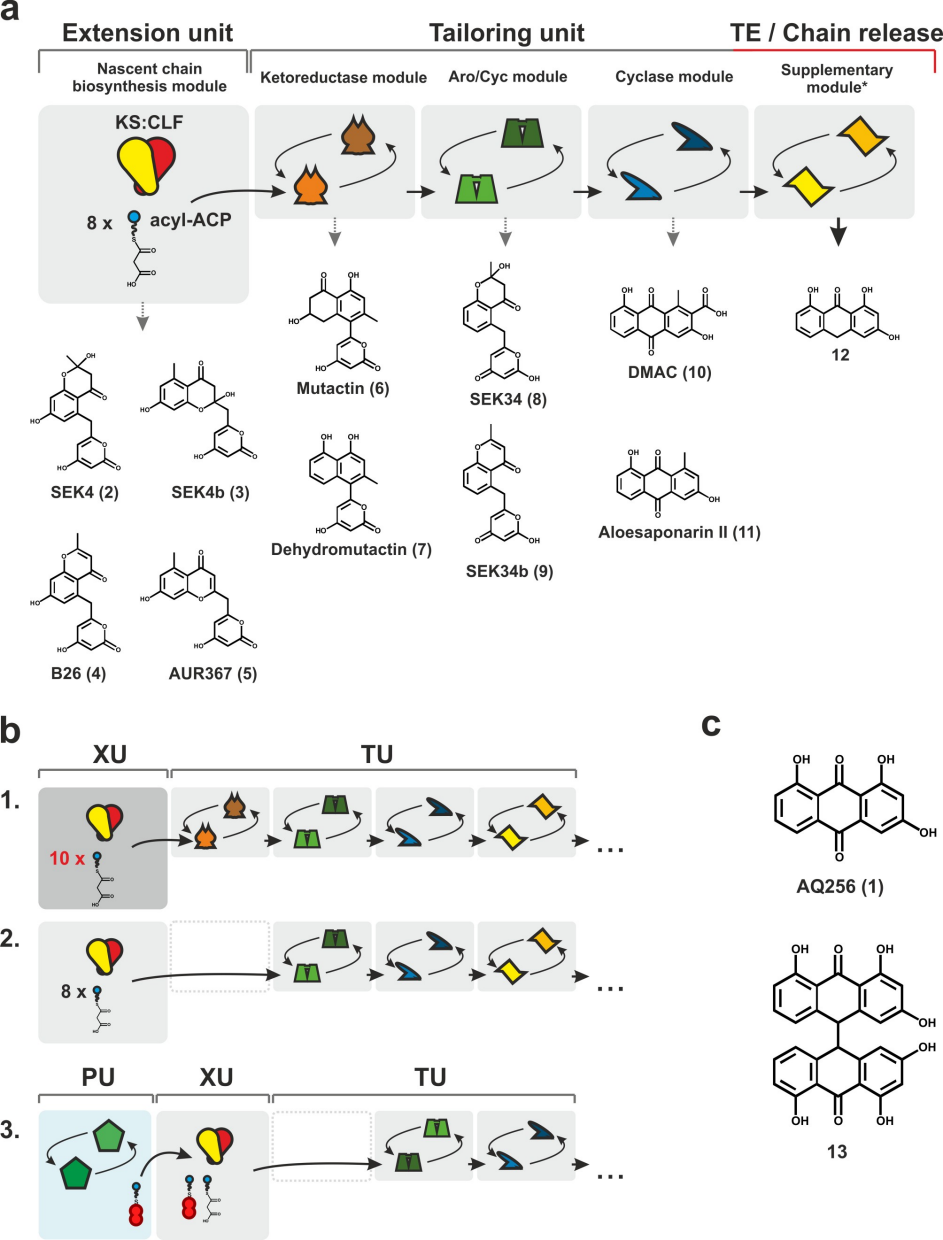
Schematic representation of aromatic polyketide biosynthesis. (a) Expected octaketide shunt metabolites after each biosynthetic step are designated by grey dotted line. Biosynthetic steps are confined to individual grey boxes that proceed in the order of biosynthesis. Circular arrows within each box represent the ability to functionally substitute biosynthetic enzymes for homologues. (b) Examples of plausible biosynthetic pathway perturbations: b1, the exchange of an octaketide producing PKS heterodimer with a decaketide producer (XU exchange); b2, compound maturation despite loss of KR (TU modification); and b3, alteration of polyketide starter unit by exchanging PU enzyme constituents as well as functional exchange of an aromatase/cyclase and loss of supplementary enzymes (PU and TU exchange). Supplementary enzymes can be variable in function. (c) Structures of AQ256 (1) and its dianthrone (13). ACP, acyl carrier protein; Aro/Cyc, aromatase/cyclase; CLF, chain length factor; DMAC, 3,8-dihydroxy-methylanthraquinone carboxylic acid; KS, ketosynthase; PKS, polyketide synthase; PU, priming unit; TU, tailoring unit; XU, extension unit.

Here, we report the successful functional expression of a soluble and heterodimeric bacterial type II PKS in *E*. *coli* for the first time, establishing a plug-and-play production line that opens the door to successful biochemical diversification and biotechnological exploitation of polyketides in a versatile and tractable heterologous expression host. We exemplify the value of this platform as a plug-and-play scaffold by demonstrating tolerance and promiscuity of the recombinant biosynthetic pathway to function successfully when complemented with sequence diverse and structurally diverse homologues from phylogenetically distant species. This *E*. *coli*–based platform can now serve as a starting point for iterative *E*. *coli*–based exploration of aromatic polyketide biosynthesis in a combinatorial fashion using a highly modular approach (Fig 2).

## Results

### Identification of candidate KS/CLF dimer pairs for heterologous expression in *E*. *coli*

**Fig 3:**
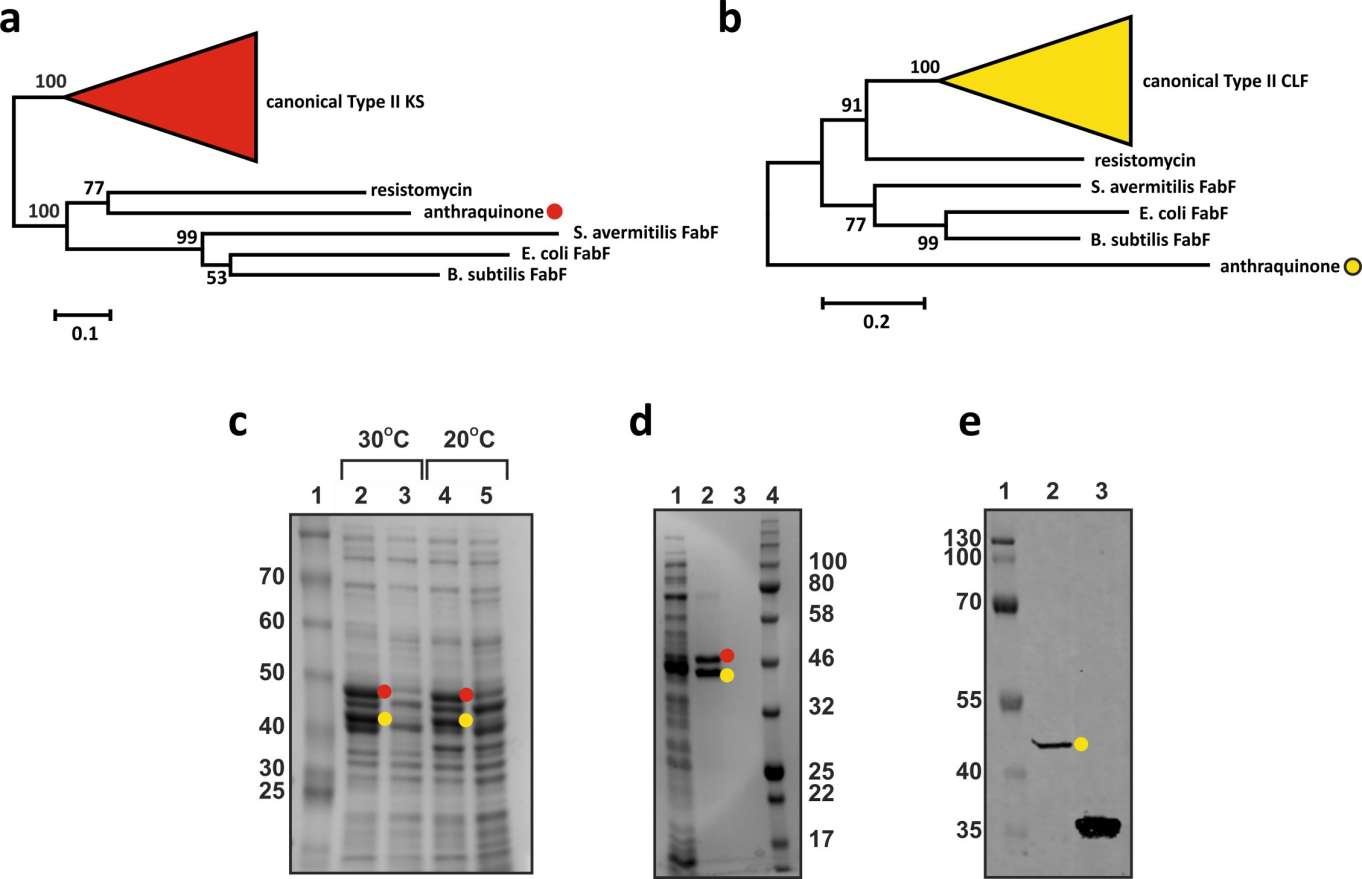
Identification and expression of soluble KS/CLF heterodimers in *E*. *coli*. (a and b) Phylogeny of KS and CLF sequences from a dataset of 58 characterised type II PKSs derived from the MiBIG repository, respectively. The clades representing canonical type II KS and CLF are denoted by red and yellow wedges, respectively. Both alignments include FabF sequences from *S*. *avermitilis*, *E*. *coli*, and *B*. *subtilis*. Red and yellow dots denote His^6^-AntE and AntD, respectively. This colouring is conserved throughout the figure. (c) Denaturing PAGE showing soluble protein extracted from *E*. *coli* BL21(DE3) (lane 1) and *E*. *coli* BL21(DE3) pBbA2k-plumPKS harbouring the mPKS from *P*. *luminescens* (lane 2) induced at 30°C. Lanes 3 and 4 mirror those of 1 and 2; however, they show soluble protein expressed when incubated at 20°C. (d) Denaturing PAGE gel of AntD and E purified by IMAC. Lane 1: protein flow through, lane 2: protein eluted at 50 mM imidazole, lane 3: 500 mM imidazole column wash. (e) Western blot of purified AntDE protein showing signal corresponding to a single AntE band. Lane 1: PAGE ladder, lane 2: purified AntDE protein, and lane 3: His-tagged mCherry (approximately 29 kDa) fusion protein as positive control. All numbers correspond to standard protein ladders and are defined in kDa. Theoretical size of His^6^-AntE and AntD is 42.43 kDa and 46.16 kDa, respectively. CLF, chain length factor; His, Histidine tag; IMAC, immobilised metal ion affinity chromatography; KS, ketosynthase; MiBIG, Minimum Information about a Biosynthetic Gene cluster; mPKS, minimal PKS; PKS, polyketide synthase.

The major challenge in establishing this platform was the identification of soluble mPKS systems for use in *E*. *coli*. Instead of using a trial-and-error approach, we used evolutionary insights into the formation of type II PKSs [12] to identify suitable KS/CLF pairs. Both type I and type II PKSs have been the subject of in-depth evolutionary modelling and phylogenetic analysis in recent years [12–15]. Phylogenetic analysis of large datasets of type II PKSs indicates that canonical type II PKS KS and CLF pairs arose from an ancient KS duplication event, most likely from a FabF-like fatty acid KS [12, 15]. Therefore, the intrinsically soluble FabF protein from *E*. *coli*, sharing a common ancestor with canonical type II PKSs, was used to query candidate KS pairs for heterologous expression in *E*. *coli*. To do so, a dataset of 58 experimentally characterised type II KS sequences was acquired from the Minimum Information about a Biosynthetic Gene cluster (MIBiG) repository [16] and aligned with 3 FabF candidates from *Streptomyces avermitilis*, *Bacillus subtilis*, and *E*. *coli*. KS sequences were chosen to search for homologues, because these represent the catalytic part of the mPKS protein dimer and are more similar to FabF than the passive, and typically more sequence diverse, CLFs. Phylogenetic reconstruction of the sequence alignment identified 2 KSs—RemA from the resistomycin BGC from *S*. *resistomycificus* and AntD from the anthraquinone BGC of *P*. *luminescens* TT01—to associate more closely with the FabF homologues than all other KS sequences acquired from the MiBIG dataset (Fig 3A); both KS and cognate CLF pairs were plausible first candidates for successful heterologous expression in active and soluble form in *E*. *coli*.

Further analysis was conducted on KS/CLF sequences from underexplored phyla. Expectedly, analysis of 2,552 reference genome sequences identified predicted BGCs spanning all 42 classes in the dataset. A phylogenetic tree of characterised actinobacterial and uncharacterised non-actinobacterial KS/CLFs was created to further determine their relationship (S1 Fig, S1 Text, S1 Table).

Biosynthesis of shunt metabolites by the resistomycin mPKS was previously shown to require coexpression of an additional cyclase [17]. This context dependency may limit the chemical diversity accessible through combinatorial biosynthesis of early biosynthetic shunt metabolites, which are shown to have valuable bioactivities. In contrast, the AntD-containing BGC, responsible for biosynthesis of anthraquinone pigments in the nematode symbiont *P*. *luminescens* TT01, was a more attractive candidate because biosynthesis of its anthraquinone core is proposed to be enzymatically congruent with biosynthesis of actinorhodin, the archetypal aromatic polyketide [18]. Though taxonomically *P*. *luminescens* is close to *E*. *coli*, there has been no previous report of expressing the *P*. *luminescens* anthraquinone PKS in *E*. *coli*, and there has been no previous report of using the *P*. *luminescens* anthraquinone mPKS (*antDEF*) in combinatorial biosynthesis for the production of novel aromatic compounds in *E*. *coli*.

### Evaluating solubility and dimer formation of the identified KS and CLF in *E*. *coli*

The entire mPKS complement from *P*. *luminescens*, comprising the KS AntD, CLF AntE, and ACP AntF, were successfully expressed as soluble recombinant proteins in *E*. *coli* BL21(DE3). Furthermore, AntD and AntE were observed as soluble proteins when overexpressed at 20°C and 30°C for >12 h, indicating transcription, translation, and protein folding to be robust (Fig 3C).

Whilst AntD and AntE are both soluble recombinant proteins in *E*. *coli*, the role of AntE as a functional heterodimer partner for AntD was unknown. Sequence features of AntE defy conventions of characterised CLFs: alignment of CLF amino acid sequences in our dataset showed AntE to fringe the clade of canonical CLFs (Fig 3B) and to also lack hallmark and gatekeeper residues that play important roles in polyketide biosynthesis (S1 Text, S2 Fig, S3 Fig, S2 Table). Most notably, the C-terminal third of AntE shows no sequence similarity to any CLF within our dataset; sequence divergence here might indicate that AntE is degenerate and no longer functional.

To examine whether a heterodimeric complex is formed by AntD and AntE, a 6 polyhistidine tag (His^6^) fusion of AntE was coexpressed with AntD in *E*. *coli* BL21(DE3). Protein purified via immobilised metal ion affinity chromatography (IMAC) and visualised by denaturing sodium dodecyl sulfate–polyacrylamide gel electrophoresis (SDS-PAGE) showed 2 distinct bands of similar intensity corresponding closely with the theoretical molecular weight of AntD and His_6_AntE (Fig 3D). Western blotting (Fig 3E) and liquid chromatography tandem mass spectrometry (LC/MS-MS) analysis (Materials and methods) of both bands confirmed that these correspond to AntD and AntE, respectively. Co-purification of AntD with AntE agrees with the assumption that stable AntDE hetereodimers are formed in *E*. *coli*.

In addition to AntDE, the resistomycin KS and CLF (RemA and B) were also soluble recombinant proteins in the *E*. *coli* background (S4 Fig). Expression of RemB in the absence of its KS counterpart resulted in 100% inclusion body formation, indicating that RemAB interactions are necessary for protein solubility. Moreover, a Streptavidin-II (Strep-II)–tagged RemA fusion protein co-purified with His^6^ RemB by IMAC, further confirming RemAB heterodimeric complex formation in vivo.

To test the solubility of the identified KS/CLFs from underexplored phyla, 7 were selected and evaluated (S1 Text, S1 Table). Type II PKS BGCs from *Delftia acidovorans* CCUG274B, *Streptoccocus* sp. GMD2s, *P*. *luteoviolacea*, *Bacillus endophyticus* DSM 13796, Candidatus *Desulfofervidus auxilii*, and *K*. *racemifer* were selected, and the solubility of each corresponding KS and CLF was evaluated as heterodimers and monomers in *E*. *coli* BL21(DE3) by introduction of codon-optimised genes. Three showed solubility in *E*. *coli*: the KS/CLF from *Streptococcus* spp. (SspA/B), *K*. *racemifer* DSM44963 (SOSP1-21 type strain) (KraA/B), and *P*. *luteoviolacea* DSM 6061 (PluA/B) (S5–S8 Figs). The expression conditions were not optimised for the selected KS/CLF pairs; hence, not all enzymes were immediately soluble in *E*. *coli*. However, because 3 enzyme pairs already are soluble, this was encouraging for the future full characterisation of these KS/CLF pairs for further refactoring to greatly expand the aromatic polyketide chemical space currently accessible in *E*. *coli*.

### Testing functionality of the PKS

Expression of soluble heterodimeric aromatic polyketide producing KS/CLF complexes in *E*. *coli* is unprecedented and was the first step towards development of an *E*. *coli*–based combinatorial biosynthetic platform for aromatic polyketides. We next sought to test KS/CLF functionality, and to do so, the AntDE-expressing strains were taken forward, because it was markedly more soluble than the other complexes in *E*. *coli*.

The components of the *P*. *luminescens* anthraquinone mPKS (*antDEF)* were introduced and expressed in *E*. *coli* BL21(DE3) on the plasmid pBbB1a-plumPKS (S3 Table). Previous studies had shown that a *P*. *luminescens* TT01 lacking 1 anthraquinone-associated cyclase accumulated mutactin and dehydromutactin, suggesting that the mPKS synthesises a 16-carbon octaketide primed with an acetyl starter unit [18]. Thus, the expected shunt metabolites formed by the mPKS were the acetyl-primed octaketides SEK4 and SEK4b and their respective dehydrated forms (Fig 2).

**Fig 4:**
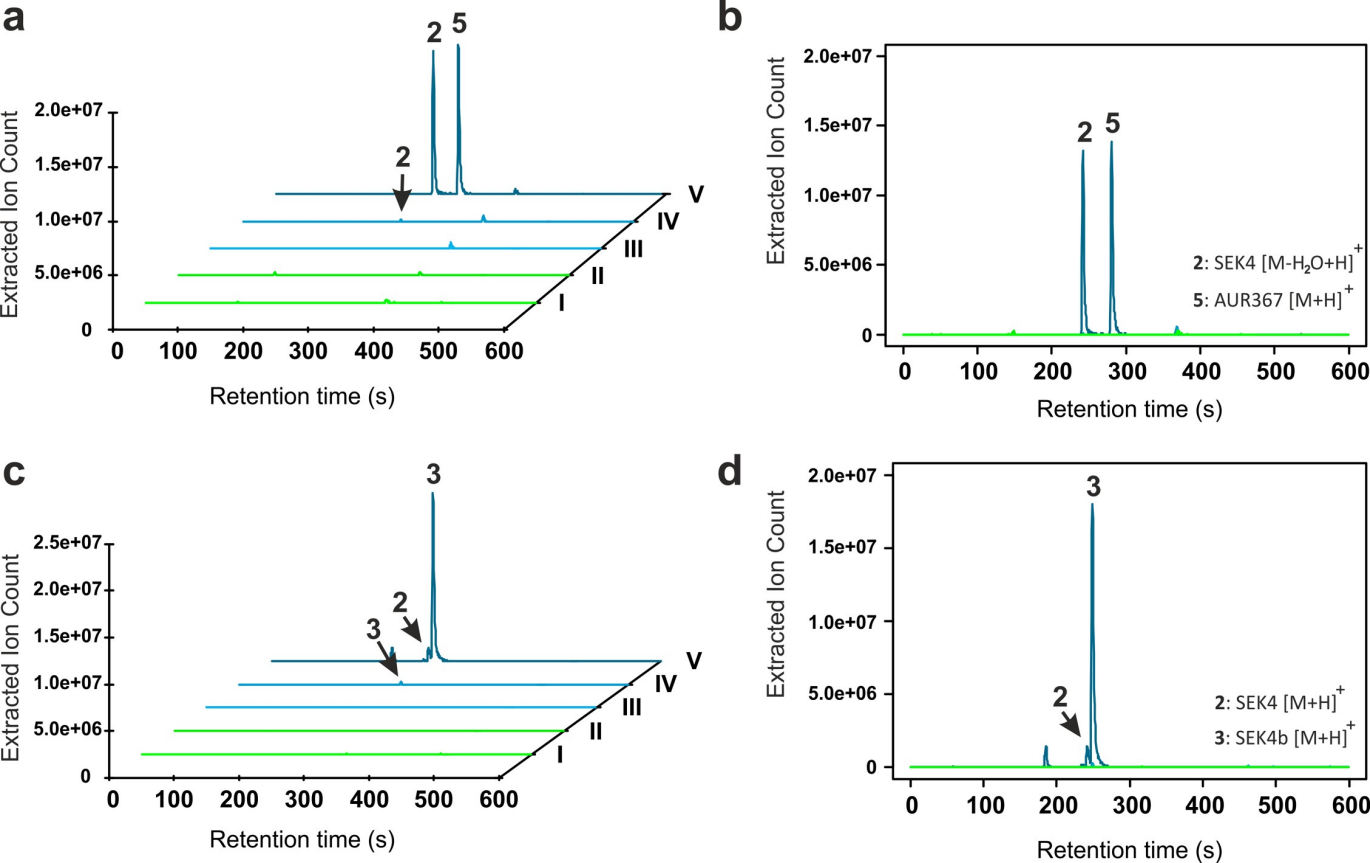
Expression of AntDEFBG in *E*. *coli*. (a and b) Three-dimensional and two-dimensional EICs of all observable masses between 301.0661 and 301.0714 *m/z*. The theoretical mass of expected shunt metabolites AUR367 (5) and SEK4 (2) is [M-H_2_O+H]^+^ 301.07067. (c and d) Three-dimensional and two-dimensional EICs of all observable masses between 319.0764 and 319.086 *m/z*. The theoretical mass of expected shunt metabolites SEK4 (2) and SEK4b (3) is [M+H]^+^ 319.08099, 15 ppm tolerance. Samples I through V are filtered supernatant from culture of (I) *E*. *coli* BL21, (II) *E*. *coli* BL21 pBbB1a-GFP pACYCDuet-1 (empty vector control), (III) *E*. *coli* BL21 pBbB1a-plumPKS pACYCDuet-1 (AntDEF), (IV) *E*. *coli* BL21 pBbB1a-plumPKS pACYC93 (AntDEFB), and (V) *E*. *coli* BL21 pBbB1a-plumPKS pACYC8893 (AntDEFBG). Extracted ion count was normalised to final cell density (OD_600_). All bolded numbers correspond to Fig 2. EIC, extracted ion chromatogram; OD_600_, optical density measured at 600 nm.

However, expression of the mPKS (*antDEF*) alone did not produce any detectable masses corresponding to SEK4/SEK4b (Fig 4, sample III). A lack of detectable octaketides suggested *E*. *coli* endogenous 4′ phosphopantetheinyl transferases (PPTases)—AcpS and EntD—could not efficiently functionalise ACP (AntF) with a 4′ phosphopantetheine arm necessary for activity. Alternative auxiliary enzymes, the anthraquinone-associated PPTase (*antB*) and Coenzyme A (CoA) ligase (*antG*), were required to carry out this posttranslation modification, which resulted in detectable biosynthesis of molecules putatively identified as SEK4, SEK4b, and AUR367 (Fig 4, sample IV): MS-MS fragmentation patterns of the putatively assigned octaketides were consistent with values reported in the literature [19] (S9 Fig). Interestingly, in the absence of AntG, the putatively assigned CoA ligase, a 13-fold decrease in SEK4 and SEK4b relative ion intensities was observed compared with the *antDEFBG*-expressing strain. This reduction in metabolite concentration suggests that AntF (ACP) is charged by the anthraquinone-associated PPTase (AntB) and that AntG functions as an acyl-ACP synthetase and directly and selectively loads *holo-*AntF with an acyl-CoA substrate [20], thus being a necessary component of the mPKS (Fig 1). However, we cannot rule out the possibility that the AntG additionally has the originally suggested CoA ligase activity.

### Exploring end-compound production of the anthraquinone biosynthetic gene cluster

In addition to the mPKS (extension units) and auxiliary CoA ligase and PPTase (priming units), primary tailoring enzymes are common to all aromatic PKS pathways and form principal components of the tailoring unit (Fig 2). The anthraquinone biosynthetic gene cluster of *P*. *luminescens* is no different and encodes 4 other enzymes with putative assigned functions, including a C9-ketoreductase (KR), 2 cyclases, and a hydrolase/peptidase. The full complement of biosynthetic genes is predicted to produce 1,3,8-trihydroxyanthrone (Fig 2, compound 12), and—in a similar fashion to aurachin biosynthesis [21, 22]—additional tailoring enzymes responsible for further modification of the polyketide core are thought to exist in extension clusters situated elsewhere on the *P*. *luminescens* genome. Accordingly, we sought to determine whether the extended biosynthetic pathway was functional in *E*. *coli* and able to produce the expected anthrone (Fig 2 compound 12). To do so, the entire anthraquinone cluster (accession no. BX470251.1, MIBiG no. BGC0000196, 9,166 bp) was introduced into pACYCDuet-1 and expressed in *E*. *coli* BL21(DE3). The exometabolome of the resulting strain was analysed for the production of key expected octaketide shunt metabolites as well as plausible octaketide end products (S2 Text, S10–S12 Figs).

**Fig 5:**
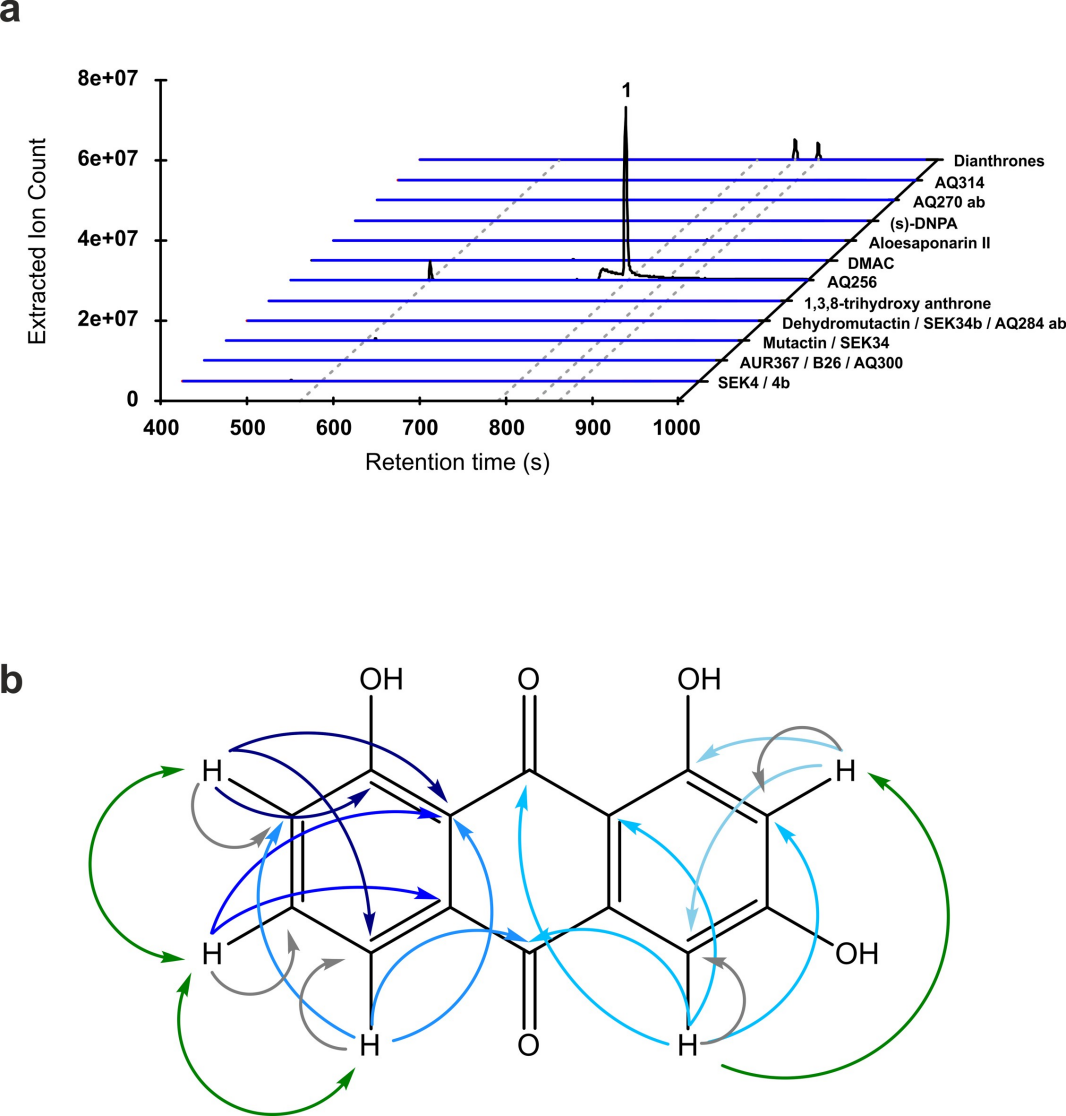
Anthraquinone identification and characterisation. (a) EIC in negative ionisation mode for shunt metabolites described in Fig 2A, in addition to anthraquinones produced by *P*. *luminescens* TT01 and predicted pathway end compounds. All EICs represent theoretical mass for [M-H]^−^ ± 5 ppm. Red, blue, and black lines represent EICs of *E*. *coli* BL21 (host control), *E*. *coli* BL21 pACYCDuet-1 (plasmid control), and *E*. *coli* BL21 pACYCAnthraquinone normalized to final cell density. EICs displaying masses from positive ionisation mode are detailed in S17 Fig. (b) Schematic diagram showing intramolecular couplings between nuclei of *E*. *coli*–produced AQ256, which were determined by COSY (green), HSQC (grey), and HMBC (blue scale) two-dimensional NMR spectroscopies. Coupling constants are detailed in Materials and methods. COSY, correlation spectroscopy; DMAC, 3,8-dihydroxy-methylanthraquinone carboxylic acid; EIC, extracted ion chromatogram; HMBC, heteronuclear multiple bond correlation; HSQC, heteronuclear single quantum correlation; NMR, Nuclear magnetic resonance.

Masses corresponding to 1,3,8-trihydroxyanthrone (compound 12) were only observed at trace levels using high-resolution MS in both positive and negative electrospray ionisation mode (S10 and S11 Figs). Anthrone natural products have previously been shown to form their cognate anthraquinone or dianthrone either enzymatically or via spontaneous oxidation [23]. Should the trihydroxylated anthrone follow the same oxidation pathways in *E*. *coli*, or during the extraction process, masses corresponding to AQ256 and 1,3,8-trihydroxydianthrone (Fig 2, compounds 1 and 13) would be expected. Both oxidised metabolites were identified and characterised. This targeted search led to the observation of AQ256 as the major product of the extended anthraquinone BGC (Fig 5, S10 Fig), which was fully characterized by nuclear magnetic resonance (NMR) spectroscopy (S14 Fig) where NMR spectra are consistent with existing literature [24], high-resolution mass spectrometry (MS) and tandem MS (MS-MS), where fragmentation patterns for AQ256 follow those of other anthraquinones (S10 Fig), and UV-visible (UV-Vis) absorbance in agreement with similar anthraquinones (λ_max_: 244, 265, 284, and 434 nm) (S15 Fig). From a large-scale cultivation of *E*. *coli* BL21(DE3) harbouring plasmid with the entire anthraquinone cluster-pACYCAnthraquinone, grown in 6 L of lysogeny broth (LB) medium, 15 mg of pure (>95%) AQ256 was obtained, corresponding to a production yield of approximately 2.5 mg/L. Interestingly, while quinone formation is proposed to be catalysed by the *plu*0947 gene product in *P*. *luminescens* TT01 [18], the absence of *plu*0947, ActVA-ORF5/ActVB, or ActVA-ORF6 homologues within the *E*. *coli* BL21 genome indicates that the quinone-forming oxygen is either introduced by an unknown alternative endogenous enzyme or through nonenzymatic oxidation as proposed for cladofulvin biosynthesis [23]. More extensively modified anthraquinones isolated from *P*. *luminescens* TT01 [18] were not identified as end compounds in the engineered *E*. *coli* strains, consistent with the absence of additional cognate tailoring enzymes.

Masses corresponding to two 1,3,8-trihydroxy-dianthrones were also identified and characterised by high-resolution MS (S10 Fig). MS-MS spectra of both dianthrones show fragmentation to occur at the C10 − 10′ bond forming anthrone radicals (S2 Text, S13 Fig): a hallmark fragmentation pattern of a wide variety of glycosylated and aglycone dianthrones [25]. Additionally, the UV-Vis absorbance spectra of both putative dianthrones showed similarities to emodin dianthrone [26, 27] with λ_max_ at 359, 263, and 217 nm and λ_max_ at 358 and 275, respectively (S13 Fig). It is plausible that the 2 metabolites correspond to *trans* and *meso* dianthrones; however, full characterisation by NMR was not possible because neither compound was present in sufficient quantities.

### Evaluation of a plug-and-play scaffold

#### Complementation of the C9-KR

A biosynthetic route to 2 pharmaceutically important octaketide scaffolds—anthraquinones and dianthones—was now established using type II PKSs in *E*. *coli*; however, to generate large libraries of aromatic polyketide derivatives, a plug-and-play scaffold is necessary, in which functionally diverse biosynthetic genes from a range of phylogenetically distant organisms can be substituted and added successfully (Fig 2B).

During aromatic polyketide biosynthesis, the growing polyketide chain is tethered to an ACP, in this case AntF. To reach compound maturation, the ACP-tethered polyketide chain must be sequentially delivered to enzymes within the biosynthetic pathway (Fig 1); this is facilitated by specific protein–protein interactions. Therefore, for a plug-and-play platform to function, AntF must successfully form these interactions with a multitude of non–cluster-associated tailoring enzymes [28]. The promiscuity of AntF is therefore the major bottleneck and the key determinant in the success of the AntA-I cluster as a generic platform for aromatic polyketide derivatisation in *E*. *coli*.

To elucidate whether uncommon ACP characteristics hamper AntF from functioning outside of the *ant* BGC, the cluster-associated KR, AntA, was functionally replaced with ActIII, a C9-KR homologue from the phylogenetically distant *Streptomyces coelicolor* actinorhodin (*act*) BGC. Two vectors comprising the *ant* cluster but lacking *antA* were constructed; the first replaced *antA* with a fully refactored *actIII* using *E*. *coli* codon preference, and the second harboured a modified wild-type *actIII* with two 5′ synonymous mutations, C to G (6th nucleotide from ATG) and G to C (9th nucleotide from ATG), which has been shown to express successfully in *E*. *coli* previously [29].

**Fig 6:**
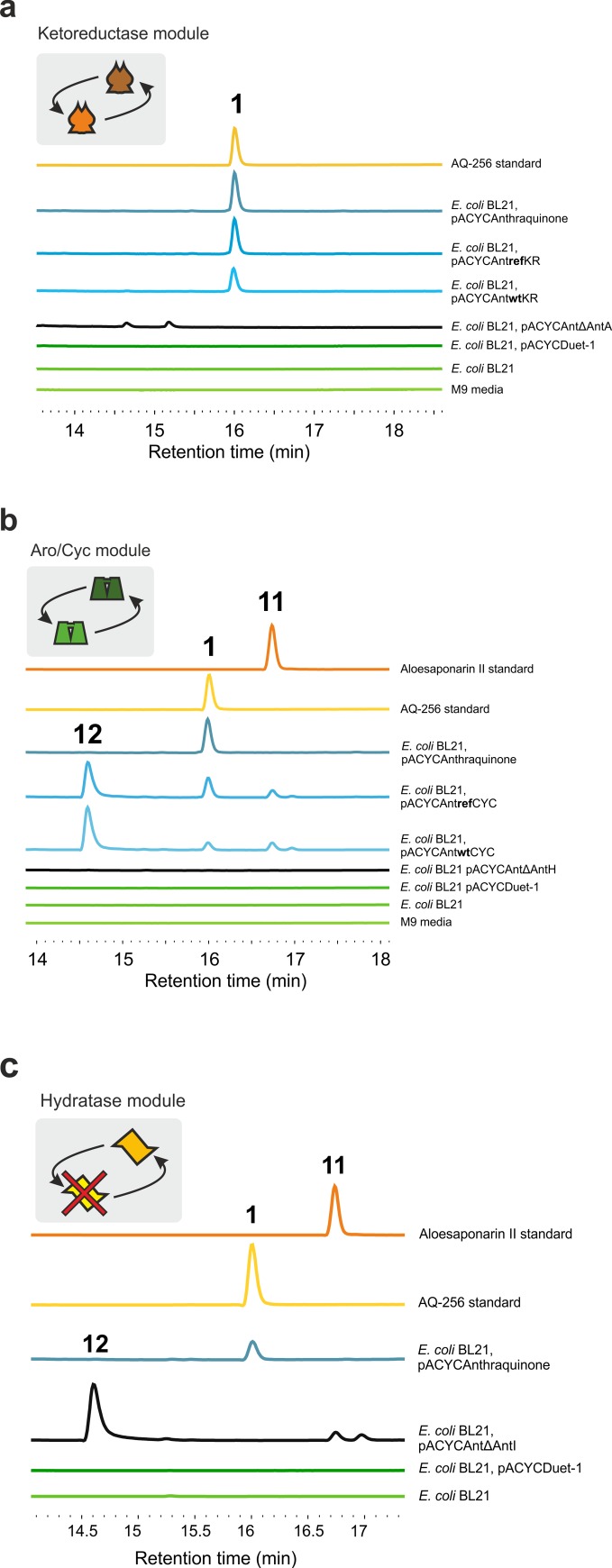
Complementation of the anthraquinone BGC with actinorhodin components. (a) Typical exometabolome HPLC profiles of *E*. *coli* BL21, *E*. *coli* BL21 expressing empty vector pACYCDuet-1, KR *antA* knockout mutant pACYCAntΔAntA, wild-type *Sco*5086 KR complemented plasmid pACYCAntwtKR, refactored *Sco*5086 KR complemented plasmid pACYCAntrefKR, and pACYCAnthraquinone compared with fully characterised AQ256 standards and M9 growth media at 434 nm. The UV-Vis spectrum for peaks designated 1 are as follows: AQ256 standard λ_max_ at 216, 264, 282, 434, and 583 nm; *E*. *coli* BL21 pACYCAnthraquinone λ_max_ at 215, 263, 283, and 434 nm; *E*. *coli* pACYCAntrefKR λ_max_ at 215, 283, and 435; and *E*. *coli* BL21 pACYCAntwtKR λ_max_ at 216, 282, 435, and 585 in agreement with AQ256 (S15 Fig). Spectra were limited to 215–600 nm. (b) Typical exometabolome HPLC profiles of *E*. *coli* BL21, *E*. *coli* BL21 expressing empty vector pACYCDuet-1, ARO/CYC knockout mutant pACYCAntΔAntH, wild-type *Sco*5090 ARO/CYC complemented plasmid pACYCAntwtCYC, refactored *Sco*5090 ARO/CYC complemented plasmid pACYCAntrefCYC, and pACYCAnthraquinone compared with standards as above. Compound reference numbers are as described in Fig 2: 1: AQ256; 11: Aloesaponarin II; and 10: DMAC. The UV-Vis spectrum for the 3 most abundant peaks are as follows: 1: λ_max_ at 216, 264, 282, 434, and 583 nm in agreement with AQ-256 (S15 Fig); 10: λ_max_ at 217, 408, and 650 nm; 11: λ_max_ at 214, 277, 409, and 582 nm, with tR of 16.004 min (960 s), 14.62 min (877.2 s), and 16.742 min (1004.5 s), respectively. (c) Typical exometabolite HPLC profile of *E*. *coli* BL21, *E*. *coli* BL21 expressing empty vector pACYCDuet-1, an α/β hydratase knockout mutant pACYCAntΔAntI, *E*. *coli* BL21 harbouring pACYCAnthraquinone, and anthraquinone standards. All numbering and λ_max_ Figs are as in panel b. ARO, aromatase; BGC, biosynthesis gene cluster; CYC, cyclase; DMAC, 3,8-dihydroxy-methylanthraquinone carboxylic acid; HPLC, high resolution liquid chromatography; KR, ketoreductase; tR, retention time; UV-Vis, UV-visible.

Substitution of *antA* with *actIII* restored the wild-type phenotype and AQ256 production in *E*. *coli* BL21 when expressed from either refactored or wild-type *actIII* gene sequences (Fig 6A), whilst removal of *antA* abolished AQ256 biosynthesis (Fig 6A, S16 Fig), indicating that AntF successfully delivers biosynthetic intermediates to and from ActIII in vivo and that there are no uncommon ACP characteristics that hamper AntF from functioning outside of the *ant* BGC. Furthermore, no moonlighting activity from other biosynthetic enzymes or *E*. *coli* endogenous KRs was observed. Moreover, in the *ΔantA* host, the expected shunt metabolites SEK4 and SEK4b accumulated at much higher intensities compared to cultures expressing the entire gene cluster when normalised to final cell density consistent with a C9-KR metabolic bottleneck (S16 Fig).

#### Cyclase/aromatase complementation

In addition to functionally replacing AntA by ActIII, the enzymatic function of the structurally unique tridomain cyclase/aromatase, AntH, was successfully substituted by the well-characterised didomain cyclase ActVII, from the *act* BGC. Two additional constructs were built following the same strategy as the KR replacement such that the first construct substituted *antH* with a refactored *actVII* and the second with wild-type *actVII*.

Functional replacement of AntH by ActVII (derived from either the refactored or wild-type *actVII*) restored AQ256 biosynthesis, not observed in the *ΔantH* host (Fig 6B, S17 Fig) evidencing the promiscuity of AntF to interface with structurally different enzymes in vivo. Interestingly, in both ActVII complemented strains actinorhodin shunt metabolites aloesaponarin II and 3,8-dihydroxy-methylanthraquinone carboxylic acid (DMAC) were also observed (Fig 6B, S17 Fig). Identification of all 3 end products indicates that the maturing polyketide chain successfully undergoes congruent reduction, aromatisation, and cyclisation to form a common bicyclic intermediate before differing in mechanism of chain release and final ring cyclisation. Deletion of *antI*, the hydrolase/peptidase, proposed to be involved in acyl-ACP release or final ring formation, confirmed this observation. *E*. *coli* deficient in the AntI, but expressing *antA-H*, no longer produced AQ256 but rather produced aloesaponarin II and DMAC as end products (Fig 6C). This concludes AntI to be the branch point between anthraquinone and dianthrone formation and BIQ biosynthesis. The use of this truncated *ant* BGC supplementated with late BIQ biosynthetic enzymes, might produce actinorhodin, granaticin and other BIQs in *E*. *coli*.

#### Expanding the aromatic polyketide chemical space accessible in *E*. *coli*

**Fig 7:**
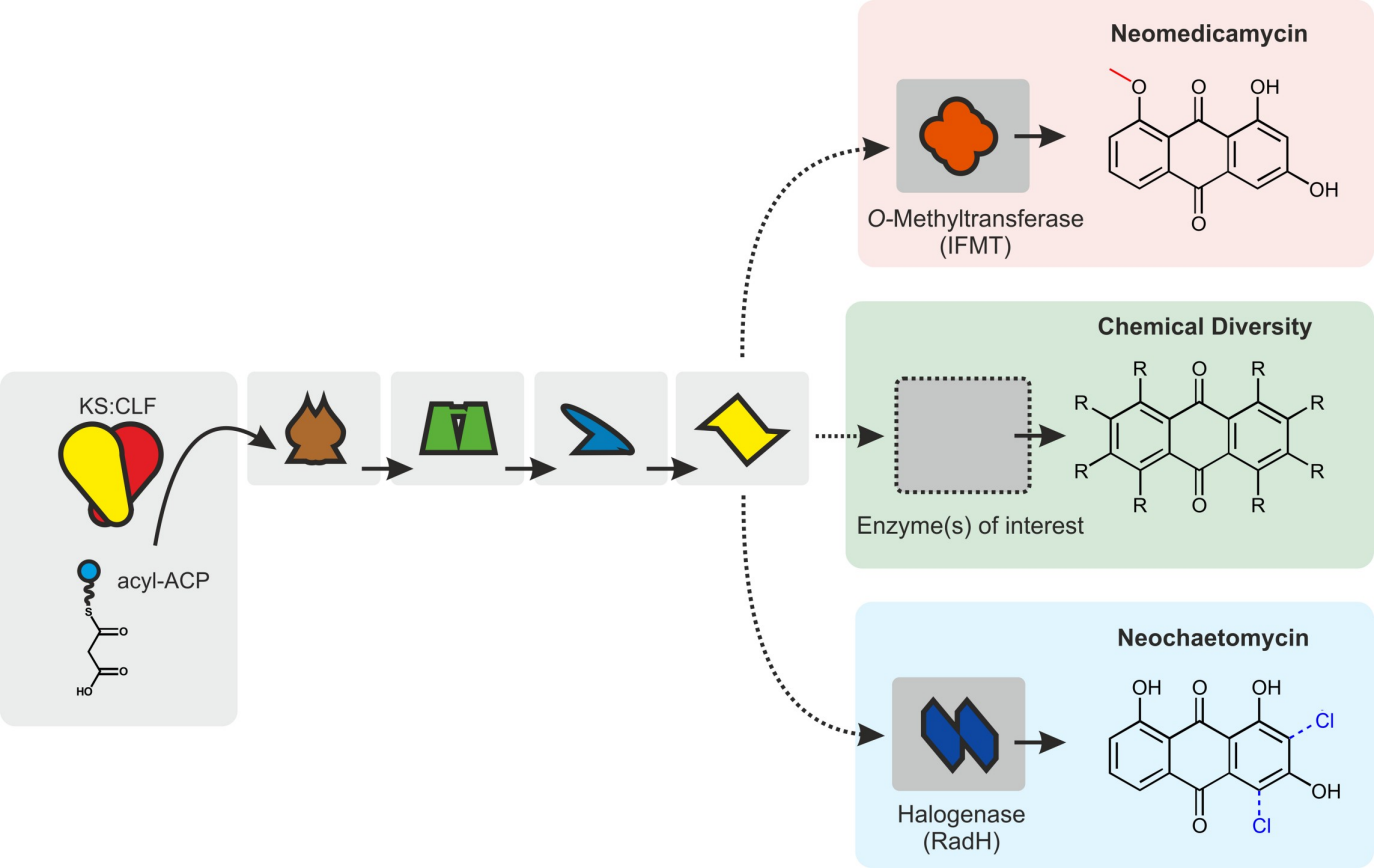
Introducing chemical diversity through supplementation and substitution of secondary tailoring enzymes. A schematic representation of an AntA-I plug-and-play biosynthetic scaffold extended with enzymes sourced from a range of organisms. Metabolites produced through addition of *O*-methyltransferase (from *M*. *truncatula*) and RadH (from *Chaetomium chiversii*) are shown in red and blue boxes correspondingly. Enzymes within AntA-I plug-and-play core biosynthetic pathway are represented in light grey boxes. Additional non-cognate secondary tailoring enzymes are shown in darker grey boxes. Dotted lines represent deviation away from the natural AQ256 biosynthetic pathway. ACP, acyl carrier protein; CLF, chain length factor; KS, ketosynthase.

The ability to functionally substitute primary tailoring enzymes in a plug-and-play fashion is an important step to test the robustness of the anthraquinone-derived biosynthetic pathway; however, the chemical space accessible in doing so is well-trodden and modest [30]. To exemplify the wider utility of the AntA-I plug-and-play scaffold, we used previously characterised secondary tailoring enzymes to produce new compounds. *P*. *luminescens* TT01 produces a suite of modified anthraquinone in addition to AQ256 [18], the majority of which comprise C1 or C3 methoxy groups. The methyltransferase(s) performing these reactions remain unknown. We used an *O-*methyltransferase (*ifmt*) from *Medicago truncatula* in an attempt to complement C1 or C3 *O*-methylation in *E*. *coli*; however, this resulted in biosynthesis of a new C8 methoxy-substituted compound, 1,3-dihydroxy-8-methoxyanthraquinone, named neomedicamycin (Fig 7, S3 Text, S17 and S18 Figs). *E*. *coli* BL21(DE3) harbouring *antA-I* and *ifmt* was grown in 4.8 L of LB for large-scale neomedicamycin production and approximately 0.5 mg of purified product, corresponding to an obtained production yield of 1.04 mg/L. Neomedicamycin was characterised by UV-Vis spectroscopy, high-resolution MS, and ^1^H, correlation spectroscopy (COSY), heteronuclear single quantum correlation (HSQC), and heteronuclear multiple bond correlation (HMBC) NMR spectroscopy, and unambiguous assignment of the methoxy group at C8 was facilitated by solving the crystal structure (Fig 7, S3 Text, S18 Fig). To our knowledge, neomedicamycin has not previously been described in nature [31].

Through addition of alternative enzymes to the AntA-I pathway, we also modified AQ256 with the promiscuous flavin-dependent halogenase RadH, from the *C*. *chiversii* fungus [32], and formed a monochlorinated AQ256 analogue—1,3,8-trihydroxy-monochloroanthraquinone—as determined by UV-Vis spectroscopy, high-resolution MS and ^1^H NMR spectroscopy (S19 Fig), which to our knowledge is also a new chemical entity, here named neochaetomycin. *E*. *coli* BL21(DE3) harbouring *antA-I* and *radH* was grown in 0.15 L of M9 for neochaetomycin isolation and gave a yield of approximately 0.73 mg/L. Chlorinated anthraquinone derivatives are known ATP citrate lyase (ACL) inhibitors, modulating de novo fatty acid synthesis in mammalian cells [33]. ACL up-regulation is observed in cancer cell lines, including chemoresistant colororectal cell lines [34] and cancer stem-like cells [35]; therefore, new chlorinated anthraquinones might be of interest as innovative chemotherapy agents. Furthermore, chlorinated anthraquinones are interesting antibiotic agents. Chloro-emodin shows more potent bactericidal activities against multidrug-resistant gram-positive pathogens than emodin and matches the activity of some commercially available antibiotics [36]. A biosynthetic route to chloro-emodin is yet to be described: the plug-and-play scaffold described here could easily be used to prototype this.

## Discussion

We show that bacterial PKSII systems can be successfully refactored in *E*. *coli*. The active expression of bacterial mPKS (KS, CLF, ACP enzymes) was previously not possible in this bioengineering workhorse species. However, by functionally expressing a type II mPKS from *P*. *luminescens* in *E*. *coli*, we overcome this limitation. We further show that this mPKS can be used in combination with enzymes from actinobacteria, plant, and fungi to produce a diversity of type II polyketides, establishing the general applicability of this platform. Potentially, the 3 other mPKSs described in this manuscript could also be functional as alternative platform PKSs, as we have shown them to be soluble in *E*. *coli* as well. As a result, it is no longer necessary to use the intractable mPKSs from actinobacteria when intending to produce type II polyketides in *E*. *coli*. Instead, we can now use, e.g., the *P*. *luminescens* mPKS to produce type II polyketides in *E*. *coli* through a mix-and-match strategy, combining the mPKS platform with tailoring enzymes from actinomycetes and other organisms.

The ability to synthesise aromatic polyketides in *E*. *coli* opens up exciting avenues for the rapid and versatile diversification of novel bioactive compounds by prototyping multiple biosynthetic pathways. Through establishing a versatile and robust biosynthetic production line to type II polyketides in *E*. *coli* it is now finally possible to perform multiple design–build–test–learn iterations for this system in a highly automated fashion—this was previously unachievable in often poorly characterised, and genetically intractable, native Actinobacterial hosts [37] from which most type II polyketides are characterised. Generation of a type II PKS exploration system in *E*. *coli* facilitates faster polyketide engineering, leading to greater accessible chemical diversity and expediting drug lead identification. Promising biosynthetic pathways can then be refined and translated into optimised bioproduction chassis for commercial exploitation.

Whilst we characterise minimal, native, and extended polyketide biosynthetic pathways in *E*. *coli* and produce 2 new chemical entities, the success of this plug-and-play platform is yet to be fully realised; however, it already meets the 2 key criteria for a good drug discovery platform: the underlying enzymology comprises discrete and dissociated biosynthetic parts [38, 39], which are well suited to combinatorial biosynthesis, arguably more so than their type I counterparts. And aromatic polyketides—the products of these pathways—include well-known and diverse bioactive specialised metabolites that are heavily used in the clinic [40, 41]. Furthermore, due to the pharmaceutical importance of aromatic polyketides, a wealth of literature already describes how to modify a variety of facets governing chemical diversity, including polyketide chain length [6], starter unit selection [42], cyclisation patterns [43], and addition, substitution, or deletion of biosynthetic genes in type II BGCs; all of these alterations can now be introduced into *E*. *coli* and harnessed for combinatorial exploration using existing high-throughput pathway assembly platforms [44].

By opening the field of type II polyketide biosynthesis to the high-throughput synthetic biology toolbox available in *E*. *coli*, and taking advantage of the plug-and-play platform, it is now possible to generate large libraries of chemical diverse aromatic polyketides in a highly automated manner starting from pharmacologically privileged scaffolds holding the potential to unlock new promising bioactivities.

## Materials and methods

### Bacterial strains and culture conditions

*E*. *coli* DH5α was used for routine cloning and plasmid preparation and maintenance. *E*. *coli* BL21(DE3) was used for expression of all recombinant proteins described herein with the exception of AntDE purification, in which *E*. *coli* NiCo21(DE3) was used. For protein purification, *E*. *coli* NiCo21(DE3) or BL21(DE3) was cultured in LB at 37°C, 180 rpm, supplemented with appropriate antibiotics and induced with isopropyl β-D-1-thiogalactopyranoside (IPTG) at OD_600_ 0.5–0.6 before reducing incubation temperature to 16°C for a further 16 h. For compound isolation, *E*. *coli* BL21(DE3) was cultured in LB for large-scale production of AQ256 and neomedicamycin, or minimal media as 2 mM MgSO_4_, 100 μM CaCl_2_, 238.8 μM Na_2_HPO_4_, 110.2 μM KH_2_PO_4_, 93.47 μM NH_4_Cl, 42.78 μM NaCl, 0.4% glucose in distilled H_2_O with appropriate antibiotics. Cultures were incubated at 37°C, 180 rpm, to OD_600_ 0.35–0.4 and induced with 50 μM IPTG, for AQ256 and octaketide shunt metabolites before reducing the incubation temperature to 20°C for 3 to 5 d. *P*. *luminescens* TT01 was kindly provided by Dr. Ralf Heermann of Ludwig-Maximilian University, Munich. *P*. *luminescens* TT01 cultures were inoculated in casein-peptone soymeal-peptone broth and routinely grown at 28°C. Working antibiotic concentrations were as follows: carbenicillin: 50 μg ml^−1^; kanamycin: 25 μg ml^−1^; and chloramphenicol: 25 μg ml^−1^.

### DNA isolation

Genomic DNA (gDNA) from *P*. *luminescens* TT01 and *S*. *coelicolor* M145 was isolated from cultures grown to an OD_600_ of 1 and OD_450_ of 0.8, respectively, using a standard phenol chloroform DNA purification protocol and validated using routine PCR amplification. All primers used in this study are detailed in S4 Table. All vectors used in this study are listed in S5 Table. CloneAmp HiFi PCR Premix (TaKaRa, Kusatsu, Japan) was used for all routine PCR amplification; for PCR products over 10 Kb PrimeSTAR Max DNA polymerase (TaKaRa, Kusatsu, Japan) was used. All PCR products and restriction endonuclease (RE) digests were purified using the MinElute PCR purification kit (Qiagen, Hilden, Germany) as per manufacturer instructions. All ligations were performed using the Rapid DNA ligation kit (Roche, Basel, Switzerland) as described by the manufacturer. All REs used in this study were obtained from New England Biolabs (NEB; Ipswich, MA), and digests were performed for 1 h at 37°C unless stated otherwise.

### Plasmid construction and refactoring

To construct the first KS/CLF expression, vector pBbA2K-RFP was digested with EcoRI-HF and XhoI; the larger DNA fragment comprising the vector backbone was PCR purified. Primers EcoRI_Plu_for and Plu4189_XhoI_R were used to amplify a 2,779 bp fragment encoding *plu*4191, *plu*4190, and *plu*4189 from *P*. *luminescens* TT01 gDNA by PCR, which was purified as above and ligated into the empty pBbA2k vector using T4 DNA ligase. The same procedure was followed to construct the BBR1 ori, T7 promoter, and ampicillin-resistant backbone mPKS expression vector pBbB1a-plumPKS. Aromatic polyketide KS and CLF genes are almost exclusively translationally coupled, and this is assumed to be the case for *plu*4191 and *plu*4190 due to the start:stop codon overlap. Transcriptional coupling has been proposed to colocalise proteins transcribed from the same polycistronic mRNA and may aid dimer formation; as such, the native operon architecture of *plu*4191, 90, and 89, which is equivalent to KS, CLF, and ACP, respectively, was retained in the pBbA2k expression vector. To construct the his^6^-AntE and AntD protein purification, vector pETDuet419091 *plu*4191 was PCR amplified from *P*. *luminescens* TT01 gDNA using primers Plu4191_for_BglII and Plu4191_rev_KpnI, purified as above and digested with BglII and KpnI-HF. The digested *plu*4191 fragment was ligated into pETDuet-1 also digested with BglII and KpnI-HF to form pETDuet4191. *Plu*4190 was amplified from *P*. *luminescens* TT01 gDNA using plu4190_for_EcoRI, which removed the start ATG, and Plu4190_rev_PstI. The fragment was purified as above and digested using EcoRI-HF and PstI-HF before ligating into pETDuetplu4191 linearised with EcoRI-HF and PstI-HF to form the His^6^-*plu*4190 fusion vector pETDuetplu419091. To express recombinant AntB and AntG, the PPTase and CoA ligase from the anthraquinone BGC, *plu*4193, and *plu*4188 sequences were amplified from *P*. *luminescens* TT01 gDNA using primers Plu4193_for_NcoI_untagged, Plu4193_rev_HindIII, Plu4188_for_NdeI, and Plu4188_rev_XhoI, respectively, before purification, digestion with NcoI and HindIII, and NdeI and XhoI correspondingly and ligation into pACYCDuet-1 using appropriate REs to form both pACYCPlu4188 firstly and pACYCPlu418893 subsequently. Neither *plu*4193 nor *plu*4188 was tagged. The p15A ori enabled coexpression of AntB and G with the mPKS of pBbB1a-plumPKS.

The entire complement of genes responsible for anthraquinone biosynthesis were cloned into pACYCDuet-1. This enables further plasmids with compatible origins of replication to be easily introduced when derivatising the end compound in a combinatorial fashion. To construct this vector, a fragment comprising *plu*4192, 93, and 94 was first cloned into pACYCDuet-1 multiple cloning site (MCS)-2 after PCR amplification from *P*. *luminescens* TT01 gDNA using primers Plu4194_for_NdeI and Plu4192_rev_XhoI. Both the fragment and pACYCDuet-1 vector were digested with NdeI and XhoI before ligation, as above, to form pACYCDuetPlu4192-94. The 6 remaining genes, *plu*4186–91, were cloned into pACYCDuet-1 MCS-1 in the same manner except using primers Plu_for_EcoRI and AnthraquinoneBGC_Rev_PstI for the PCR amplification forming the 13.6 Kb pACYCAnthraquinone vector.

To generate the KR complementation vector, the anthraquinone KR, *plu*4194, was swapped with *sco*5086, the C9 KR from the actinorhodin BGC; here, pACYCAnthraquinone was linearised with primers KR_Swap_IF and Plu4194_rev_InFusion, which both read outwards of *Plu*4194 removing most of the *plu*4194 CDS from the linear vector. The resulting 13.6 Kb fragment was purified by ethanol precipitation. A 767 bp DNA fragment encoding the wild-type *sco*5086 sequence was PCR amplified from *Streptomyces coelicolor* M145 using primers Sco5086_for_IF Sco5086_rev_IF introducing 2 synonymous mutations in the 5′ of the sequence. These primers added 15 bps of sequence homologous to each end of the linearised pACYCAnthraΔ*plu*4194 to the *sco*5086-containing sequence and enabled plasmid construction by In Fusion (Clonetech, Mountain View, CA), as per the manufacturer’s instruction, forming pACYCAntwtKR. The anthraquinone KR, *plu*4194, and downstream CDS PPTase, *plu*4193, are transcriptionally coupled with a start-stop codon overlap. The PPTase ribosome binding site, therefore, is within the 3′ end of *plu*4194; fortuitously, the N-terminal amino acid sequence of both ActIII and AntA are identical. The primer plu4194_rev_InFusion binds a DNA sequence within the region encoding the identical sequence N-terminal amino acid sequence and maintains the putative PPTase Shine-Dalgarno. The same procedure was followed for the introduction of the refactored actinorhodin KR; however, Ref_Sco5086_IF_for and Ref_SCO5086_IF_rev were used as primers to amplify a codon-optimised *sco*5086 sequence from pG9m-2-ActKRRef to form pACYCAntrefKR. Additionally, pACYCAnthraΔKR was constructed as a KR negative control by linearising pACYCAnthraquinone via PCR as above using primers Plu4194_del_IF_for and KR_Swap_IF_For followed by DNA assembly using NEBuilder HiFi DNA Assembly Master Mix (NEB) following the manufacturer’s instructions. The same process was undertaken to construct *sco*5090 complementation vectors. Once more, pACYCAnthraquinone was linearised with primers plu4187_replace_fw and plu4188_rev both removing *plu*4187 to form the 12,169 bp linearised vector and purified by ethanol precipitation. A 989 bp DNA fragment encoding the wild-type *sco*5086 sequence was PCR amplified from *S*. *coelicolor* M145 using primers Sco5090_for_IF Sco5090_rev_IF and purified using the MinElute Qiagen (Hilden, Germany) PCR purification kit. The vector pACYCAntwtCYC was subsequently constructed via In Fusion DNA assembly from purified *sco*5090 DNA fragment and linearised pACYCAnthraΔ*plu*4187. To introduce the refactored actinorhodin CYC/ARO, the same method was followed; however, Ref_Sco5090_IF_for and Ref_Sco5090_IF_rev were used as primers to amplify a codon-optimised *sco*5090 sequence from pG9m-2-ActARO/CYCRef forming pACYCAntrefCYC/ARO. The *sco*5090 knockout plasmid pACYCAntΔAntH was generated by linearisation of pACYCAnthraquinone using primers Plu4187_delta_for and Plu4187_delta_rev, purification of the linear DNA fragment by ethanol precipitation, and DNA assembly using In Fusion as described above. Plasmids pACYCAntΔAntC and pACYCAntΔAntI were also generated by linearisation of pACYCAnthraquinone using primers Plu4192Δ_for and Plu4192Δ_rev, and plu4186Δ_for and plu4186Δ_rev, respectively, followed by In Fusion DNA assembly as above. All codon-optimised genes were designed using Gen Optimiser and manufactured by Gen9 (Massachusetts, US).

The gene for IFMT (also known as IOMT 3, SAM dependent isoflavone 7-*O*-methyltransferase, GenBank: AAY18582.1) from *M*. *truncatula* was codon optimised for *E*.*coli* expression and synthesised by GenScript (Piscataway, NJ). The synthesised gene was subsequently cloned into a pET28b vector (Novagen, Darmstadt, Germany) using NdeI and XhoI restriction sites, resulting in both N- and C-terminal hexa-histidine tagged fusion protein. A pET28b vector that carries *radH* halogenase gene from *C*. *chiversii* (UniPort ID: C5H881) was used for this study. The cloning of *radH* into pET28b vector has been previously reported [32, 45].

Similar procedures were used to construct the expression plasmids for the uncharacterised KS/CLF pairs, chosen from *K*. *racemifer* DSM 44953 (KraA/B), *D*. *acidovorans* CCUG 247B (DacA/B), *Streptococcus* sp. GMD2s (SspA/B), Candidatus *D*. *auxilii* (DauA/B), *B*. *endophyticus* DSM 13796 (BendA/B), *P*. *luteoviolacea* DSM 6061 (PluA/B), *Blautia wexlerea* DSM 19850 (BweA/B), *Gloeocapsa* sp. PCC 7428 (GloeA/B), and 2 characterised KS/CLF sequences from *S*. *resistomycificus* (RemA/B) and *Streptomyces antibioticus* ATCC 11891 (OvmP/K) for expression in *E*. *coli*. Nucleotide sequences were refactored to achieve a codon adaptation index similar to highly expressed *E*. *coli* housekeeping genes using a simulated annealing approach. Refactored nucleotide sequences were verified graphically using the %MinMax Rare Codon Calculator [46]. Unfavourable intragenic alternative start sites were identified using the ribosome binding calculator [47] and substituted manually, where necessary. To future proof the use of the KS/CLF gene sequences as biosynthetic parts, NdeI, BamHI, NcoI, PstI, XbaI, EcoRI, HindIII, NotI, and XhoI RE recognition sites were omitted from each gene sequence.

A synthetic duel KS/CLF expression cassette was designed in silico based upon the pETDuet-1 expression vectors (Novagen, Darmstadt, Germany) for *kraA*/*B*, *dauA*/*B*, *bendA*/*B*, *pluA*/*B*, *gloeA*/*B*, *bweA*/*B*, and *remA*/*B*. In brief, the cassette comprised a His^6^-ΔMethionine1_CLF fusion gene sequence, designed using the N-terminal hexahistidine tag (MGSSHHHHHHSQDPNS) nucleotide sequence from pETDuet-1, upstream of the standard pETDuet MCS-2 intergenic region, comprising the T7 promoter for MCS-2, the cognate Shine Dalgarno sequence, and NdeI methionine start codon. In frame with NdeI M1 (MSC2) was the cognate KS gene sequence preceded by an N-terminal Strep-II tag (ASWSHPQFEKG) from pET51b (Novagen, Darmstadt, Germany). The 5′ and 3′ ends of each cassette were flanked by an NcoI and XhoI RE site to facilitate cloning into the expression vector, pETM11-b. All nucleotide sequences were synthesised by GeneArt (ThermoFisher Scientific, Massachusetts, US). Synthetic operons were cloned directly into pETM11-b by GeneArt, forming a series of pETKS/CLF vectors. Gene synthesis *gloeA*/*B* and *bweA*/*B* systematically failed. To construct *dacA*/*B*, *ovmP/K*, and *sspA*/*B* co-expression vectors, each synthetic gene sequence was amplified from holding vectors pHold[KS/CLF] template DNA by PCR using PCR primers containing RE sites within 5′ overhangs. CloneAmp HiFi PCR premix was used for all PCR reactions (Takara, Kusata, Japan) as per the manufacturer’s instructions. All primers’ annealing temperatures (T_a_) were calculated using Integrated DNA Technologies (IDT, Iowa, US) OligoAnalyser 3.1 (https://eu.idtdna.com/calc/analyzer, 2016/17), with T_a_ as close to 50°C as possible. Resultant CLF PCR products were flanked by NdeI and XhoI, and KS PCR products were flanked by NdeI and HindIII and were cloned into MCS-1 and MCS-2 of pETDuet expression vectors forming pETDacB, pETDacAB, pETSspA, pETSspAB, pETOvmP, and pETOvmPK. In all expression vectors, the CLF CDSs were fused with an N-terminal hexahistidine tag, with the exception of Ssp-containing vectors, in which the corresponding KS was His-tagged. For ΔKS expression vector construction, GeneArt-cloned pETKS/CLF expression vectors were linearised by PCR, removing the corresponding KR sequence from the amplicon. PCR primers were designed with complementary 20 bp overhangs to facilitate relegation via Gibson DNA Assembly (NEB) or In Fusion HD cloning (Takara, Kusata, Japan) as per the manufacturer’s instructions. All vectors are detailed in S5 Table.

### Protein purification and peptide identification

Total cell lysate was extracted from *E*. *coli* BL21(DE3) cultures normalised to a total OD_600_ of 4. Normalised cells were centrifuged at 4,000*g*, and supernatant was discarded. To lyse cells, 300 μl of BugBuster (Novagen, Darmstadt, Germany) protein extraction reagent was added to cell pellets and incubated on a rocker for 30 min before centrifugation at 12,000*g* for 20 min, 4°C. Supernatant was removed from cell debris and designated as soluble cell lysate. Cell pellets were resuspended in equal volumes of BugBuster and designated insoluble cell lysate. For protein purification, 400 mL cultures were typically used and cultured as in the “Bacterial strains and culture conditions” section. Once more, culture supernatant was removed by centrifugation, 4,000*g* at 4°C for 20 min. *E*. *coli* BL21(DE3) cell pellets were resuspended in buffer A (50 mM Tris-HCl, 300 mM NaCl [pH 7.4] 5% glycerol [v/v]) supplemented with cOmplete Mini EDTA-free protease inhibitor cocktail (Roche, Basel, Switzerland). All buffers were filtered sterilised using a 0.22 μm syringe filter (Merck). Cell suspension was sonicated on ice for 5 min and centrifuged at 12,000*g* for 25 min, 4°C. Supernatant was removed and centrifuged a second time as above. Supernatant was once more removed and applied to an IMAC column, Ni-NTA agarose (Qiagen, Hilden, Germany), pre-equilibrated with buffer A. Flow through was collected and reapplied to the IMAC column. The column was washed with 5 × column volumes (CV) of buffer A before sequential 1 CV washes with Buffer A comprising increasing concentrations of imidazole. Typically 20, 50, 200, 400, and 500 mM solutions were prepared and are denoted on each SDS-PAGE gel image accordingly. IMAC columns were re-equilibrated in buffer A before washing in 20% ethanol. The pH of all buffers was calculated at 4°C, and all buffer and protein purification was carried out at 4°C.

### Protein purification and peptide identification for AntD/E

His^6^AntE/D and His^6^RemB/A heterodimeric complexes were purified from *E*. *coli* BL21 NiCo21(DE3), using immobilised metal affinity chromatography in 300 mM NaCl, 50 mM tris-HCl (pH 7.4), 50 mM imidazole. His^6^AntE/D was further purified via anion exchange chromatography using a 6 ml resource Q (GE Healthcare Life Sciences, Massachusetts, US) with a linear gradient from 95% to 5% 50 mM Tris-HCl (pH 7.4) against 50 mM Tris-HCl 1M NaCl at 3 ml min^−1^. Samples containing His^6^AntE/D were subsequently separated by size exclusion chromatography using Superdex 200 Increase 100/300 GL columns (GE Healthcare) eluted with 1.5 CVs of 200 mM NaCl, 50 mM tris-HCl (pH 7.4) to isolate the complex in its dimeric form. Backbone vectors containing RFP or GFP were used as protein expression induction controls and to monitor protein extraction efficiency throughout.

To visualise protein samples by SDS-PAGE, protein aliquots were added to fresh 2 × Laemmli SDS-PAGE loading dye (4% SDS [w/v], 0.2% bromophenol blue [w/v], 20% glycerol [v/v], and 200 mM dithiothreitol), made up to 15 μl. Samples were boiled for 10 min prior to loading onto 10%–12% SDS-PAGE gels (Biorad, California, US). Gels were run at 250 V as standard in Towbin buffer (25 mM Tris, 192 mM glycine, 0.1% SDS). PageRule prestained protein ladder (ThermoFisher Scientific, Massachusetts, US) was used as a molecular weight reference, unless stated otherwise. SDS-PAGE gels were stained using InstantBlue protein stain (Expedeon, Cambridge, UK) before washing with water and visualisation using a Gel Doc EZ system (BioRad, California, US). Corresponding western blots followed the above procedure; however, they were not stained with InstantBlue (Expedeon). Instead SDS-PAGE gels were transferred onto nitrocellulose membranes using Trans-Blot Turbo transfer packs (BioRad) as per the manufacturer’s instructions. After transfer, SDS-PAGE gels were stained with InstantBlue (Expedeon) to assess protein transfer quality. Nitrocellulose membranes were washed in deionised water for 5 min before transfer to iBind Western system (ThermoFisher Scientific). Western blots were carried out following the manufacturer’s instructions. Primary H1029_.02ml monoclonal anti-polyhistidine antibodies produced in mouse were purchased from Sigma (Missouri, US). Primary anti-strep-II monoclonal antibodies produced in mouse (71590-100VG) were purchased from Abcam (Cambridge, UK). Secondary antibodies used throughout were ab216772 goat pAb to mouse IgG, IRDye 800CW.

Protein bands of interest were isolated from polyacrylamide gels, and Coomassie stain was removed through alternating dehydration and hydration steps in 50% acetonitrile and 50 mM ammonium bicarbonate before digestion with MS-grade trypsin (Promega, Wisconsin, US) at 37°C for 20 h. Extracts containing tryptic peptides were centrifuged at 13,000 rpm for 10 min to remove particulate matter prior to separation and analysis using a C18 column (LC Packings, Acclaim Pep Map 100) and Bruker (Massachusetts, US) Esquire 3000 Plus ion trap mass spectrometer. Analysis was carried out in positive ion mode with an injection volume 20 μl and flow rate of 200 nl min^−1^ over a gradient of water to 90% acetonitrile both acidified with 0.1% formic acid. Peptide fragments were identified using the Mascot MS/MS ion search software (Matrix Sciences). Mascot MS/MS search results from band 1 (Fig 3, red circle) identified 5 peptide fragments consistent with AntD (FVLGESAFGIPINSLK, LSSGFSGIHSVIVMR, SEDYDSFDFSSAATSVAK, SGAIGQVYGSDGNNKEFVLK, and GAHIYAELAGYASVNNAYHMTDLPADGMAMAR). Similarly, results from band 2 (Fig 3, yellow circle) showed 7 peptide fragments consistent with AntE (LDVDKLDPNR, INEFSNTNQIIIQR, QPGDFSEGAAFLVLEER, IDEFSVYGIVAVEMALK, VVVTGVGAIHPDGNDVTAIK, KIDEFSVYGIVAVEMALK, and RVVVTGVGAIHPDGNDVTAIK). Peptide fragments are identified as [2M+H]^+^ adducts.

### Metabolite extraction and analysis

Both exo- and endo-metabolomes were analysed when elucidating the metabolic profile of *E*. *coli* expressing AntA-I, or substitutions of primary tailoring enzymes. For analysis by HPLC and MS, exometabolomes were analysed rather than endometabolomes. For exometabolome preparation, cells were pelleted at 4,000*g*, 4°C, for 20 min before decanting supernatant and storing on ice. Supernatant was prepared by further centrifugation at 12,000*g*, 4°C, for 20 min to remove cell debris before filtering through a 0.45 μm pore size filter (Merck Millipore, Massachusetts, US) prior to analytics. For endometabolomes, unbiased metabolite extraction of the cell pellet was done as previously described [48]. In brief, cell pellets were flash frozen in liquid nitrogen before being suspended in 80% MeOH (−48°C). Each sample was subsequently flash frozen in liquid nitrogen and allowed to thaw on ice 3 times. Samples were centrifuged at 14,000*g* for 20 min at −9°C before decanting the supernatant. The extraction process was repeated 3 times, and the supernatant from each sample was pooled and used to analyse the exometabolome. Metabolites monitored in each experimental analysis include SEK4 (2), SEK4b (3), B26 (4), AUR367 (5), mutactin (6), dehydromutactin (7), SEK34 (8), SEK34b (9), 1,3,8-trihydroxyanthrone (12), 1,3,8-trihydroxydianthrone (13), aloesaponarin II (11), DMAC (10), AQ256 (1), AQ270a and b, AQ284a and b, and AQ300 and AQ314 [18, 20, 20] (structures not shown). To normalise EICs to final culture optical density, extracted ion intensities were divided by the average optical density (OD_600_) of 3 technical replicates for each sample.

### Octaketide shunt metabolite identification using HPLC-ESI-MS

The exometabolome of *E*. *coli* expressing *antDEF*, *antDEFB*, and *antDEFBG* were analysed by HPLC-ESI-MS (Waters, Massachusetts, US; Acquity Ultra Performance LC, Thermo Scientific, M Massachusetts, US; LTQ Orbitrap XL). HPLC conditions were as follows: 1 min: isocratic gradient of 5% solvent B; 7 min: linear gradient 70% solvent B; 7.5 min: linear gradient 95% solvent B; 8.5 min: isocratic gradient 95% solvent B; 9 min: linear gradient to 5% B; and 10 min: isocratic gradient of 5% B. Solvents A and B were HPLC grade water and HPLC grade acetonitrile both acidified with 0.1% formic acid using a C18 2.6 μm 2.1 × 100 mm LC column (Phenomenex, Macclesfield, UK) heated to 30°C with a flow rate of 0.3 ml min^−1^. Injection volume of 3 μl was analysed by electrospray injection MS in positive ionisation (ES^+^) mode with an ESI-HESI source over a mass scan range of 80–1,200 *m/z*.

### Shunt metabolite identification via HPLC-UV-Vis-ESI-MS

Shunt metabolites (2, 3, 6–9) (S2 Text), dianthones (13) (S2 Text), and modified anthraquinones (structures not shown) (S2 Text) were identified using HPLC-UV-Vis-MS (Agilent, California, US; 1100 series LC/MSD trap). HPLC conditions were as follows: 5 min isocratic gradient of 5% solvent B; 30 min linear gradient from 5 to 95% solvent B; 10 min isocratic gradient of 95% solvent B; 5 min linear gradient from 95–5% solvent B; and 10 min isocratic gradient at 5% B using a C18 2.6 μm 2.1 × 100 mm Accucore LC column (ThermoFisher Scientific) heated to 45°C with a flow rate of 0.25 ml min^−1^. Injection volume of 20 μl was analysed via electrospray injection MS in negative mode.

### Actinorhodin KR and CYC complementation and pACYCAntΔ86 analysis via UV-Vis

Biosynthesis of anthraquinone and BIQ production in actinorhodin KR and CYC complementation experiments was monitored at 434 nm using a Shimadzu prominence UFLC RX SPD-20A UV-Vis detector. Metabolites were separated using a 15 min gradient as follows: 5 min isocratic gradient of 5% B; 15 min linear gradient to 95% B; 5 min isocratic gradient at 95% B; 3 min linear gradient to 5% B; and 7 min isocratic gradient at 5% B. HPLC solvents and column are as in Materials and methods.

### HPLC high-resolution mass spectrum analysis

All experimental samples described here were principally analysed using HPLC high-resolution MS using the Dionex ultimate 3000 rapid separation HPLC coupled with QExactive plus mass spectrometer (Thermo Scientific). HPLC are as follows: 5 min isocratic gradient of 5% solvent B; 15 min linear gradient from 5% to 95% solvent B; 5 min isocratic gradient of 95% solvent B; 3 min linear gradient from 95% to 5% solvent B; and 2 min isocratic gradient at 5% B. Column, column conditions and solvents were as described in Materials and methods but with a flow rate of 0.3 ml min^−1^. The QExactive plus mass spectrometer was operated in both positive and negative ionisation mode using an ESI-HESI source. All mass spectra were recorded using a full mass spectrum scan with data dependent MS-MS (Top5). Full-scan spectra were obtained over a scan range of 80–800 *m/z* with a resolution of 70,000. A resolution of 17,500 was used for routine MS-MS spectra with a default charge state of 1 and collision-induced dissociation energy at 35 eV. Fragmentation patterns of AQ256, aloesaponarin II, emodin, and chrysophanol (S15 Fig) were analysed using HPLC-tSIM-MS-MS with exact masses detailed in an inclusion list. A resolution of 35,000 was used when recording MS-MS spectra. Default charge state and collision dissociation energy were as described above.

### Methodology for mass spectrum data analysis

Mass spectra were recorded in .raw format from all instruments before conversion to .mzML using Proteowizard 3.0.9393 with binary encoding precision of 64-bit, write index, zlib compression, and TPP compatibility selected. Peak picking filters with MS level 1–2 were used as standard. Mass spectra were subsequently analysed using XCMS LC/MS and GC/MS data analysis package [49] using R.

### Methodology for characterisation of AQ256 (1,3,8-trihydroxyanthraquinone)

*E*. *coli* BL21(DE3) harbouring plasmid with the entire anthraquinone cluster, pACYCAnthraquinone, was grown in 6 L of LB medium for large-scale AQ256 production. The cultures were grown at 37°C, 180 rpm, to OD_600_ 0.35–0.4 and induced with 50 μM IPTG. AQ256 was extracted from both cell pellet and culture supernatant using methanol and diethyl ether, respectively. Extracts were evaporated to dryness under vacuum to give a brown oil, which was suspended in 50% methanol before purification by semi-preparative HPLC. The following eluent system was used: 5% B for 0–10 min; 5%–95% B linear gradient for 10–55 min; 95% B for 55–65 min; 95%–5% B for 65–75 min; and 5% B for 75–85 min with a flow rate of 5 mL/min, where solvents A and B were water and acetonitrile acidified with 0.1% formic acid, respectively. The yellow product-containing fractions were combined and evaporated to dryness under reduced pressure before resuspension in 1/10th volume 80% MeOH. Samples were crystallised at 4°C over a period of 48 h; then, the excess solvent was removed. At this point, 15 mg of pure (>95%) AQ256 was obtained, corresponding to a production yield of approximately 2.5 mg/L. The samples were desiccated for 48 h before suspension in deuterated methanol (600 μL, Sigma Aldrich, ≥99.8 atm % D, contains 0.03% [v/v] tetramethylsilane [TMS]) for NMR spectroscopy.

^1^H and COSY NMR spectroscopy was performed using a 400 MHz Bruker NMR spectrometer. ^13^C, HSQC, and HMBC NMR spectroscopy was performed using an 800 MHz Bruker NMR spectrometer (S14 Fig). Compound AQ256 has been reported previously and characterised by ^1^H NMR spectroscopy [50], although no chemical shift assignments were provided. Therefore, assignment of the peaks has been performed here (assignments given below) using analysis of chemical shifts and coupling constants, in combination with COSY, HSQC, and HMBC NMR data. ^1^H NMR (400 MHz, methanol-*d*_4_) δ ppm: 6.60 (d, *J* = 2.5 Hz, 1 H, H-2), 7.23 (d, *J* = 2.5 Hz, 1 H, H-4), 7.30 (dd, *J* = 8.3, 1.0 Hz, 1 H, H-7), 7.71 (t, *J* = 8.1 Hz, 1 H, H-6), and 7.77 (dd, *J* = 7.3, 1.0 Hz, 1 H, H-5). ^13^C NMR (800 MHz, methanol-*d*_4_) δ ppm: 107.6 (C-2), 108.8 (C-4), 109.2 (C-13), 115.4 (C-12), 119.1 (C-5), 124.1 (C-7), 133.6 (C-11), 135.5 (C-14), 136.1 (C-6), 162.0 (C-8), 165.4 (C-1), 166.2 (C-3), 181.7 (C-10), and 190.9 (C-9).

UV-Vis spectroscopy of chrysophanol and emodin standards, as well as AQ256, was performed using a Cary 60 UV-Vis spectrophotometer (Agilent Technologies, California, US), and these are described in S15 Fig.

### Methodology for characterisation of neomedicamycin (1,3-dihydroxy-8-methoxyanthraquinone)

Co-expression of the AntA-I pathway with a previous characterised *O*-methyltransferase (IFMT) from the *M*. *truncatula* isoflavone and isoflavanone pathways produced 1,3-dihydroxy-8-methoxyanthraquinone, a new C8 methoxy-substituted anthraquinone, and represents the first demonstration of this enzyme accepting hydroxyl-substituted anthraquinones. The new compound was named neomedicamycin. *E*. *coli* BL21(DE3) harbouring *antA-I* and *ifmt* was grown in 4.8 L of LB for large-scale neomedicamycin production. The cultures were grown at 37°C, 180 rpm, to OD_600_ 0.35–0.4 and induced with 50 μM IPTG. Neomedicamycin was extracted with 1:1 volume of diethyl ether. The organic phase was visibly yellow and evaporated to dryness under reduced pressure and dissolved in 100% methanol before purification by semi-preparative HPLC using a Phenomenex Gemini 5 μ C18 column (250 × 10 mm) with the following eluent: 10% B 0–2 min; 40% B 2–5 min linear gradient; 40% B 5–50 min isocratic gradient; 95% B 50–51 min linear gradient; 95% B 51–58 min isocratic gradient; 10% B 58–59 min linear gradient; and 10% B 59–65 min isocratic gradient with a flow rate of 5 ml/min. Solvents A and B were water and acetonitrile, respectively, both acidified with 0.05% trifluoroacetic acid. The neomedicamycin-containing fractions were combined and then evaporated to dryness under reduced pressure to give approximately 0.5 mg of purified product, corresponding to a neomedicamycin production yield in *E*. *coli* of 1.04 mg/L in agreement with integrated peak values from crude extracts. This purified fraction was dissolved in deuterated methanol (600 μL, Sigma Aldrich, ≥99.8 atm % D, contains 0.03% [v/v] TMS) for characterisation by NMR spectroscopy.

NMR spectroscopy was performed on a 400 MHz Bruker NMR spectrometer for ^1^H and COSY NMR spectra, whilst a 500 MHz Bruker NMR spectrometer was used to obtain ^13^C, HSQC, and HMBC NMR spectra (S19 Fig). Assignment of the peaks was performed using analysis of chemical shifts and coupling constants, in combination with COSY, HSQC, and HMBC NMR data. Some expected peaks in the ^13^C NMR spectrum were too weak to observe and are therefore not assigned. ^1^H NMR (500 MHz, methanol-*d*_4_), δ ppm: 4.02 (s, 3 H, CH_3_), 6.53 (d, *J* = 2.3 Hz, 1 H, H-2), 7.13 (d, *J* = 2.4 Hz, 1 H, H-4), 7.54 (d, *J* = 8.5 Hz, 1 H, H-7), 7.78 (t, *J* = 8.0 Hz, 1 H, H-6), and 7.89 (d, *J* = 7.6 Hz, 1 H, H-5).

For unambiguous assignment of the structure of neomedicamycin (e.g., the location of the methoxy- group at C8), single crystals suitable for X-ray diffraction analysis were grown by slow evaporation of a saturated solution of diethyl ether at 4°C. Data for neomedicamycin were collected on a dual source Rigaku FR-X rotating anode diffractometer using MoK_α_ wavelength at 150 K and reduced using CrysAlisPro 171.39.30c. Absorption correction was performed using empirical methods (SCALE3 ABSPACK) based upon symmetry-equivalent reflections combined with measurements at different azimuthal angles. The structure was solved and refined against all F^2^ values using Shelx-2016 implemented through Olex2 version 1.2.9 [51, 52]. All crystallographic data are detailed in S3 Table and S19 Fig.

### Methodology for characterisation of neochaetomycin (1,3,8-trihydroxy-monochloroanthraquinone)

Coexpression the *antA-I* pathway with a previous characterised flavin-dependant halogenase *radH* from *C*. *chiversii* yielded a new monochlorinated AQ256 derivative, neochaetomycin, and represents the first demonstration of this enzyme accepting hydroxyl-substituted anthraquinones. *E*. *coli* BL21(DE3) harbouring *antA-I* and *radH* was grown in 0.15 L of M9 for neochaetomycin isolation. Cultures were grown at 37°C, 180 rpm, to OD_600_ 0.35–0.4 and induced with 50 μM IPTG before reducing incubation temperature. Neochaetomycin was extracted using diethyl ether extraction followed by preparative HPLC, using a similar method employed for neomedicamycin but with an extended isocratic gradient of 42% B (as opposed to 40%) to aid peak separation. The neochaetomycin-containing fractions were combined and evaporated to dryness under reduced pressure before dissolution in deuterated methanol (600 μL, Sigma Aldrich, ≥99.8 atm % D, contains 0.03% [v/v] TMS) for characterisation by NMR spectroscopy. Approximately 0.73 mg/L of neochaetomycin were produced by the heterologously expressed pathway in *E*. *coli* prior to large-scale culture optimisation.

A 600 MHz Bruker NMR spectrometer was used to record the ^1^H NMR spectrum. ^1^H NMR (600 MHz, methanol-*d*_4_), δ ppm: 7.10 (s, 1 H, H-2 or H-4), 7.23 (dd, *J* = 7.5, 1.0 Hz, 1 H, H-7), 7.61 (t, *J* = 7.8 Hz with additional splitting unresolved, 1 H, H-6), and 7.70 (dd, *J* = 7.6, 0.9 Hz, 1 H, H-5).

### Phylogenetic analysis of amino acid sequences

Multiple sequence alignments were performed with the Multiple Alignment Fast Fourier Transform (MAFFT) G-INS-1 progressive method [53]. Maximum likelihood phylogenetic trees were generated, bootstrapped with 500 iterations using MEGA6 [54]. Sequence alignment with protein secondary structures in postscript were visualised using ESPript [55].

### Code availability

Scripts used throughout are available upon request.

## Supporting information

S1 Fig KS/CLF phylogeny. Phylogenetic relationship of characterised Actinobacterial and uncharacterised non-Actinobacterial KS/CLFs shown in S1 Table. Type III PKSs (red) and FabH (orange) are used as outgroups, supported by bootstrap values above 95%. Clades a and e represent 55 characterised canonical Actinobacterial CLF and KS amino acid sequences, respectively. Non-Actinobacterial KSs are shown to clade together (Clade d, bootstrap values of >99%), and away from canonical Actinobacterial KSs. Non-Actinobacterial CLF sequences form 2 discrete clades (b and c, unsupported by bootstrapping), which clade apart from canonical Actinobacterial CLFs (bootstrap value: 100%). Maximum likelihood tree was computed as described in S1 Text. Bacterial names are not italicised for clarity purposes.
(TIF)

S2 Fig Multiple sequence alignment of FabF, AntD, AntE, ActIORFI, and ActIORFII. Multiple sequence alignment of FabF, AntD, AntE, ActI ORFI short (act KS), derived from its crystal structure, and ActI ORFII (act CLF) fatty acid synthesis and polyketide synthesis components. The FabF protein secondary structure overlaid is derived from the wild-type *E*. *coli* FabF crystal structure: 2GFW26. The blue arrow shows the catalytic cysteine of FabF, ActIORFI, and AntD, the glutamine in ActI ORFII intrinsic to starter unit decarboxylation and the corresponding aspartic acid in AntE. Black arrows at R207 and L209 show residues important in AcpP:FabF interaction in *E*. *coli* and do not map onto AntE. The red dotted arrow indicates the QIIIQR motif predicted to form β-strand 13 by JPred (doi: 10.1093/nar/gkv332); the red bar indicates the region of nonaligned residues in AntE that form β-strand 13 in FabF, AntD, and both ActI ORFI and ActI ORFII.
(TIF)

S3 Fig Homology model of the AntDE dimer. Homology model of the AntE (grey and red) and AntD (blue) dimer built using SWISS-MODEL (https://swissmodel.expasy.org/) using 1TQY chains B and A, respectively, as target model template. The C-terminal dissimilar third of AntE is coloured red. Homology models were built individually and visualised and dimerised using PyMOL (Schrodinger, New York, US). The red predicted structure of AntE is considerably smaller and more open and disordered than the counterpart structure of the blue KS, AntD. KS, ketosynthase.
(TIF)

S4 Fig RemAB: Soluble heterodimeric recombinant proteins in E. coli. (A) Denaturing PAGE showing insoluble protein extracted from *E*. *coli* BL21(DE3) expressing constituents of the rem mPKS. Lanes 2 and 3: *E*. *coli* BL21(DE3), host background control, without and with 50 μM IPTG. Lanes 4 and 5 *E*. *coli* BL21(DE3) pETRemB without and with 50 μM IPTG induction and lanes 6 and 7: *E*. *coli* BL21(DE3) pETRemAB without and with 50 μM IPTG. Blue circle: His6RemB, green circle: StrepII-RemA. Colours are consistent throughout the figure. (B) Denaturing PAGE of soluble protein as in panel A. (C) Denaturing PAGE gel of RemA/B purified by IMAC. Lane 2: soluble protein extracted from *E*. *coli* BL21(DE3) pETRemAB, lane 3: unbound flow through, lane 4: protein eluting at 20 mM imidazole, lane 5: protein eluting at 50 mM imidazole, lane 6: protein eluting at 200 mM imidazole. Two bands with similar molecular weights to RemA and B can be seen in both lanes 5 and 6, suggesting that RemA copurifies with RemB. (D) Western blot of purified RemAB using anti-polyhistidine primary antibodies, lanes are as described in panel C. A signal corresponding to His^6^RemB is visible in all fractions but is enriched in protein eluting at higher concentrations of imidazole. (E) Western blot of purified RemAB, using anti-streptagII primary antibodies. Lane 1 is soluble protein extracted from *E*. *coli* BL21(DE3) pETRemAB, lane 2: flow through, lane 4 through 6 are protein eluting with 20 mM, 50 mM, and 200 mM imidazole. A signal corresponding to StrepII-RemA is visible in the protein eluted with 50 mM imidazole, consistent with panel C.
(TIF)

S5 Fig Evaluating the solubility of SspA and SspB, KS and CLF, in E. coli. (A) Soluble protein cell lysate of *E*. *coli* BL21(DE3) (NPC) and *E*. *coli* BL21(DE3) harbouring pETDuet-1, pETSspB, and pETSspAB expression vectors induced with 0, 50, or 200 μM IPTG. pETSspA encodes a *His*^*6*^*sspA* fusion gene sequence (His^6^SspA: 46.59 kDa) downstream of a T7 promoter. pETSspAB encodes *His*^*6*^*sspA* and *sspB* (42.86 kDa), both under the control of individual T7 promoters. Gene expression was induced for 16 h at 30°C. His^6^SspA is denoted by a black arrow and visible only in *E*. *coli* BL21(DE3) pETSspAB induced with 50 and 200 μM IPTG. Sample designation and lane number are consistent in panel B, showing insoluble cell lysate. His^6^SspA is visible as an insoluble protein in *E*. *coli* pETSspB and pETSspAB induced with 50 or 200 μM IPTG. No proteins corresponding to the theoretical mass of His^6^SspA are visible in protein extracted from the *E*. *coli* plasmid and background controls. Western blots corroborate these findings (panels C and D), showing recombinant His^6^SspA to soluble only upon co-expression of *sspB*. No signal is detected from soluble protein extracts of *E*. *coli* pETSspA un/induced. His^6^mCherry is positive control (PC: lane 12).
(TIF)

S6 Fig Co-purification of SspA and SspB. SDS-PAGE of polyhistidine containing proteins from *E*. *coli* pETSspAB soluble (a) cell lysate purified by IMAC. His^6^SspA and SspB co-elute after addition of 200 mM and 400 mM imidazole to elution buffer A, denoted by solid and dotted black arrows, respectively. Western blot of soluble (b) purified cell lysate using Mouse anti-polyhistidine IgGa primary antibodies. Signals corresponding to His^6^SspA are present in the soluble protein fraction, FT, and 200 mM and 400 mM imidazole containing eluent. PC:His^6^mCherry is positive control. EF, elusion fraction; FT, flow through; Sol, total soluble cell lysate.
(TIF)

S7 Fig Evaluation of KraAB solubility. Separation and visualisation of soluble (a) and insoluble (b) cell lysate extracted from *E*. *coli* BL21(DE3) expressing *kraA* and *kraB* by SDS-PAGE. *E*. *coli* BL21(DE3) was used as host background control, and *E*. *coli* BL21(DE3) pETM11b and pETM11b-AntB were used as vector controls. Recombinant protein is visible in both soluble and insoluble fractions of *E*. *coli* BL21(DE3) expressing *kraA* and *kraB*; however, the very similar molecular weight of His^6^KraB and StrepII-KraA (47 and 46.8 kDa) makes identification challenging. Both His^6^KraB and StrepII-KraA are resolved independently by western blots using anti-polyhistidine primary antibodies (c) and anti-strep-II primary antibodies (d) (black arrows). His^6^mCherry and StrepII-PluA are used as positive controls, respectively. NPC, no plasmid control.
(TIF)

S8 Fig Evaluation of PluA and B solubility in E. coli. (A) SDS-PAGE analysis of His^6^PluB and StrepII-PluA fusion protein solubility from *E*. *coli* BL21(DE3) pETPluAB total cell lysate. Lanes 1–5 show proteins isolated from *E*. *coli* BL21(DE3) soluble cell lysate, and lanes 7 through 11 show insoluble protein. *E*. *coli* BL21(DE3) and *E*. *coli* BL21(DE3) pETM11b-AntB, induced with 200 μM IPTG, represent host background and vector controls in lanes 1, 2, 7, and 8. Soluble and insoluble protein extracted from *E*. *coli* BL21(DE3) pETPluAB uninduced and induced with 50 μ and 200 μM IPTG are visualised in lanes 3 through 5 and 9 through 11, respectively. StrepII-PluA (46.96 kDa) is visible in both soluble and insoluble protein fractions (panel A), lanes 4 and 5 highlighted by a white dashed arrow and lanes 10 and 11 identified by a black arrow dashed, respectively. His^6^PluB (43.31 kDa) is visible in lanes 10 and 11 (solid black arrow). (B) Western blot counterpart of panel A, resolving strepII-PluA from soluble and insoluble cell lysate protein using Anti-strep-II IgG primary antibodies. (C) Western blot of panel A resolving His^6^PluB from soluble and insoluble cell lysate protein using anti-polyhistidine primary antibodies. Lanes are as described in panel A. Anti-polyhistidine IgG primary antibodies were used. His^6^mCherry positive control is denoted by a red arrow (lane 12). PageRuler prestained protein ladder is used as a molecular weight reference (kDa).
(TIF)

S9 Fig MS-MS spectra of octaketide shunt metabolites, SEK4 and SEK4b, produced by the anthraquinone mPKS. (A) Tandem mass spectrum showing possible fragmentation pattern of SEK4b from *E*. *coli* BL21(DE3) pBbB1a-plumPKS, pACYC8893. Observed mass of each fragment is within a 2.8 ppm tolerance of expected masses. Adducts are as follows: [M-H-C_6_H_6_O_3_]^−^ 191.03 and [M-H-C_10_H_8_O_4_]^−^ 125.02. (B) MS-MS mass spectrum showing possible fragmentation pattern of SEK4 as above, adducts are as follows: [M-H-CO_2_] 273.08, [M-H-CH_2_O_3_]^−^ 255.07 and [M-H-C_3_H_2_O_3_]^−^ 231.07. Mass spectra presented here are representative of 3 biological samples and recorded as described in Materials and methods.
(TIF)

S10 Fig Comparative EICs for octaketide shunt metabolites of interest. A comparison of EICs for metabolites of interest extracted the exometabolome of *E*. *coli* BL21(DE3), *E*. *coli* BL21(DE3) pACYCDuet-1, *E*. *coli* BL21(DE3) pACYCAnthraquinone analysed in both positive and negative ionisation mode [M-H]^−^ (panels A and B) and [M+H]^+^ (panels C and D). EICs show all masses within a ±5 ppm of each metabolites theoretical mass. HPLC-ESI-MS conditions are as described in Materials and methods. Red, blue, and black lines represent EICs of *E*. *coli* BL21 (host control), *E*. *coli* BL21 pACYCDuet-1 (plasmid control), and *E*. *coli* BL21 pACYCAnthraquinone (producing AQ256), all normalised by final cell density (OD_600_). Panels B and D show a zoomed perspective of panels A and C, respectively, enabling identification of minor shunt metabolites. For the purpose of clarity, the EIC displaying masses corresponding to AQ256 are greyed out in panel B. Each EIC is representative of 3 biological replicates. Collectively, EICs show accumulation of AQ256, the predominant metabolite synthesised from the anthraquinone biosynthetic pathway identified using this targeted approach. Additionally, SEK34b also accumulates to high ion intensities.
(TIF)

S11 Fig EICs for expected octaketide shunt metabolites analysed in positive and negative ionisation mode. Typical EICs for expected octaketide shunt metabolites (2, 3, and 6–9) in analysed using negative and positive ionisation mode. EICs a, c, and e compare exometabolomes from the background host *E*. *coli* BL21(DE3), host expressing and empty plasmid, and host expressing *antA-I*, showing EICs from *E*. *coli* BL21(DE3) in red, *E*. *coli* BL21(DE3) pACYCDuet-1 in blue, and *E*. *coli* BL21(DE3) pACYCAnthraquinone in black, respectively. EICs b, d, and f additionally show chromatograms for the KR, ARO/CYC, and Cyc biosynthetic pathway knockouts as *E*. *coli* BL21(DE3) pACYCAntΔAntA, green, *E*. *coli* BL21(DE3) pACYCAntΔAntH, orange, and *E*. *coli* BL21(DE3) pACYCAntΔAntC in sky blue. Ion intensities were normalised by final cell density (OD_600_). Each EIC was limited to the theoretical deprotonated mass ±5 ppm for metabolites of interest. Masses are as follows: for a and b unreduced octaketide SEK4 (2) and SEK4b (3), [M-H]^−^ 317.0651–317.0683 *m/z*; for c and d mutactin (6) and SEK34 (8), [M-H]^−^ 301.0703–301.0733 *m/z*, and for e and f dehydromutactin (7), SEK34b (9) [M-H^−^] 283.0598–283.0626 *m/z*. EICs are representative of 3 biological replicates and were analysed using conditions described in S10 Fig. EIC, extracted ion chromatogram.
(TIF)

S12 Fig HPLC-UV-Vis-ESI-MS analysis of *E. coli* BL21(DE3) expressing AntB-I, ΔAntA. HPLC-UV-Vis-ESI-MS analysis of *E*. *coli* BL21(DE3) expressing *antB-I*, ΔAntA, identifying SEK4 and SEK4b as predominant octaketide shunt metabolites in the ΔKR anthraquinone biosynthetic pathway. (a) Typical chromatogram of culture supernatant from *E*. *coli* BL21(DE3) pACYCAntΔA at monitored 279 nm showing 2 peaks corresponding to SEK4 and SEK4b which are not present in *E*. *coli* BL21(DE3) or *E*. *coli* BL21(DE3) pACYCDuet-1. (b) UV-Vis spectrum for SEK4 (2) with λ_max_ at 231 and 297 nm consistent with previously reported literature [10]. (c) ES^−^ mass spectrum corresponding to SEK4 (2) observed [M-H]^−^ 317.3, theoretical [M-H]^−^ 317.1. (d) UV-Vis spectrum corresponding to SEK4b (3) with λ_max_ of 231 and 297, also consistent with literature [10]. (e) As for panel c; ES^−^ mass spectrum corresponding to SEK4b (3) observed deprotonated mass [M-H]^−^ 317.3, theoretical mass is as for isomeric SEK4. Data presented represent 3 biological replicates and were acquired from *E*. *coli* BL21(DE3) pACYCAntΔAntA samples analysed in S10 Fig. UV-Vis, UV-visibility.
(TIF)

S13 Fig Full mass spectrum and UV-Vis absorption spectrum for two putative dianthrones. Mass spectrum of dianthrones from *antA*-*I* expressing BL21(DE3) at 13.95 min and 14.39 min, respectively, a and b. Deprotonated parent masses for each dianthrone are 2.1 ppm and 2.2 ppm from theoretical mass. The two retention times plausibly correspond to *trans* and *meso* dianthrone isomers. c and d represent MS-MS spectrum for both dianthrones. (e) UV-Vis absorption spectrum for putative dianthrone 1, λ_max_ at 359, 263, 217 nm. (f) ES^−^ MS spectrum corresponding to dianthrone 1 [M-H]^−^ 418.1. (g) UV-Vis spectrum for putative dianthrone 2, λ_max_ at 358, 275, 261, 218 nm. (h) ES^−^ MS spectrum corresponding to dianthrone 1 [M-H]^−^ 480.9. Theoretical mass is [M-H]^−^ 481.0929. Spectra are recorded from the *E*. *coli* BL21(DE3) *antA*-*I* exometabolome. Each figure is a consensus of 3 biological samples. UV-Vis, UV-visibility.
(TIF)

S14 Fig NMR spectroscopy of AQ256. (a) Complete ^1^H NMR spectrum (400 MHz, methanol-*d*_4_, 298 K) in deuterated methanol with TMS and methanol peaks annotated. (b) Expanded ^1^H NMR spectrum showing aromatic proton signals. (c) ^13^C NMR spectrum (800 MHz, methanol-*d*_4_, 298 K) with TMS standard: the methanol solvent peak is annotated. (d) HSQC NMR spectrum (800 MHz, methanol-*d*_4_, 298 K). (e) Expansion of the HSQC spectrum showing relationship between aromatic protons and the corresponding carbon atoms. (f) A two-dimensional ^1^H-^1^H COSY NMR spectrum (800 MHz, methanol-*d*_4_, 298 K) showing proton–proton coupling. (g) HMBC NMR spectrum (800 MHz, methanol-*d*_4_, 298 K) with TMS standard. (h) Expansion of the HMBC NMR spectrum showing relationship between the aromatic protons and the corresponding carbon atoms.
(TIF)

S15 Fig Evaluation of chrysophanol, emodin, aloesaponarin II, and AQ256 MS-MS spectra and UV-Vis spectra. (a–d) Evaluation of chrysophanol, emodin, aloesaponarin II, and AQ256 MS-MS spectra (ES^−^). The measured MS-MS spectra values are depicted under each structure. (e) UV-Vis absorbance spectra for AQ256 (100% methanol), and 2 other anthraquinones, emodin and chrysophanol (80% methanol), recorded between 250 and 700 nm using a Cary 60 UV-Vis spectrophotometer (Agilent Technologies). All samples show common λ_max_ at approximately 430 nm. UV-Vis, UV-visible.
(TIF)

S16 Fig EIC of KR complemented anthraquinone biosynthetic pathways. A comparison of EICs from the exometabolome of *E*. *coli* BL21(DE3) expressing the anthraquinone pathway complemented with ActIII, a KR from the actinorhodin biosynthetic pathway. All samples are numbered as media blank—1: *E*. *coli* BL21(DE3) wild type, 2: *E*. *coli* BL21(DE3) pACYCDuet-1, 3: *E*. *coli* BL21(DE3) pACYCAntΔAntA, 4: *E*. *coli* BL21(DE3) pACYCAnt**ref**KR (refactored sequence), 5: *E*. *coli* BL21(DE3) pACYCAnt**wt**KR (modified wild-type sequence), 6: *E*. *coli* BL21(DE3) pACYCAnthraquinone, and 7: Ion intensities were normalised to final cell density (OD_600_). Panels a and b show two-dimensional and three-dimensional EICs of all observable masses between 255.0286 and 255.0312 *m/z*: AQ256 theoretical mass of [M-H]^−^ 255.02989. Panels c and d show EIC of masses between 317.0651 and 317.0683 m/z: SEK4 and SEK4b theoretical mass of [M-H]^−^ 317.0667. All EICs use a ±5 ppm cutoff for identification of metabolite of interest and are representative of 3 biological samples, and HPLC conditions are as described in Materials and methods. AQ256 biosynthesis is observed in *E*. *coli* BL21(DE3) expressing *antA-I*, and restored in *E*. *coli* BL21(DE3) expressing *AntB-I* complemented with the actinorhodin KR but not in the ΔAntA host. Instead, the exometabolome of *E*. *coli* BL21(DE3) pACYCAntΔAntA is enriched in SEK4 and SEK4b, indicating a metabolic bottleneck to occur before ketoreduction of C9, as expected. Bolded numbers represent metabolites detailed in Fig 2. EIC, extracted ion chromatogram; KR, ketoreductase.
(TIF)

S17 Fig HPLC-UV-Vis-MS analysis of *E. coli* BL21(DE3) expressing AntA-G, I, ΔAntH. HPLC-UV-Vis-MS analysis of *E*. *coli* BL21(DE3) expressing *antA-G*, *I*, ΔAntH, identifying mutactin as predominant octaketide shunt metabolites in the ΔAntH anthraquinone biosynthetic pathway. All chromatographic conditions and methods are as described in Materials and methods. (A) Typical chromatogram of culture supernatant from *E*. *coli* BL21(DE3) pACYCAntΔH at 269 nm showing a peak corresponding mutactin which is not present in *E*. *coli* BL21(DE3) or *E*. *coli* BL21(DE3) pACYCDuet-1. (B) UV-Vis spectrum for mutactin (6) with λ_max_ at 223 and 269 nm consistent with previously reported literature [1]. (C) ES^−^ mass spectrum corresponding to mutactin (6) observed [M-H]^−^ 301.2, theoretical [M-H]^−^ 301.1. Data presented here are representative of 3 biological replicates and were acquired from *E*. *coli* pACYCAntΔAntH samples analysed in S10 Fig [56]. UV-Vis, UV-visible.
(TIF)

S18 Fig UV-Vis spectrum and EIC of neomedicamycin (1,3-dihydroxy-8-methoxyanthraquinone). (A) UV-Vis absorbance spectrum for neomedicamycin in water:acetonitrile (0.05% TFA). λ_max_: 213, 245, 282, and 428 nm, λ_min_: 237, 256, and 317 nm. (B) EIC of neomedicamycin. Retention times for neomedicamycin t_R_: 426, AQ256 t_R_: 478. Exact masses neomedicamycin [M-H^−^]: 296.04568, 0.5 ppm from theoretical mass of 269.04555; AQ256 [M-H^−^]: 255.02983, 0.27 ppm from theoretical deprotonated mass of 255.02989. The red and black lines show EICs of 296.04568 ± 5 ppm and 255.02989 ± 5 ppm, respectively. Masses corresponding to methoxy-substituted AQ256 were not detectable in *E*. *coli* BL21(DE3) expressing *antA-I*. EIC, extracted ion chromatogram; UV-Vis, UV-visible.
(TIF)

S19 Fig Characterisation of neomedicamycin (1,3-dihydroxy-8-methoxyanthraquinone). (A) ^1^H NMR spectrum (500 MHz, methanol-*d*_4_, 298 K). Values for integrated peak areas are denoted in red: TMS present at 0.03% vol/vol. Inset shows the expanded aromatic region. (B) COSY NMR spectrum (500 MHz, methanol-*d*_4_, 298 K). (C) HSQC NMR spectrum (500 MHz, methanol-*d*_4_, 298 K). (C) HMBC NMR spectrum (500 MHz, methanol-*d*_4_, 298 K). (D) X-ray crystal structure of neomedicamycin. Carbon = grey, oxygen = red, and hydrogen = white; image produced using POV-ray. TMS, Tetramethylsilane.
(TIF)

S20 Fig Characterisation of neochaetomycin (1,3,8-trihydroxy-monochloroanthraquinone). (A) UV-Vis absorbance spectrum for neochaetomycin in water:acetonitrile (0.05% TFA). λ_max_: 213, 243, 276, and 432 nm, λ_min_: 238, 253, 335, and 353 nm. (B) Chromatograms 1 and 2 represent EICs of all masses between 288.99092 ± 5 ppm from *E*. *coli* BL21(DE3) expressing *antA-I* and *E*. *coli* BL21(DE3) expressing *antA-I* and *radH*. A mass corresponding to neochaetomycin is detectable only in *E*. *coli* BL21(DE3) expressing the *antA-I* pathway as well as *radH* ([M-H]^−^ 288.99066, 0.9 ppm for the theoretical mass of [M-H]^−^ 288.99092). Chromatograms 3 and 4 represent EICs of all masses between 255.02989 ± 5 ppm from *E*. *coli* BL21(DE3) expressing *antA-I* and *E*. *coli* BL21(DE3) expressing *antA-I* and *radH*. AQ256 can be detected in both at similar intensities. EIC ion intensities were normalised to final cell densities to enable comparison. Comparison of (C) the ^1^H NMR spectrum for AQ256 (400 MHz, methanol-*d*_4_, 298 K) to (D) the ^1^H NMR spectra of neochaetomycin (600 MHz, methanol-*d*_4_, 298 K). This comparison suggests that chlorination occurs at position 2, as the resonance at 6.60 ppm assigned to this proton in AQ256 disappears. However, this tentative assignment cannot be confirmed until further characterisation data are obtained. UV-Vis, UV-visible.
(TIF)

S21 Fig HPLC-UV-Vis-MS analysis of *E. coli* BL21(DE3) expressing AntA, B, D-I, ΔAntC. HPLC-UV-Vis-MS analysis of *E*. *coli* BL21(DE3) expressing *antA*, *B*, *D-I*, ΔAntC, identifying SEK34 as predominant octaketide shunt metabolites in the ΔAntC anthraquinone biosynthetic pathway. Chromatographic conditions and methods are as described in Supplementary Figure 38. (A) Typical chromatogram of culture supernatant from *E*. *coli* BL21(DE3) pACYCAntΔC at 258 nm showing a peak corresponding SEK34 which is not present in *E*. *coli* BL21(DE3) or *E*. *coli* BL21(DE3) pACYCDuet-1. (B) UV-Vis spectrum for SEK34 (8) with λ_max_ at 258 and 288 nm consistent with previously reported literature [1]. (C) ES^−^ mass spectrum corresponding to SEK34 (8) observed [M-H]^−^ 301.1, theoretical [M-H]^−^ 301.1, presence of an abundant mass at 257.3 *m/z* is also previously reported for SEK34. Data presented are in strong agreement with 2 additional biological replicates and were acquired from *E*. *coli* BL21(DE3) pACYCAntΔAntC samples analysed in S10 Fig [56].
(TIF)

S22 Fig HPLC-UV-Vis-MS analysis of *E. coli* BL21(DE3) expressing AntA-I and AQ256 standards. HPLC-UV-Vis-MS analysis of *E*. *coli* BL21(DE3) expressing *antA-I* identifying AQ256 as the predominant end compound of the anthraquinone biosynthetic gene cluster. Data are representative of the same 3 biological replicates. All chromatographic conditions and methods are as described in Materials and methods. (A) Typical chromatogram of culture supernatant from *E*. *coli* BL21(DE3) pACYCAnthraquinone monitored at 434 nm showing a peak corresponding AQ256 which is not present in *E*. *coli* BL21(DE3) or *E*. *coli* BL21(DE3) pACYCDuet-1. Additionally, no other major peaks are present at this wavelength indicating this to be the only anthraquinone produced. (B) Typical chromatogram of AQ256 analytical standard purified from *E*. *coli* BL21(DE3) pACYCAnthraquinone and characterised by ^1^H, ^13^C, COSY, HSQC, and HMBC NMR spectroscopy. (C) UV-Vis spectrum for AQ256 with λ_max_ at 244, 265, 284, and 434 nm consistent with the counterpart UV-Vis spectrum of the AQ256 standard in D. (E and F) ES^−^ mass spectrum corresponding to AQ256 derived from *E*. *coli* BL21(DE3) pACYCAnthraquinone and AQ256 analytical standard, respectively. Observed masses are [M-H]^−^ 254.9 and [M-H]^−^ 254.8, theoretical mass [M-H]^−^ 255. UV-Vis and mass spectra are in good agreement between experimental samples and analytical standards. UV-Vis, UV-visible.
(TIF)

S1 Table Non-Actinobacterial organisms comprising one or more predicted type II PKS BGCs. Underlined organisms contain characterised BGCs. Coloured fields show organisms comprising BGCs predicted to produce the same or extremely similar specialised metabolites. Type II PKS BGCs from the underlined organisms are selected for this study. *K. *racemifer* comprises 3 predicted type II PKS BGCs: two satisfy manual curation criteria.
(DOCX)

S2 Table CLF gatekeeper residues for biosynthesis of different length nascent poly-β-ketide chains. A table displaying the gatekeeper residues from a series of CLFs with bulky R-groups which sterically reduce the size of the amphipathic tunnel at the KS/CLF dimer interface [1]. Residue order represents their proximity to the cavity entrance. Red residues define the bottom of the cavity, while blue AAs are smaller residues from homologues producing longer polyketides. Gatekeeper residues do not map to the anthraquinone sequence; prediction of chain length using this method suggests the nascent poly-β-ketide to be C_20_ [57].
(DOCX)

S3 Table Theoretical masses for all shunt metabolites. All theoretical masses used in this study are listed. Isomers are highlighted in corresponding colours. All masses are reported as atomic mass units.
(DOCX)

S4 Table Primers used for plasmid construction. Primer nomenclature is typically gene/region amplified_direction of amplification_restriction endonuclease site. All primers with additional 5′ RE sequences are preceded with an additional random 6 bp sequence to facilitate PCR product digestion. RE recognition sequences are bolded. Primers were designed and verified with IDT oligoanalyser. RE, restriction endonuclease.
(DOCX)

S5 Table Plasmids used in this study. All plasmids used and constricted in this study are shown. *Sequence optimised using GeneArt GeneOptimiser (ThermoFischer Scientific, Massachusetts, US) and synthesised by Gen9 (Massachusetts, US).
(DOCX)

S1 Text Bioinformatics analysis. Detailed bioinformatic analysis of the actinobacterial KS and CLF.
(DOCX)

S2 Text Elucidation of the major metabolites produced by the anthraquinone biosynthetic gene cluster. (DOCX)

S3 Text Elucidation of Neomedicamycin (1,3-dihydroxy-8-methoxyanthraquinone) and neochaetomycin (1,3,8-trihydroxy-monochloroanthraquinone). (DOCX)

## Abbreviations

ACP: acyl carrier protein
ACL: ATP citrate lyase
BIQ: benzoisochromanequinone
BGC: biosynthesis gene cluster
CLF: chain length factor
CoA: Coenzyme A
COSY: correlation spectroscopy
CV: column volume
DMAC: 3,8-dihydroxy-methylanthraquinone carboxylic acid
EIC: extracted ion chromatogram
gDNA: genomic DNA
His^6^: 6 polyhistidine tag
HMBC: heteronuclear multiple bond correlation
HSQC: heteronuclear single quantum correlation
IMAC: immobilised metal ion affinity chromatography
IPTG: isopropyl β-D-1-thiogalactopyranoside
KR: ketoreductase; KS, ketosynthase
LB: lysogeny broth
LC/MS-MS: liquid chromatography tandem mass spectrometry
MAFFT: Multiple Alignment Fast Fourier Transform
MCAT: malonyl-CoA ACP transacylase
MCS: multiple cloning site
MIBiG: Minimum Information about a Biosynthetic Gene cluster
mPKS: minimal polyketide synthase
NEB: New England Biolabs
NMR: nuclear magnetic resonance
NR-PKS: nonreducing fungal PKS
OD_600_: optical density measured at 600 nm
OTC: oxytetracycline
PKS: polyketide synthase
PPTase: phosphopantetheinyl transferase
RE: restriction endonuclease
SDS-PAGE: sodium dodecyl sulfate–polyacrylamide gel electrophoresis
Strep-II tag: Streptavidin-II tag
TMS: tetramethylsilane
UV-Vis: UV-visible
