# Supplementary figures and images for: Assembling a plug-and-play production line for combinatorial biosynthesis of aromatic polyketides in Escherichia coli
Source: PLoS Biol. 2019 Jul 18;17(7):e3000347. doi: 10.1371/journal.pbio.3000347 (PMC6638757; doi:10.1371/journal.pbio.3000347)

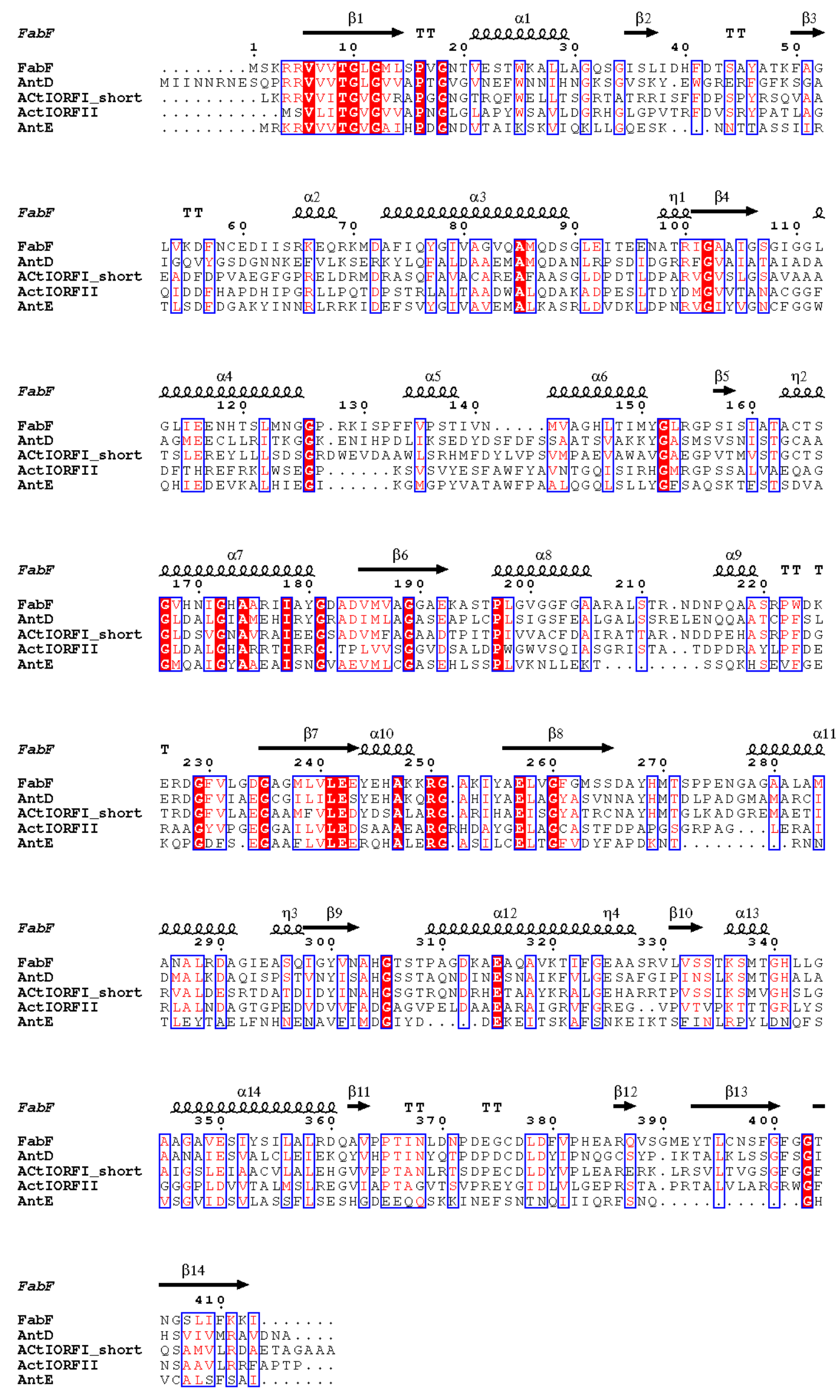

Supplement: S2 Fig — Multiple sequence alignment of FabF, AntD, AntE, ActI ORFI short (act KS), derived from its crystal structure, and ActI ORFII (act CLF) fatty acid synthesis and polyketide synthesis components. The FabF protein secondary structure overlaid is derived from the wild-type E. coli FabF crystal structure: 2GFW26. The blue arrow shows the catalytic cysteine of FabF, ActIORFI, and AntD, the glutamine in ActI ORFII intrinsic to starter unit decarboxylation and the corresponding aspartic acid in AntE. Black arrows at R207 and L209 show residues important in AcpP:FabF interaction in E. coli and do not map onto AntE. The red dotted arrow indicates the QIIIQR motif predicted to form β-strand 13 by JPred (doi: 10.1093/nar/gkv332); the red bar indicates the region of nonaligned residues in AntE that form β-strand 13 in FabF, AntD, and both ActI ORFI and ActI ORFII. (TIF) [file pbio.3000347.s002.tif]

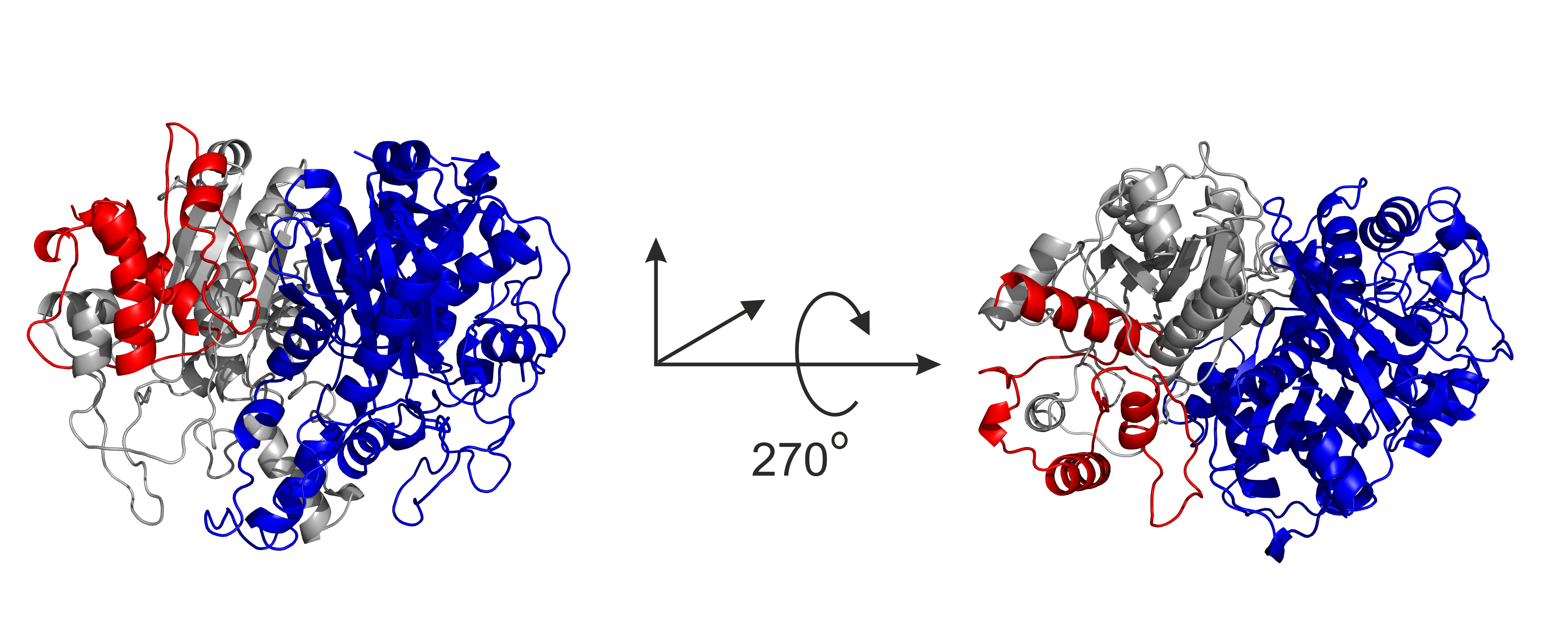

Supplement: S3 Fig — Homology model of the AntE (grey and red) and AntD (blue) dimer built using SWISS-MODEL (https://swissmodel.expasy.org/) using 1TQY chains B and A, respectively, as target model template. The C-terminal dissimilar third of AntE is coloured red. Homology models were built individually and visualised and dimerised using PyMOL (Schrodinger, New York, US). The red predicted structure of AntE is considerably smaller and more open and disordered than the counterpart structure of the blue KS, AntD. KS, ketosynthase. (TIF) [file pbio.3000347.s003.tif]

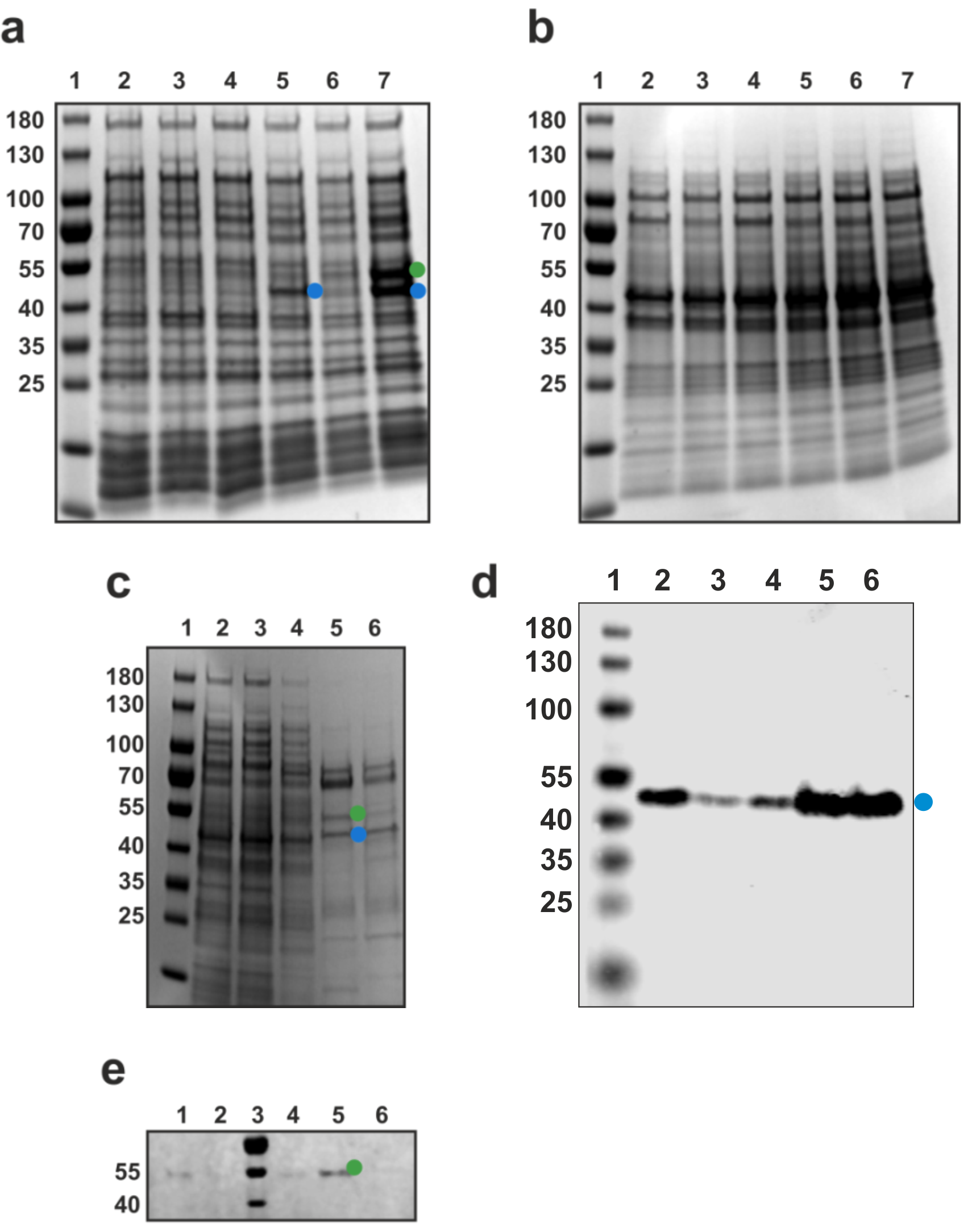

Supplement: S4 Fig — (A) Denaturing PAGE showing insoluble protein extracted from E. coli BL21(DE3) expressing constituents of the rem mPKS. Lanes 2 and 3: E. coli BL21(DE3), host background control, without and with 50 μM IPTG. Lanes 4 and 5 E. coli BL21(DE3) pETRemB without and with 50 μM IPTG induction and lanes 6 and 7: E. coli BL21(DE3) pETRemAB without and with 50 μM IPTG. Blue circle: His6RemB, green circle: StrepII-RemA. Colours are consistent throughout the figure. (B) Denaturing PAGE of soluble protein as in panel A. (C) Denaturing PAGE gel of RemA/B purified by IMAC. Lane 2: soluble protein extracted from E. coli BL21(DE3) pETRemAB, lane 3: unbound flow through, lane 4: protein eluting at 20 mM imidazole, lane 5: protein eluting at 50 mM imidazole, lane 6: protein eluting at 200 mM imidazole. Two bands with similar molecular weights to RemA and B can be seen in both lanes 5 and 6, suggesting that RemA copurifies with RemB. (D) Western blot of purified RemAB using anti-polyhistidine primary antibodies, lanes are as described in panel C. A signal corresponding to His6RemB is visible in all fractions but is enriched in protein eluting at higher concentrations of imidazole. (E) Western blot of purified RemAB, using anti-streptagII primary antibodies. Lane 1 is soluble protein extracted from E. coli BL21(DE3) pETRemAB, lane 2: flow through, lane 4 through 6 are protein eluting with 20 mM, 50 mM, and 200 mM imidazole. A signal corresponding to StrepII-RemA is visible in the protein eluted with 50 mM imidazole, consistent with panel C. (TIF) [file pbio.3000347.s004.tif]

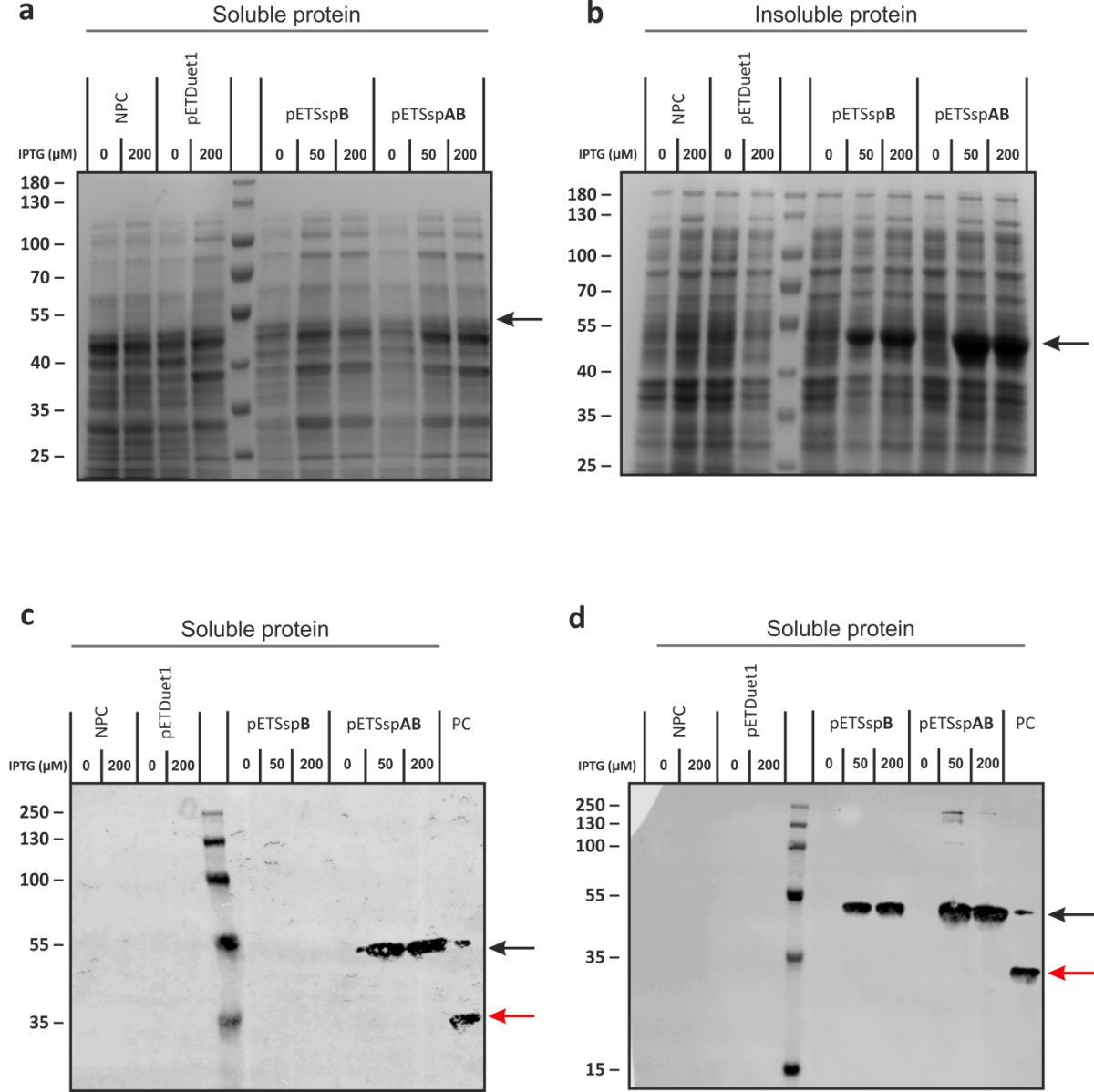

Supplement: S5 Fig — (A) Soluble protein cell lysate of E. coli BL21(DE3) (NPC) and E. coli BL21(DE3) harbouring pETDuet-1, pETSspB, and pETSspAB expression vectors induced with 0, 50, or 200 μM IPTG. pETSspA encodes a His6sspA fusion gene sequence (His6SspA: 46.59 kDa) downstream of a T7 promoter. pETSspAB encodes His6sspA and sspB (42.86 kDa), both under the control of individual T7 promoters. Gene expression was induced for 16 h at 30°C. His6SspA is denoted by a black arrow and visible only in E. coli BL21(DE3) pETSspAB induced with 50 and 200 μM IPTG. Sample designation and lane number are consistent in panel B, showing insoluble cell lysate. His6SspA is visible as an insoluble protein in E. coli pETSspB and pETSspAB induced with 50 or 200 μM IPTG. No proteins corresponding to the theoretical mass of His6SspA are visible in protein extracted from the E. coli plasmid and background controls. Western blots corroborate these findings (panels C and D), showing recombinant His6SspA to soluble only upon co-expression of sspB. No signal is detected from soluble protein extracts of E. coli pETSspA un/induced. His6mCherry is positive control (PC: lane 12). (TIF) [file pbio.3000347.s005.tif]

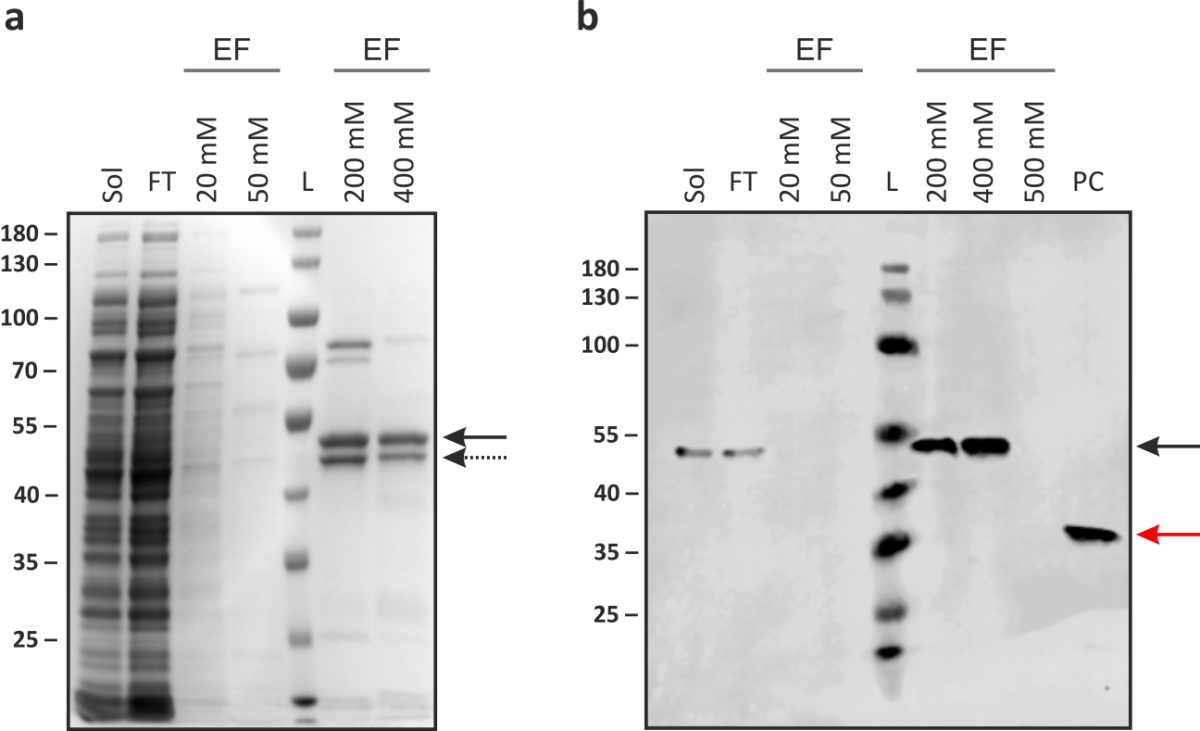

Supplement: S6 Fig — SDS-PAGE of polyhistidine containing proteins from E. coli pETSspAB soluble (a) cell lysate purified by IMAC. His6SspA and SspB co-elute after addition of 200 mM and 400 mM imidazole to elution buffer A, denoted by solid and dotted black arrows, respectively. Western blot of soluble (b) purified cell lysate using Mouse anti-polyhistidine IgGa primary antibodies. Signals corresponding to His6SspA are present in the soluble protein fraction, FT, and 200 mM and 400 mM imidazole containing eluent. PC:His6mCherry is positive control. EF, elusion fraction; FT, flow through; Sol, total soluble cell lysate. (TIF) [file pbio.3000347.s006.tif]

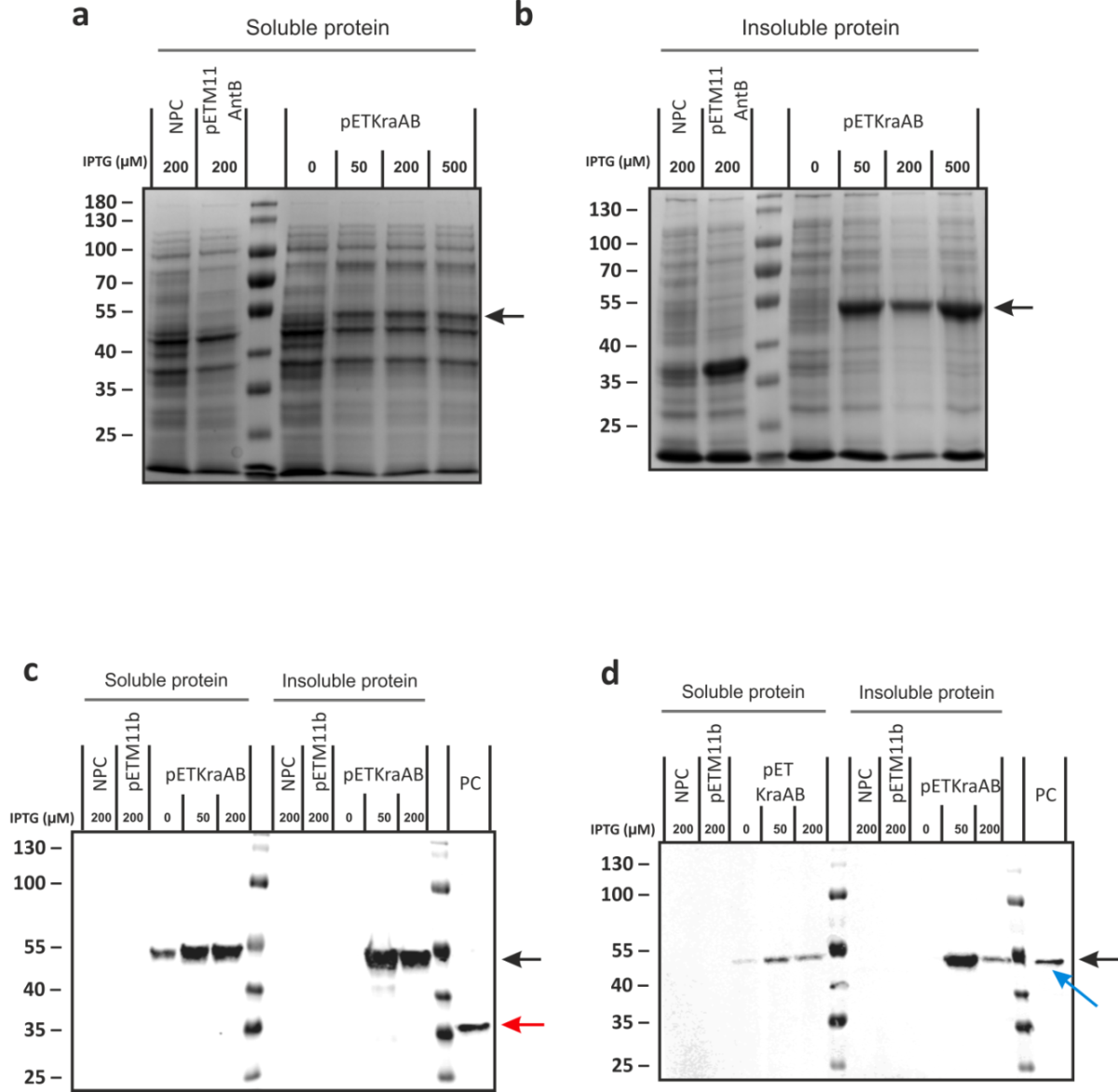

Supplement: S7 Fig — Separation and visualisation of soluble (a) and insoluble (b) cell lysate extracted from E. coli BL21(DE3) expressing kraA and kraB by SDS-PAGE. E. coli BL21(DE3) was used as host background control, and E. coli BL21(DE3) pETM11b and pETM11b-AntB were used as vector controls. Recombinant protein is visible in both soluble and insoluble fractions of E. coli BL21(DE3) expressing kraA and kraB; however, the very similar molecular weight of His6KraB and StrepII-KraA (47 and 46.8 kDa) makes identification challenging. Both His6KraB and StrepII-KraA are resolved independently by western blots using anti-polyhistidine primary antibodies (c) and anti-strep-II primary antibodies (d) (black arrows). His6mCherry and StrepII-PluA are used as positive controls, respectively. NPC, no plasmid control. (TIF) [file pbio.3000347.s007.tif]

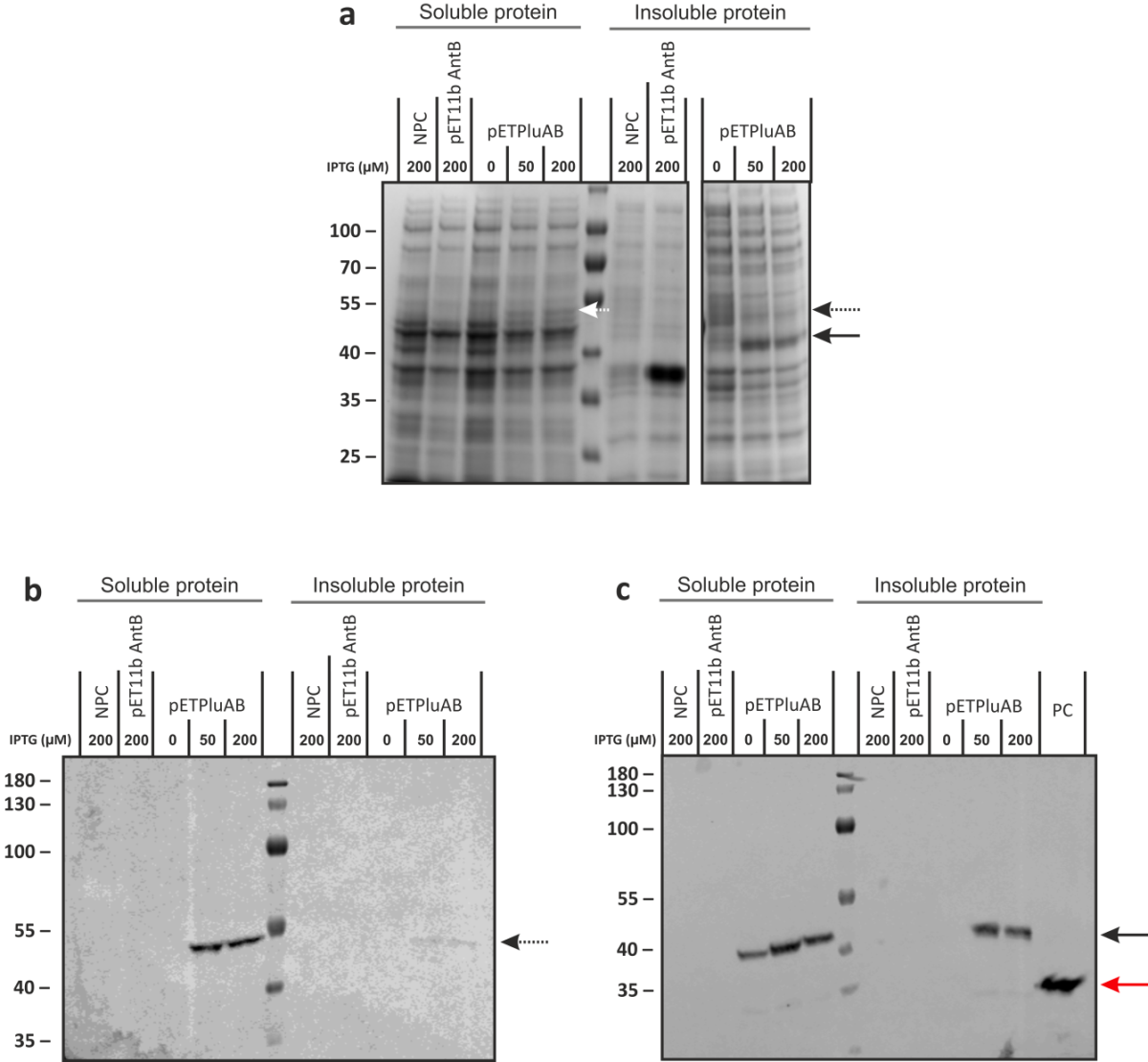

Supplement: S8 Fig — (A) SDS-PAGE analysis of His6PluB and StrepII-PluA fusion protein solubility from E. coli BL21(DE3) pETPluAB total cell lysate. Lanes 1–5 show proteins isolated from E. coli BL21(DE3) soluble cell lysate, and lanes 7 through 11 show insoluble protein. E. coli BL21(DE3) and E. coli BL21(DE3) pETM11b-AntB, induced with 200 μM IPTG, represent host background and vector controls in lanes 1, 2, 7, and 8. Soluble and insoluble protein extracted from E. coli BL21(DE3) pETPluAB uninduced and induced with 50 μ and 200 μM IPTG are visualised in lanes 3 through 5 and 9 through 11, respectively. StrepII-PluA (46.96 kDa) is visible in both soluble and insoluble protein fractions (panel A), lanes 4 and 5 highlighted by a white dashed arrow and lanes 10 and 11 identified by a black arrow dashed, respectively. His6PluB (43.31 kDa) is visible in lanes 10 and 11 (solid black arrow). (B) Western blot counterpart of panel A, resolving strepII-PluA from soluble and insoluble cell lysate protein using Anti-strep-II IgG primary antibodies. (C) Western blot of panel A resolving His6PluB from soluble and insoluble cell lysate protein using anti-polyhistidine primary antibodies. Lanes are as described in panel A. Anti-polyhistidine IgG primary antibodies were used. His6mCherry positive control is denoted by a red arrow (lane 12). PageRuler prestained protein ladder is used as a molecular weight reference (kDa). (TIF) [file pbio.3000347.s008.tif]

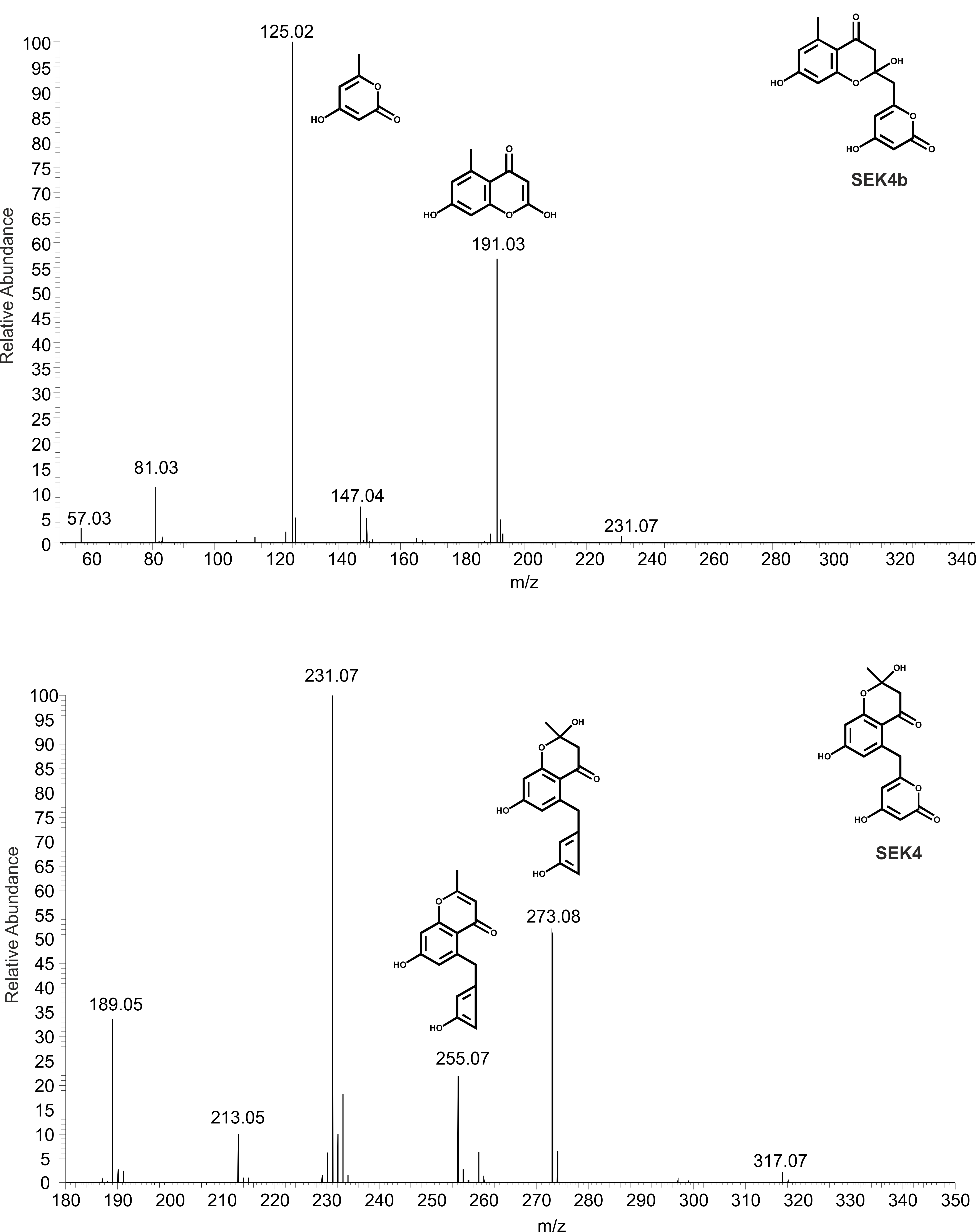

Supplement: S9 Fig — (A) Tandem mass spectrum showing possible fragmentation pattern of SEK4b from E. coli BL21(DE3) pBbB1a-plumPKS, pACYC8893. Observed mass of each fragment is within a 2.8 ppm tolerance of expected masses. Adducts are as follows: [M-H-C6H6O3]− 191.03 and [M-H-C10H8O4]− 125.02. (B) MS-MS mass spectrum showing possible fragmentation pattern of SEK4 as above, adducts are as follows: [M-H-CO2] 273.08, [M-H-CH2O3]− 255.07 and [M-H-C3H2O3]− 231.07. Mass spectra presented here are representative of 3 biological samples and recorded as described in Materials and methods. (TIF) [file pbio.3000347.s009.tif]

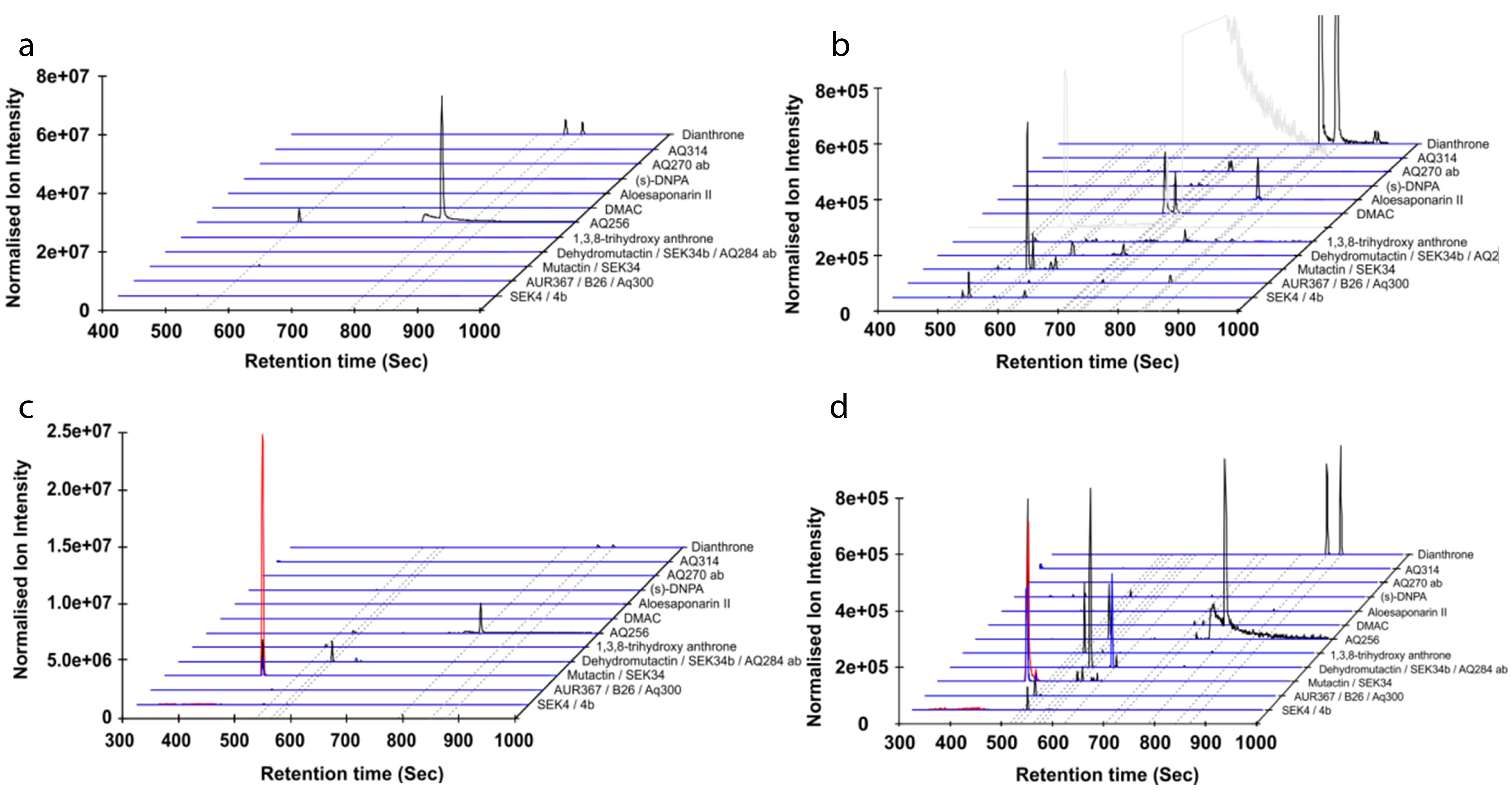

Supplement: S10 Fig — A comparison of EICs for metabolites of interest extracted the exometabolome of E. coli BL21(DE3), E. coli BL21(DE3) pACYCDuet-1, E. coli BL21(DE3) pACYCAnthraquinone analysed in both positive and negative ionisation mode [M-H]− (panels A and B) and [M+H]+ (panels C and D). EICs show all masses within a ±5 ppm of each metabolites theoretical mass. HPLC-ESI-MS conditions are as described in Materials and methods. Red, blue, and black lines represent EICs of E. coli BL21 (host control), E. coli BL21 pACYCDuet-1 (plasmid control), and E. coli BL21 pACYCAnthraquinone (producing AQ256), all normalised by final cell density (OD600). Panels B and D show a zoomed perspective of panels A and C, respectively, enabling identification of minor shunt metabolites. For the purpose of clarity, the EIC displaying masses corresponding to AQ256 are greyed out in panel B. Each EIC is representative of 3 biological replicates. Collectively, EICs show accumulation of AQ256, the predominant metabolite synthesised from the anthraquinone biosynthetic pathway identified using this targeted approach. Additionally, SEK34b also accumulates to high ion intensities. (TIF) [file pbio.3000347.s010.tif]

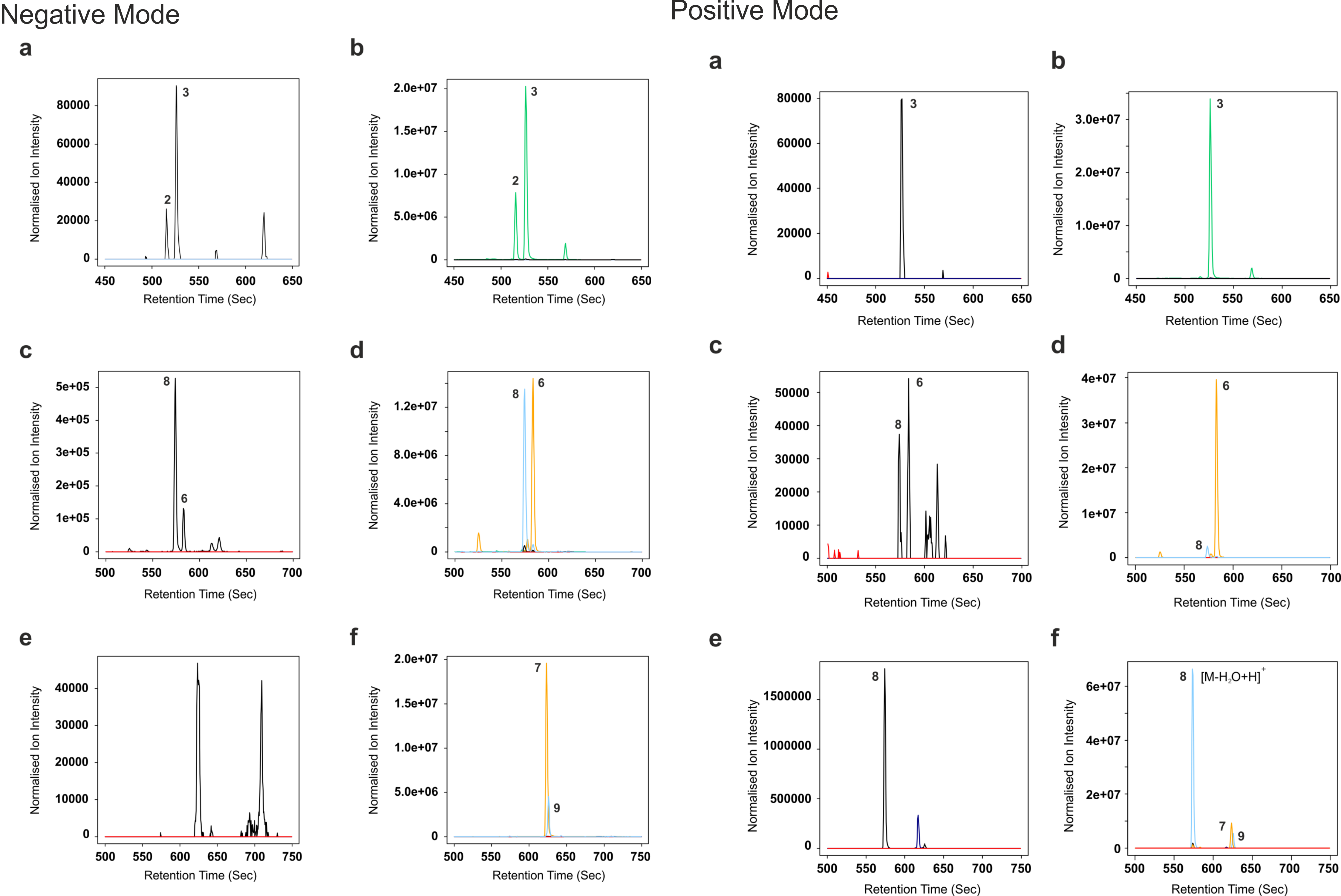

Supplement: S11 Fig — Typical EICs for expected octaketide shunt metabolites (2, 3, and 6–9) in analysed using negative and positive ionisation mode. EICs a, c, and e compare exometabolomes from the background host E. coli BL21(DE3), host expressing and empty plasmid, and host expressing antA-I, showing EICs from E. coli BL21(DE3) in red, E. coli BL21(DE3) pACYCDuet-1 in blue, and E. coli BL21(DE3) pACYCAnthraquinone in black, respectively. EICs b, d, and f additionally show chromatograms for the KR, ARO/CYC, and Cyc biosynthetic pathway knockouts as E. coli BL21(DE3) pACYCAntΔAntA, green, E. coli BL21(DE3) pACYCAntΔAntH, orange, and E. coli BL21(DE3) pACYCAntΔAntC in sky blue. Ion intensities were normalised by final cell density (OD600). Each EIC was limited to the theoretical deprotonated mass ±5 ppm for metabolites of interest. Masses are as follows: for a and b unreduced octaketide SEK4 (2) and SEK4b (3), [M-H]− 317.0651–317.0683 m/z; for c and d mutactin (6) and SEK34 (8), [M-H]− 301.0703–301.0733 m/z, and for e and f dehydromutactin (7), SEK34b (9) [M-H−] 283.0598–283.0626 m/z. EICs are representative of 3 biological replicates and were analysed using conditions described in S10 Fig. EIC, extracted ion chromatogram. (TIF) [file pbio.3000347.s011.tif]

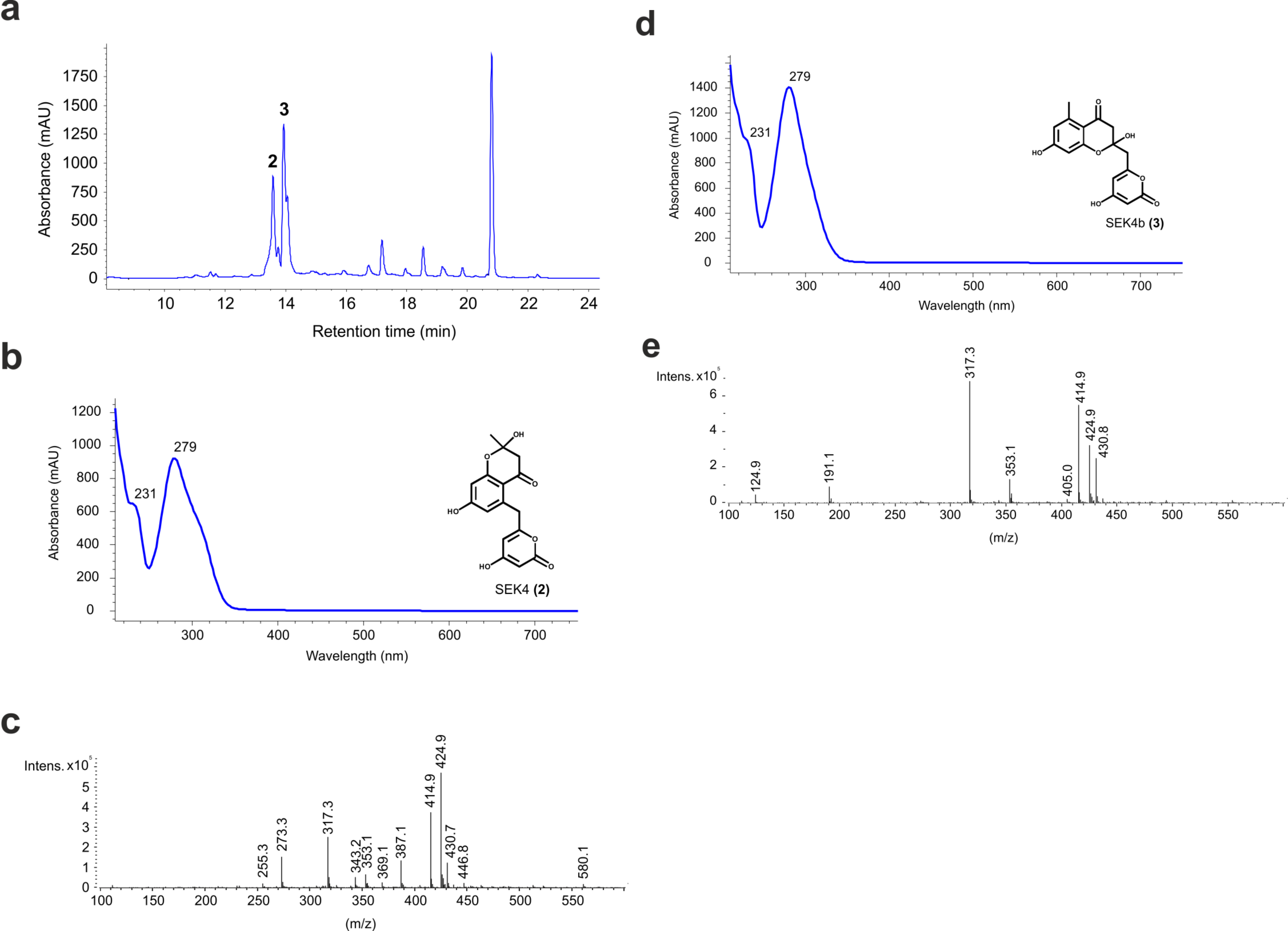

Supplement: S12 Fig — HPLC-UV-Vis-ESI-MS analysis of E. coli BL21(DE3) expressing antB-I, ΔAntA, identifying SEK4 and SEK4b as predominant octaketide shunt metabolites in the ΔKR anthraquinone biosynthetic pathway. (a) Typical chromatogram of culture supernatant from E. coli BL21(DE3) pACYCAntΔA at monitored 279 nm showing 2 peaks corresponding to SEK4 and SEK4b which are not present in E. coli BL21(DE3) or E. coli BL21(DE3) pACYCDuet-1. (b) UV-Vis spectrum for SEK4 (2) with λmax at 231 and 297 nm consistent with previously reported literature [10]. (c) ES− mass spectrum corresponding to SEK4 (2) observed [M-H]− 317.3, theoretical [M-H]− 317.1. (d) UV-Vis spectrum corresponding to SEK4b (3) with λmax of 231 and 297, also consistent with literature [10]. (e) As for panel c; ES− mass spectrum corresponding to SEK4b (3) observed deprotonated mass [M-H]− 317.3, theoretical mass is as for isomeric SEK4. Data presented represent 3 biological replicates and were acquired from E. coli BL21(DE3) pACYCAntΔAntA samples analysed in S10 Fig. UV-Vis, UV-visibility. (TIF) [file pbio.3000347.s012.tif]

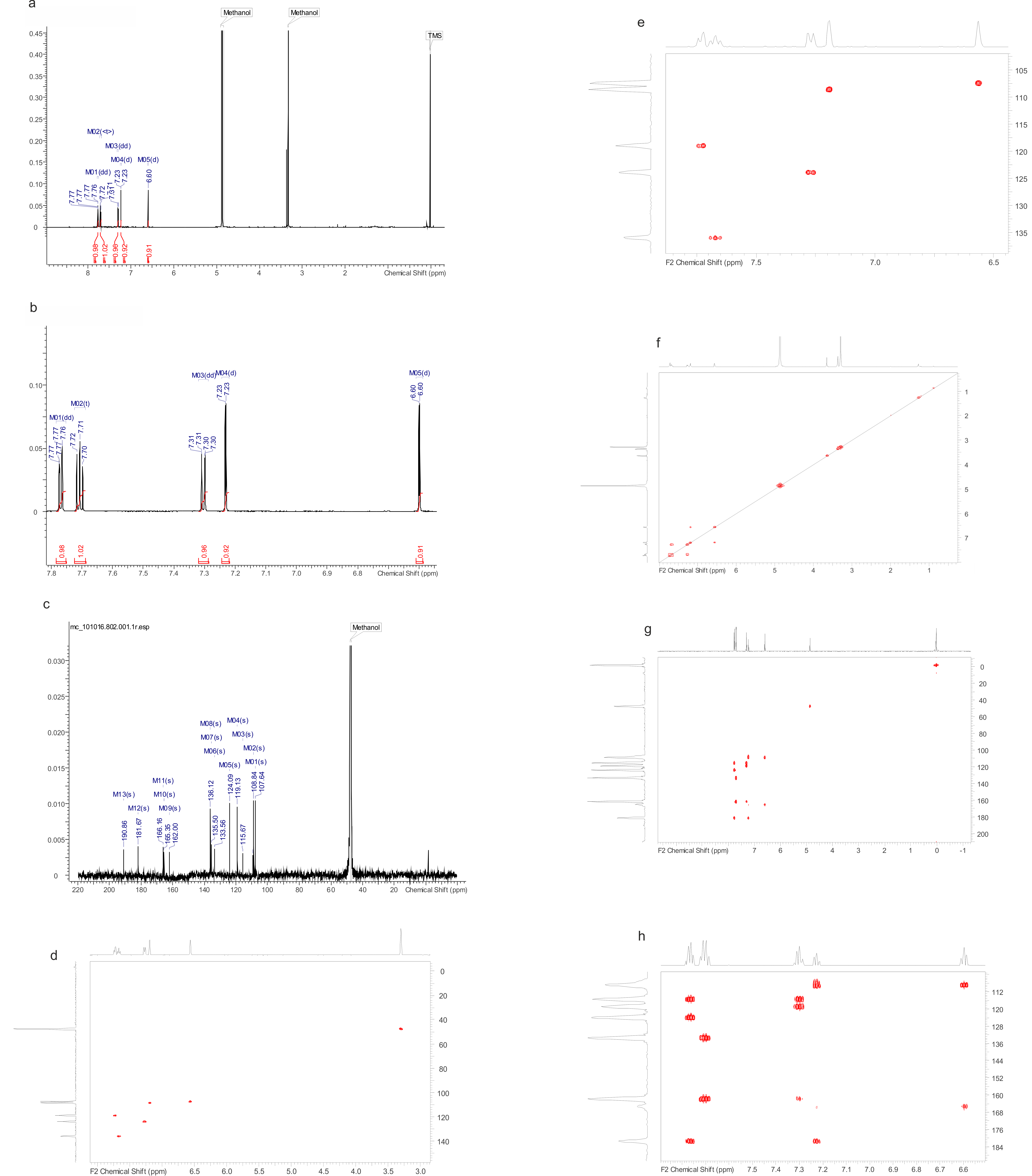

Supplement: S14 Fig — (a) Complete 1H NMR spectrum (400 MHz, methanol-d4, 298 K) in deuterated methanol with TMS and methanol peaks annotated. (b) Expanded 1H NMR spectrum showing aromatic proton signals. (c) 13C NMR spectrum (800 MHz, methanol-d4, 298 K) with TMS standard: the methanol solvent peak is annotated. (d) HSQC NMR spectrum (800 MHz, methanol-d4, 298 K). (e) Expansion of the HSQC spectrum showing relationship between aromatic protons and the corresponding carbon atoms. (f) A two-dimensional 1H-1H COSY NMR spectrum (800 MHz, methanol-d4, 298 K) showing proton–proton coupling. (g) HMBC NMR spectrum (800 MHz, methanol-d4, 298 K) with TMS standard. (h) Expansion of the HMBC NMR spectrum showing relationship between the aromatic protons and the corresponding carbon atoms. (TIF) [file pbio.3000347.s014.tif]

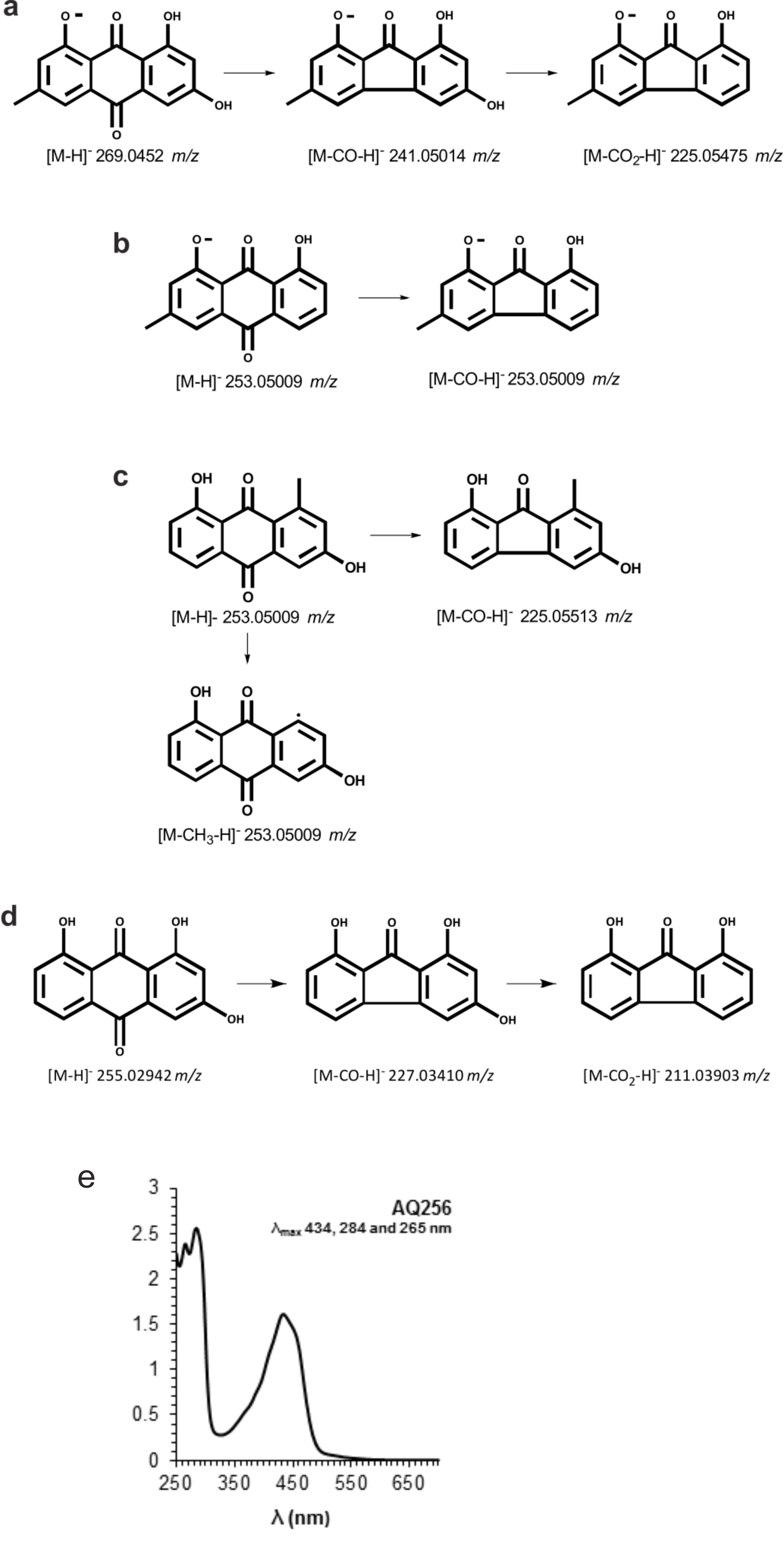

Supplement: S15 Fig — (a–d) Evaluation of chrysophanol, emodin, aloesaponarin II, and AQ256 MS-MS spectra (ES−). The measured MS-MS spectra values are depicted under each structure. (e) UV-Vis absorbance spectra for AQ256 (100% methanol), and 2 other anthraquinones, emodin and chrysophanol (80% methanol), recorded between 250 and 700 nm using a Cary 60 UV-Vis spectrophotometer (Agilent Technologies). All samples show common λmax at approximately 430 nm. UV-Vis, UV-visible. (TIF) [file pbio.3000347.s015.tif]

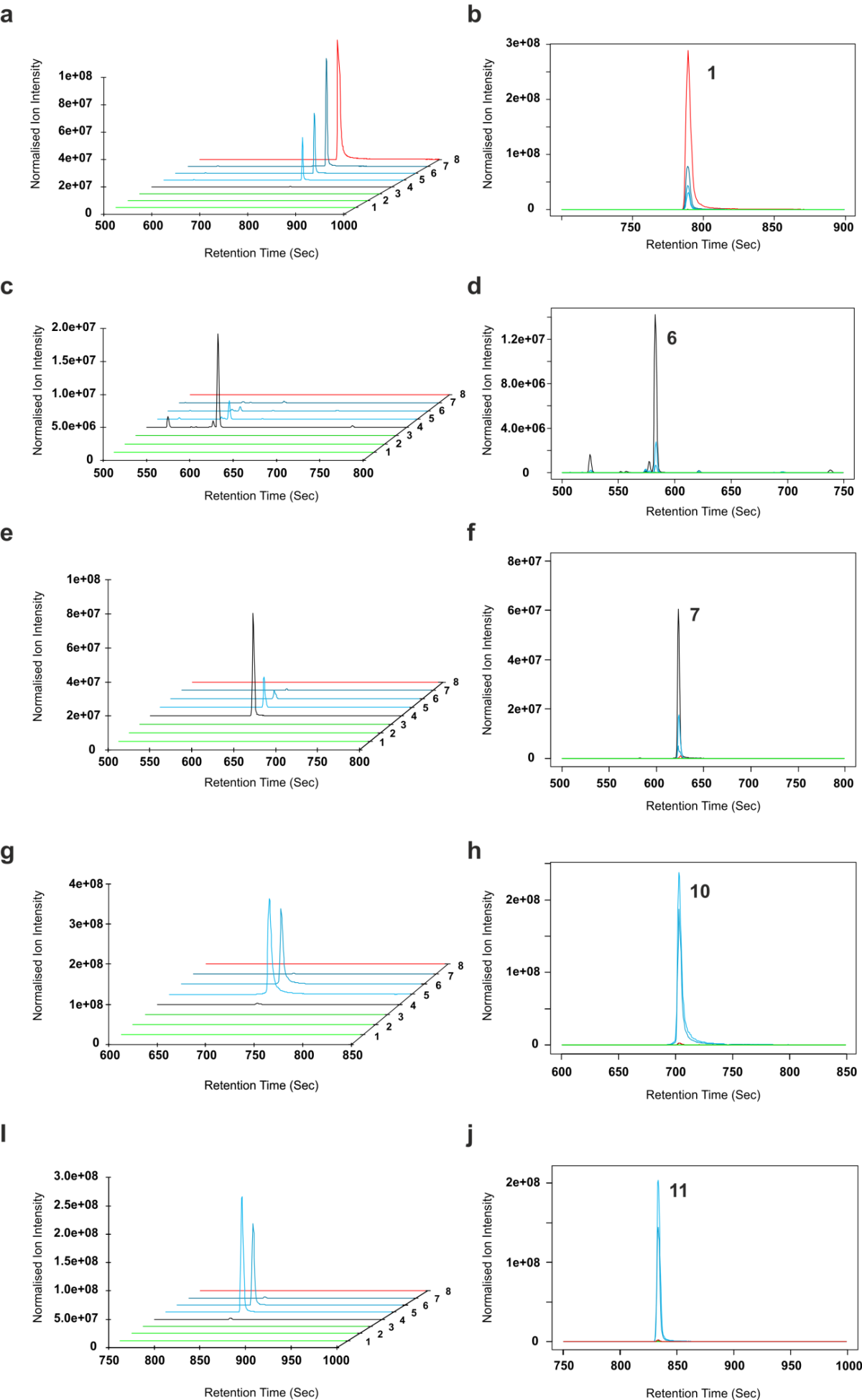

Supplement: S16 Fig — A comparison of EICs from the exometabolome of E. coli BL21(DE3) expressing the anthraquinone pathway complemented with ActIII, a KR from the actinorhodin biosynthetic pathway. All samples are numbered as media blank—1: E. coli BL21(DE3) wild type, 2: E. coli BL21(DE3) pACYCDuet-1, 3: E. coli BL21(DE3) pACYCAntΔAntA, 4: E. coli BL21(DE3) pACYCAntrefKR (refactored sequence), 5: E. coli BL21(DE3) pACYCAntwtKR (modified wild-type sequence), 6: E. coli BL21(DE3) pACYCAnthraquinone, and 7: Ion intensities were normalised to final cell density (OD600). Panels a and b show two-dimensional and three-dimensional EICs of all observable masses between 255.0286 and 255.0312 m/z: AQ256 theoretical mass of [M-H]− 255.02989. Panels c and d show EIC of masses between 317.0651 and 317.0683 m/z: SEK4 and SEK4b theoretical mass of [M-H]− 317.0667. All EICs use a ±5 ppm cutoff for identification of metabolite of interest and are representative of 3 biological samples, and HPLC conditions are as described in Materials and methods. AQ256 biosynthesis is observed in E. coli BL21(DE3) expressing antA-I, and restored in E. coli BL21(DE3) expressing AntB-I complemented with the actinorhodin KR but not in the ΔAntA host. Instead, the exometabolome of E. coli BL21(DE3) pACYCAntΔAntA is enriched in SEK4 and SEK4b, indicating a metabolic bottleneck to occur before ketoreduction of C9, as expected. Bolded numbers represent metabolites detailed in Fig 2. EIC, extracted ion chromatogram; KR, ketoreductase. (TIF) [file pbio.3000347.s016.tif]

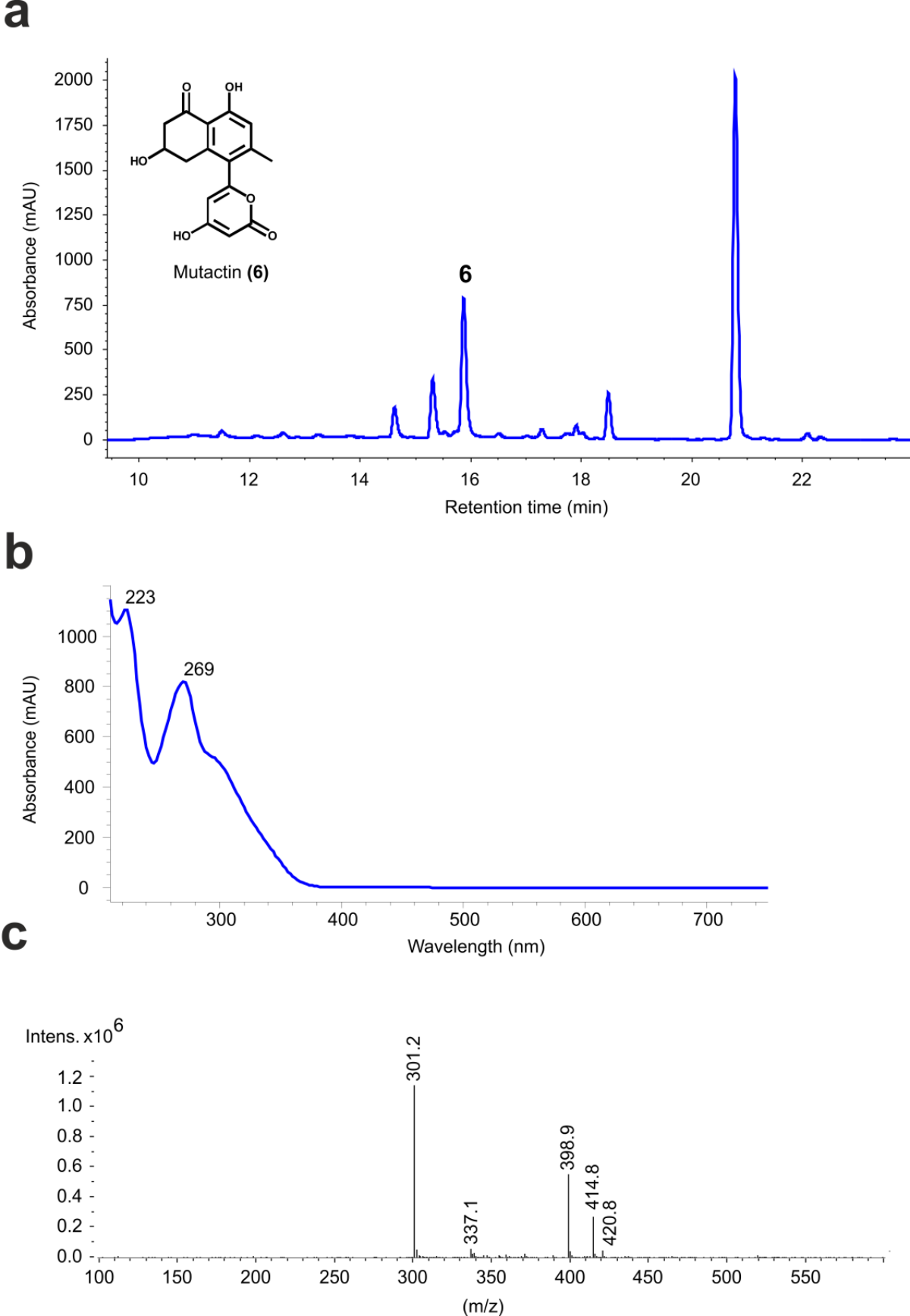

Supplement: S17 Fig — HPLC-UV-Vis-MS analysis of E. coli BL21(DE3) expressing antA-G, I, ΔAntH, identifying mutactin as predominant octaketide shunt metabolites in the ΔAntH anthraquinone biosynthetic pathway. All chromatographic conditions and methods are as described in Materials and methods. (A) Typical chromatogram of culture supernatant from E. coli BL21(DE3) pACYCAntΔH at 269 nm showing a peak corresponding mutactin which is not present in E. coli BL21(DE3) or E. coli BL21(DE3) pACYCDuet-1. (B) UV-Vis spectrum for mutactin (6) with λmax at 223 and 269 nm consistent with previously reported literature [1]. (C) ES− mass spectrum corresponding to mutactin (6) observed [M-H]− 301.2, theoretical [M-H]− 301.1. Data presented here are representative of 3 biological replicates and were acquired from E. coli pACYCAntΔAntH samples analysed in S10 Fig [56]. UV-Vis, UV-visible. (TIF) [file pbio.3000347.s017.tif]

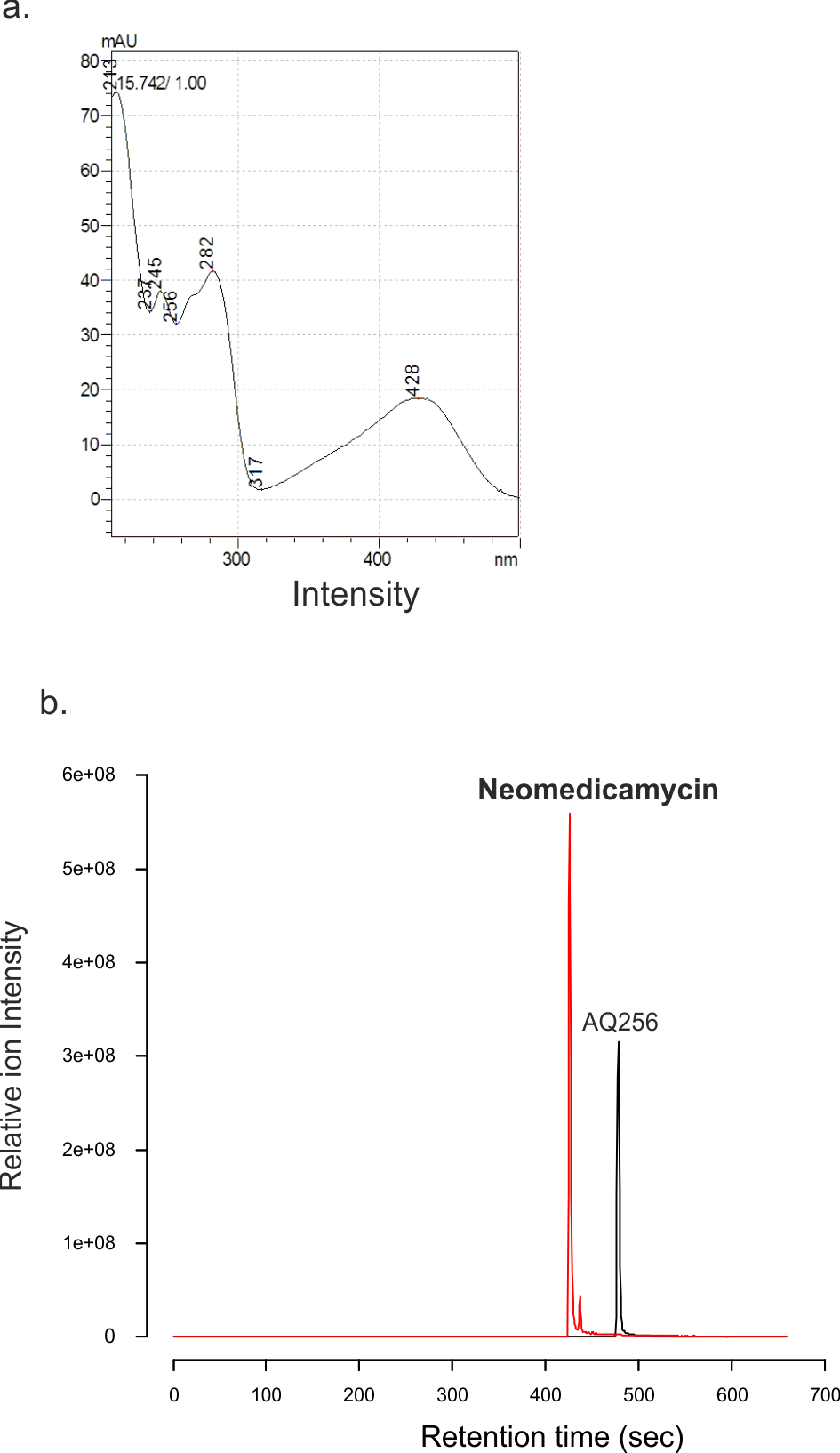

Supplement: S18 Fig — (A) UV-Vis absorbance spectrum for neomedicamycin in water:acetonitrile (0.05% TFA). λmax: 213, 245, 282, and 428 nm, λmin: 237, 256, and 317 nm. (B) EIC of neomedicamycin. Retention times for neomedicamycin tR: 426, AQ256 tR: 478. Exact masses neomedicamycin [M-H−]: 296.04568, 0.5 ppm from theoretical mass of 269.04555; AQ256 [M-H−]: 255.02983, 0.27 ppm from theoretical deprotonated mass of 255.02989. The red and black lines show EICs of 296.04568 ± 5 ppm and 255.02989 ± 5 ppm, respectively. Masses corresponding to methoxy-substituted AQ256 were not detectable in E. coli BL21(DE3) expressing antA-I. EIC, extracted ion chromatogram; UV-Vis, UV-visible. (TIF) [file pbio.3000347.s018.tif]

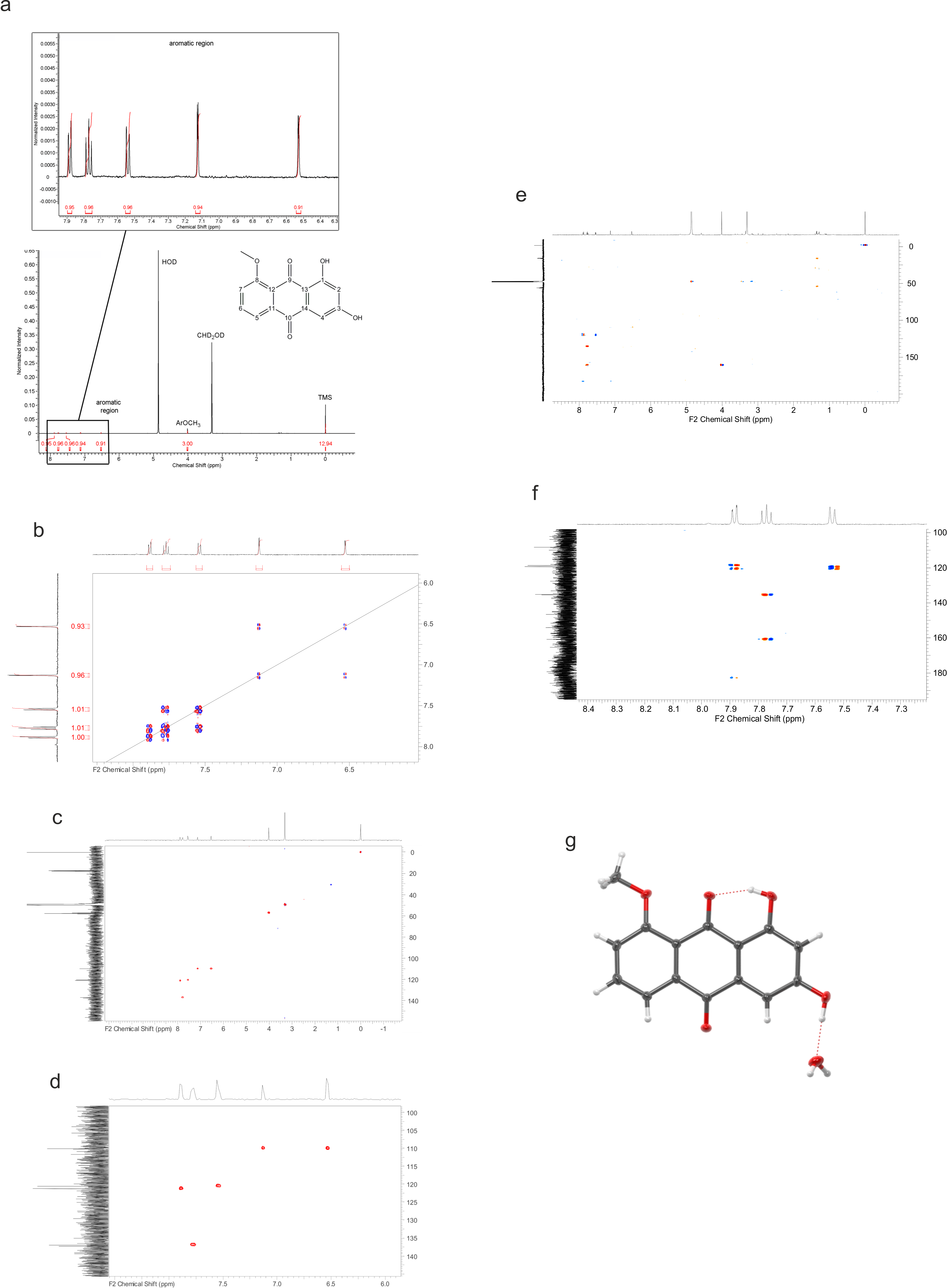

Supplement: S19 Fig — (A) 1H NMR spectrum (500 MHz, methanol-d4, 298 K). Values for integrated peak areas are denoted in red: TMS present at 0.03% vol/vol. Inset shows the expanded aromatic region. (B) COSY NMR spectrum (500 MHz, methanol-d4, 298 K). (C) HSQC NMR spectrum (500 MHz, methanol-d4, 298 K). (C) HMBC NMR spectrum (500 MHz, methanol-d4, 298 K). (D) X-ray crystal structure of neomedicamycin. Carbon = grey, oxygen = red, and hydrogen = white; image produced using POV-ray. TMS, Tetramethylsilane. (TIF) [file pbio.3000347.s019.tif]

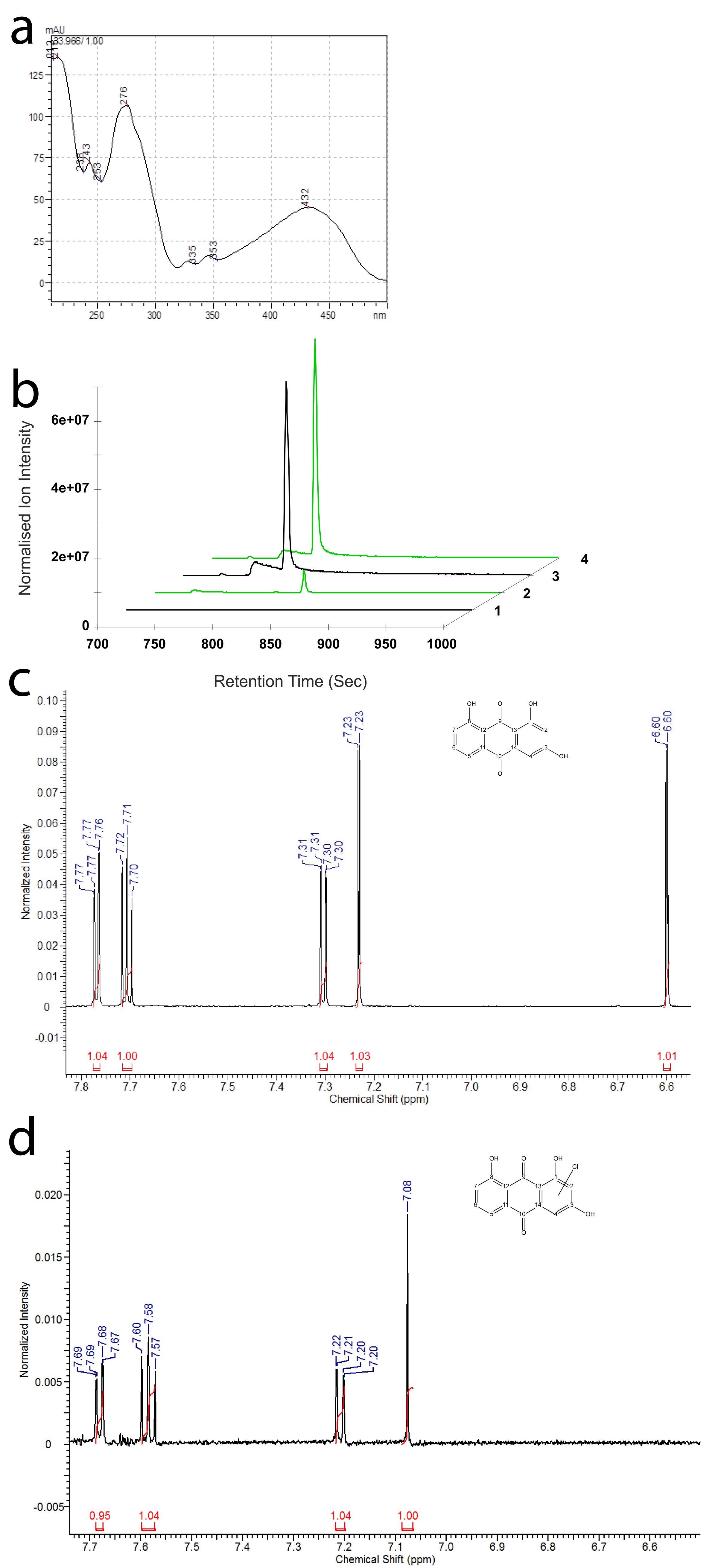

Supplement: S20 Fig — (A) UV-Vis absorbance spectrum for neochaetomycin in water:acetonitrile (0.05% TFA). λmax: 213, 243, 276, and 432 nm, λmin: 238, 253, 335, and 353 nm. (B) Chromatograms 1 and 2 represent EICs of all masses between 288.99092 ± 5 ppm from E. coli BL21(DE3) expressing antA-I and E. coli BL21(DE3) expressing antA-I and radH. A mass corresponding to neochaetomycin is detectable only in E. coli BL21(DE3) expressing the antA-I pathway as well as radH ([M-H]− 288.99066, 0.9 ppm for the theoretical mass of [M-H]− 288.99092). Chromatograms 3 and 4 represent EICs of all masses between 255.02989 ± 5 ppm from E. coli BL21(DE3) expressing antA-I and E. coli BL21(DE3) expressing antA-I and radH. AQ256 can be detected in both at similar intensities. EIC ion intensities were normalised to final cell densities to enable comparison. Comparison of (C) the 1H NMR spectrum for AQ256 (400 MHz, methanol-d4, 298 K) to (D) the 1H NMR spectra of neochaetomycin (600 MHz, methanol-d4, 298 K). This comparison suggests that chlorination occurs at position 2, as the resonance at 6.60 ppm assigned to this proton in AQ256 disappears. However, this tentative assignment cannot be confirmed until further characterisation data are obtained. UV-Vis, UV-visible. (TIF) [file pbio.3000347.s020.tif]

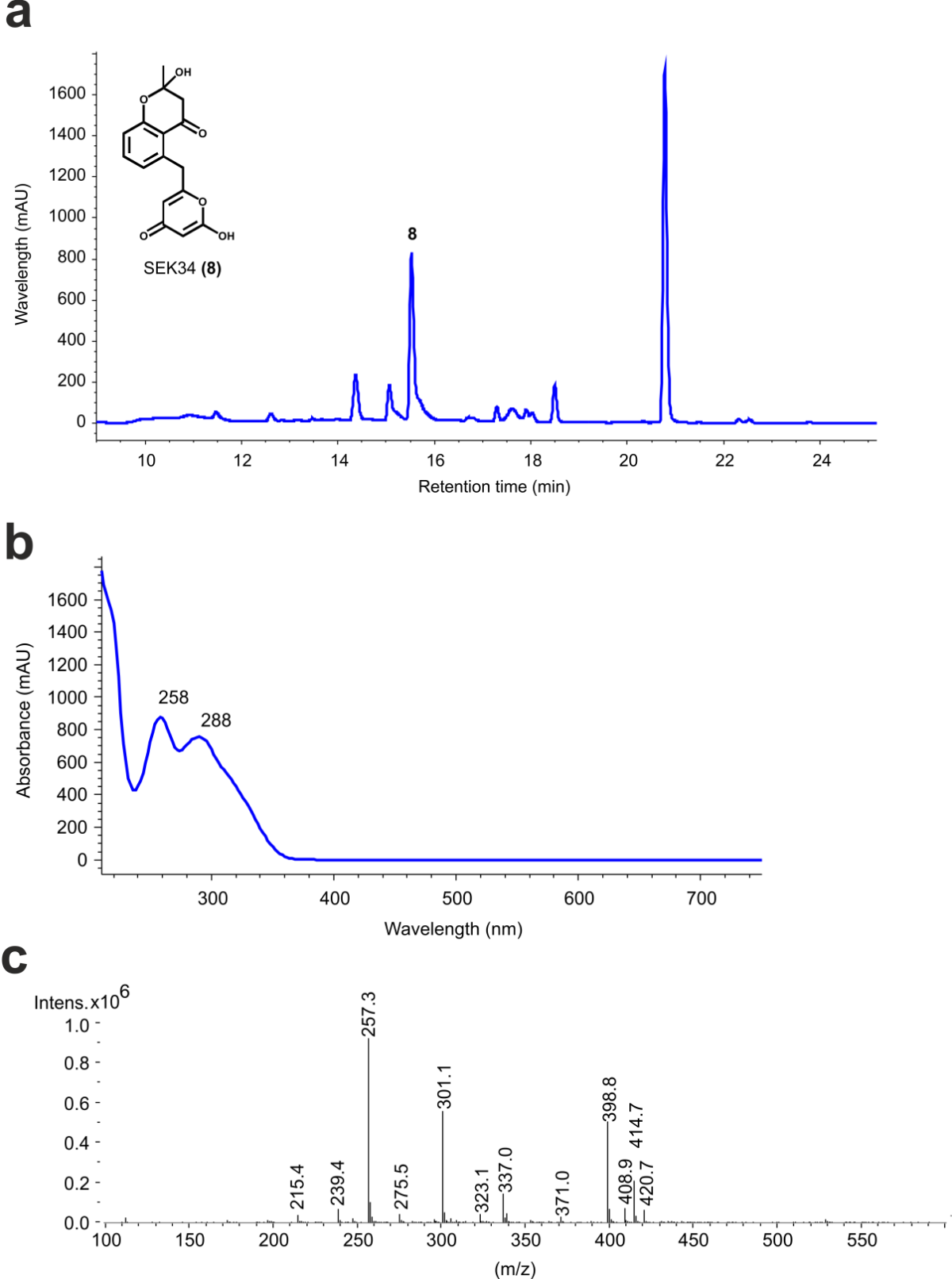

Supplement: S21 Fig — HPLC-UV-Vis-MS analysis of E. coli BL21(DE3) expressing antA, B, D-I, ΔAntC, identifying SEK34 as predominant octaketide shunt metabolites in the ΔAntC anthraquinone biosynthetic pathway. Chromatographic conditions and methods are as described in Supplementary Figure 38. (A) Typical chromatogram of culture supernatant from E. coli BL21(DE3) pACYCAntΔC at 258 nm showing a peak corresponding SEK34 which is not present in E. coli BL21(DE3) or E. coli BL21(DE3) pACYCDuet-1. (B) UV-Vis spectrum for SEK34 (8) with λmax at 258 and 288 nm consistent with previously reported literature [1]. (C) ES− mass spectrum corresponding to SEK34 (8) observed [M-H]− 301.1, theoretical [M-H]− 301.1, presence of an abundant mass at 257.3 m/z is also previously reported for SEK34. Data presented are in strong agreement with 2 additional biological replicates and were acquired from E. coli BL21(DE3) pACYCAntΔAntC samples analysed in S10 Fig [56]. (TIF) [file pbio.3000347.s021.tif]

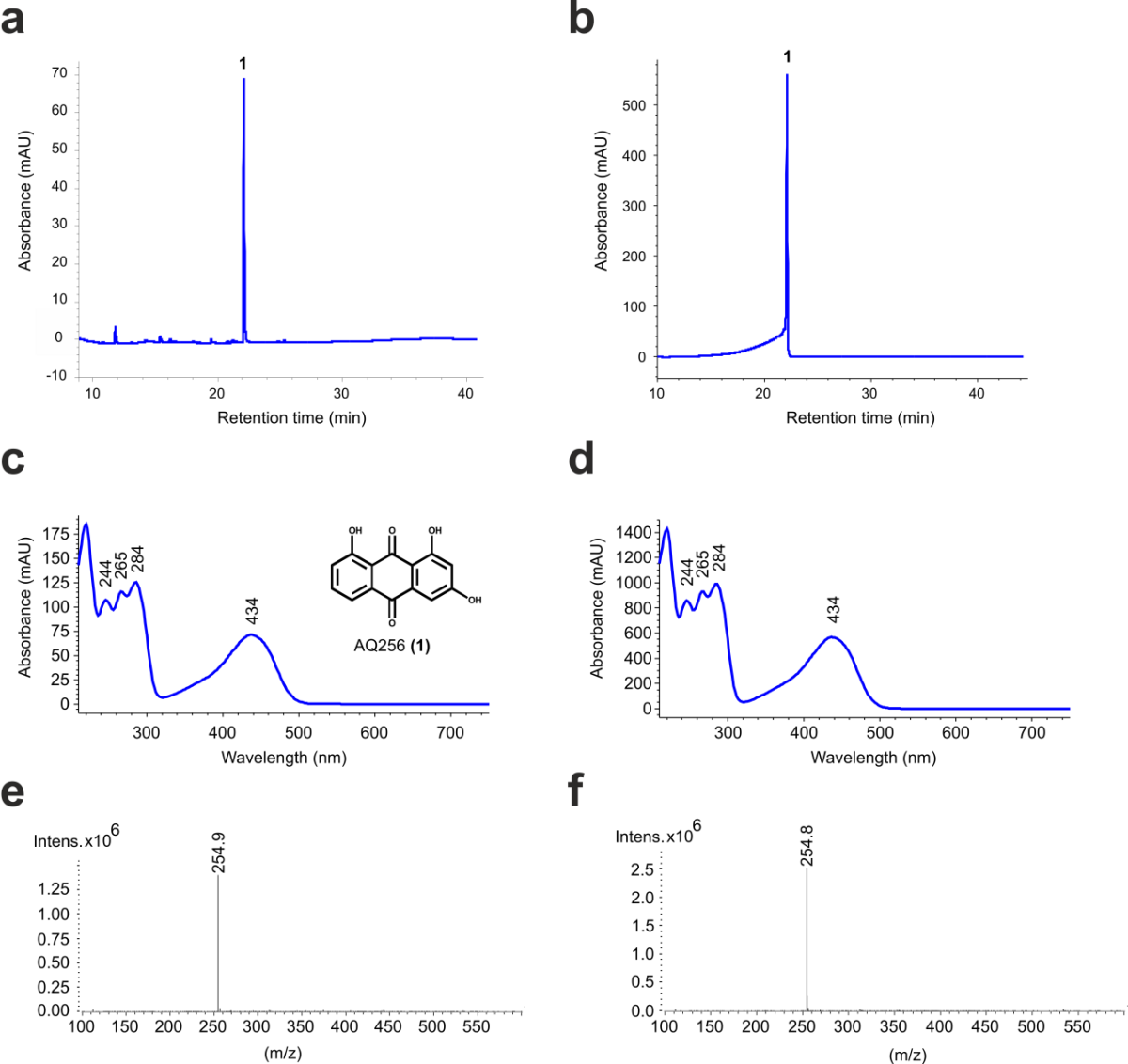

Supplement: S22 Fig — HPLC-UV-Vis-MS analysis of E. coli BL21(DE3) expressing antA-I identifying AQ256 as the predominant end compound of the anthraquinone biosynthetic gene cluster. Data are representative of the same 3 biological replicates. All chromatographic conditions and methods are as described in Materials and methods. (A) Typical chromatogram of culture supernatant from E. coli BL21(DE3) pACYCAnthraquinone monitored at 434 nm showing a peak corresponding AQ256 which is not present in E. coli BL21(DE3) or E. coli BL21(DE3) pACYCDuet-1. Additionally, no other major peaks are present at this wavelength indicating this to be the only anthraquinone produced. (B) Typical chromatogram of AQ256 analytical standard purified from E. coli BL21(DE3) pACYCAnthraquinone and characterised by 1H, 13C, COSY, HSQC, and HMBC NMR spectroscopy. (C) UV-Vis spectrum for AQ256 with λmax at 244, 265, 284, and 434 nm consistent with the counterpart UV-Vis spectrum of the AQ256 standard in D. (E and F) ES− mass spectrum corresponding to AQ256 derived from E. coli BL21(DE3) pACYCAnthraquinone and AQ256 analytical standard, respectively. Observed masses are [M-H]− 254.9 and [M-H]− 254.8, theoretical mass [M-H]− 255. UV-Vis and mass spectra are in good agreement between experimental samples and analytical standards. UV-Vis, UV-visible. (TIF) [file pbio.3000347.s022.tif]
