# Supplementary material for: Assembling a plug-and-play production line for combinatorial biosynthesis of aromatic polyketides in Escherichia coli
Source: PLoS Biol. 2019 Jul 18;17(7):e3000347. doi: 10.1371/journal.pbio.3000347 (PMC6638757; doi:10.1371/journal.pbio.3000347)
Supplement: S1 Table — Underlined organisms contain characterised BGCs. Coloured fields show organisms comprising BGCs predicted to produce the same or extremely similar specialised metabolites. Type II PKS BGCs from the underlined organisms are selected for this study. *K. racemifer comprises 3 predicted type II PKS BGCs: two satisfy manual curation criteria. (DOCX) [file pbio.3000347.s023.docx]

S1 Table

| **AntD - Cluster No.** | |  | **AntE - Cluster No.** | |  |
| --- | --- | --- | --- | --- | --- |
| **antiSMASH 3.0** | **Manual curation** | **Organism** | **antiSMASH 3.0** | **Manual curation** | **Unique BGC** |
| 2 | 2 | *Delftia acidovorans* | 3 | 3 | 1 |
| 1 | 1 | *Acidithiobacillus ferrivorans* | 1 | 1 |  |
| 1 | 1 | *Azospirillum brasilense* | 1 | 1 |  |
| 2 | 2 | *Blautia* spp. | 1 | 1 | 1 |
| 1 | 1 | *Lachnospiraceae bacterium* 3-1 | 1 | 1 |  |
| 0 | 0 | *Lactobacillus salivarius* | 1 | 1 | 1 |
| 0 | 0 | *Lactobacillus oris* | 1 | 1 |  |
| 0 | 0 | *Streptoccocus* spp. | 1 | 1 |  |
| 1 | 1 | Candidatus *Desulfofervidus auxilii* | 1 | 1 | 1 |
| 1 | 1 | *Deltaproteobacteria bacterium* RBG_16_54_11 | 1 | 1 |  |
| 1 | 1 | *Desulfobacterium* sp. 4572_20 | 0 | 0 |  |
| 1 | N/A | ***Clostridium beijerinckii*** | 1 | N/A | Reported |
| 1 | N/A | ***Clostridium puniceum*** | 1 | N/A |  |
| 2 | 2 | *Bacillus endophyticus* | 0 | 0 | 1 |
| 1 | 1 | *Dendrosporobacter quercicolus* | 1 | 1 | 1 |
| 1 | 1 | *Gloeocapsa* sp. PCC 7428 | 0 | 0 | 1 |
| 1 | 1 | *Hoeflea* sp. IMCC20628 | 1 | 1 | 1 |
| 1 | 2 | *Ktedonobacter racemifer** | 1 | 2 | 2 |
| 11 | 5 | *Omnitrophica* spp. | 11 | 8 | 1 |
| 4 | 4 | *Pseudoalteromonas luteoviolacea* | 8 | 8 | 1 |
| 15 |  | ***Photorhabdus* spp.** | 10 |  | Reported |
| 52 |  | *Actinobacteria* | 3 |  | N/A |
| 100 | 28 | **Total** | 49 | 34 | 12 |
