# Supplementary material for: Assembling a plug-and-play production line for combinatorial biosynthesis of aromatic polyketides in Escherichia coli
Source: PLoS Biol. 2019 Jul 18;17(7):e3000347. doi: 10.1371/journal.pbio.3000347 (PMC6638757; doi:10.1371/journal.pbio.3000347)
Supplement: S2 Table — A table displaying the gatekeeper residues from a series of CLFs with bulky R-groups which sterically reduce the size of the amphipathic tunnel at the KS/CLF dimer interface [1]. Residue order represents their proximity to the cavity entrance. Red residues define the bottom of the cavity, while blue AAs are smaller residues from homologues producing longer polyketides. Gatekeeper residues do not map to the anthraquinone sequence; prediction of chain length using this method suggests the nascent poly-β-ketide to be C20 [57]. (DOCX) [file pbio.3000347.s024.docx]

| **Host organism Gram stain** | **Cluster** | **Chain length factor gate keeper residues** | | | | | **Chain length** | **Conserved Q residue** |
| --- | --- | --- | --- | --- | --- | --- | --- | --- |
|  |  | 112 | 116 | 194 | 109 | 193 |  | 161 |
| Gram + | Actinorhodin | T | F | W | F | G | C16 | Q |
| Gram + | Oxytetracycline | G | L | W | F | G | C18 | Q |
| Gram + | WhiE spore pigment | G | L | I | G | S | C24 | D |
| Gram + | Griseorhodin | G | M | L | T | G | C26 | Q |
| Gram - | Anthraquinone | I | V | - | W | - | C16 (putative) | D |
