# Supplementary material for: Assembling a plug-and-play production line for combinatorial biosynthesis of aromatic polyketides in Escherichia coli
Source: PLoS Biol. 2019 Jul 18;17(7):e3000347. doi: 10.1371/journal.pbio.3000347 (PMC6638757; doi:10.1371/journal.pbio.3000347)
Supplement: S3 Table — All theoretical masses used in this study are listed. Isomers are highlighted in corresponding colours. All masses are reported as atomic mass units. (DOCX) [file pbio.3000347.s025.docx]

| **Name** | **Molecular formula** | **Exact mass** | **[M+H]^+^** | **[M-H]^-^** |
| --- | --- | --- | --- | --- |
| SEK4 | C_16_H_14_O_7_ | 318.0740 | 319.0812 | 317.0667 |
| SEK4b | C_16_H_14_O_7_ | 318.0740 | 319.0812 | 317.0667 |
| B26 | C_16_H_12_O_6_ | 300.0634 | 301.0707 | 299.0561 |
| AUR367 | C_16_H_12_O_6_ | 300.0634 | 301.0707 | 299.0561 |
| Mutactin | C_16_H_14_O_6_ | 302.0790 | 303.0863 | 301.0718 |
| Dehydromutactin | C_16_H_12_O_5_ | 284.0685 | 285.0758 | 283.0612 |
| SEK34 | C_16_H_14_O_6_ | 302.0790 | 303.0863 | 301.0718 |
| SEK34b | C_16_H_12_O_5_ | 284.0685 | 285.0758 | 283.0612 |
| Aloesaponarin II | C_15_H_10_O_4_ | 254.0579 | 255.0652 | 253.0506 |
| DMAC | C_16_H_10_O_6_ | 298.0477 | 299.0550 | 297.0405 |
| (s)-DNPA | C_16_H_14_O_5_ | 286.0841 | 287.0914 | 285.0768 |
| AQ256 | C_14_H_8_O_5_ | 256.0372 | 257.0445 | 255.0299 |
| AQ270a | C_15_H_10_O_5_ | 270.0528 | 271.0601 | 269.0455 |
| AQ270b | C_15_H_10_O_5_ | 270.0528 | 271.0601 | 269.0455 |
| AQ284a | C_16_H_12_O_5_ | 284.0685 | 285.0758 | 283.0612 |
| AQ284b | C_16_H_12_O_5_ | 284.0685 | 285.0758 | 283.0612 |
| AQ300 | C_16_H_12_O_6_ | 300.0634 | 301.0707 | 299.0561 |
| AQ314 | C_17_H_14_O_6_ | 314.0790 | 315.0863 | 313.0718 |
| 1,3,8-trihydroxyanthracen-9(10H)-one | C_14_H_10_O_4_ | 242.0579 | 243.0652 | 241.0506 |
| 1,3,8-trihydroxy-Dianthrone | C_28_H_18_O_8_ | 482.10017 | 483.1074 | 481.0929 |
