# Supplementary material for: Assembling a plug-and-play production line for combinatorial biosynthesis of aromatic polyketides in Escherichia coli
Source: PLoS Biol. 2019 Jul 18;17(7):e3000347. doi: 10.1371/journal.pbio.3000347 (PMC6638757; doi:10.1371/journal.pbio.3000347)
Supplement: S4 Table — Primer nomenclature is typically gene/region amplified_direction of amplification_restriction endonuclease site. All primers with additional 5′ RE sequences are preceded with an additional random 6 bp sequence to facilitate PCR product digestion. RE recognition sequences are bolded. Primers were designed and verified with IDT oligoanalyser. RE, restriction endonuclease. (DOCX) [file pbio.3000347.s026.docx]

| **Primer name** | **Primer sequence 5’-3’** |
| --- | --- |
| Plu4189_rev_XhoI | atcagt**ctcgag**atttttatcgtttaaacttgatgag |
| Plu4191_for_BglII | ccgctc**agatct**aatgataataaataacagaaatgaatctc |
| Plu4191_rev_KpnI | atgcat**ggtacc**ttacgcattgtctact |
| plu4190_for_EcoRI | atgcat**gaattc**tcgtaaaagagttgtc |
| Plu4190_rev_PstI | tcgcat**ctgcag**ttaaatagctgaaaaact |
| Plu4193_for_NcoI untagged | gcatgc**ccatgg**gcgacgatatttctttatcatctga |
| Plu4193_rev_HindIII | gcatgc**aagctt**ttactcatctttgttccttataatctc |
| Plu4188_for_NdeI | gttgtc**catatg**aaactaatctctatgttgttacattcag |
| Plu4188_rev_XhoI | ttatac**ctcgag**ttattgattcctcaatgtaaatagttttttagga |
| Plu4194_for_NdeI | gcagag**catatg**aaatatgcctttattaccggc |
| Plu4192_rev_XhoI | tatact**ctcgag**ttataatattgcgaccactcgactc |
| AQBGC_Rev_PstI | gcatgc**ggatcc**cgaacatcaaacatagcgcaaatatt |
| plu4186Δ_for | taaaattttaatatactaaatacgagtgtctaaccactctttgc |
| plu4186Δ _rev | tatattaaaattttaaatatttgcgctatgtttgatgttcg |
| KR_swap_IF | atgtatatctccttcttatacttaactaatatactaagatg |
| Plu4194_del_IF_for | tgtaaccgctcaagcacttaat |
| Sco5086_for_IF | gaaggagatatacatatggcgacccaggactccgaagtcgcactg |
| Sco5086_rev_IF | gcttgagcggttacagcggccgcaccgggcccgat |
| Ref_Sco5086_IF_for | gaaggagatatacatatggcaacccaggatagcgaagtt |
| Ref_Sco5086_IF_rev | gcttgagcggttacagctgctgcacccgga |
| plu4187_replace_fw | taacttaaaggaaaataagatgaataat |
| plu4188_rev | ttattgattcctcaatgtaaatagttttttagga |
| Sco5090_for_IF | ttacattgaggaatcaataatgagccggccgggagaac |
| Sco5090_rev_IF | cttattttcctttaagttatcatgacgccggcccg |
| Ref_Sco5090_IF_for | ttacattgaggaatcaataatgagccgtccgggtgaaca |
| Ref_Sco5090_IF_rev | cttattttcctttaagttattagcttgccggaccagcg |
| Plu4187_delta_for | acttaggcataaacctaacttaaaggaaaataagatgaataat |
| Plu4187_delta_rev | ggtttatgcctaagtttattgattcctcaatgtaaatagttttttagga |
| Plu4186Δ_for | taaaattttaatatactaaatacgagtgtctaaccactctttgc |
| Plu4186Δ _rev | tatattaaaattttaaatatttgcgctatgtttgatgttcg |
| Plu4192Δ_for | attttagtcatgatactcgagtctggtaaagaaacc |
| Plu4192Δ _rev | tatcatgactaaaatttactcatctttgttccttataatc |
