# Supplementary material for: Assembling a plug-and-play production line for combinatorial biosynthesis of aromatic polyketides in Escherichia coli
Source: PLoS Biol. 2019 Jul 18;17(7):e3000347. doi: 10.1371/journal.pbio.3000347 (PMC6638757; doi:10.1371/journal.pbio.3000347)
Supplement: S5 Table — All plasmids used and constricted in this study are shown. *Sequence optimised using GeneArt GeneOptimiser (ThermoFischer Scientific, Massachusetts, US) and synthesised by Gen9 (Massachusetts, US). (DOCX) [file pbio.3000347.s027.docx]

| **Plasmid name** | **Insert Description** | **Backbone description** | **Source** |
| --- | --- | --- | --- |
| pACYCDuet-1 | **-** | p15A ori, *cat* (Cm^R^), T7 promoters | Novagen |
| pETDuet-1 | **-** | pBR322 ori, *bla* (Ap), T7 promoters | Novagen |
| pG9m-2-ActKRRef | Refactored *Sco*5086, codon optimised for expression in *E. coli* | pUC19 ori, Amp^R^ | This study* |
| pG9m-2-ActARO/CYCRef | Refactored *Sco*5090, codon optimised for expression in *E. coli* | pUC19 ori, Amp^R^ | This study* |
| pBbA2k-RFP | RFP | p15A ori, kan/Neo (Kn^R^/Neo^R^), pTet | ^22^ |
| pBbB1a-GFP | GFP | BBR1 ori, *bla* (Ap), T7 promoter | ^22^ |
| pBbA2k-plumPKS | *plu*4191, *plu*4190, *plu*4189 | p15A ori, kan/Neo (Kn^R^/Neo^R^), pTet | This study |
| pBbB1a-plumPKS | *plu*4191, *plu*4190, *plu*4189 | BBR1 ori, *bla* (Ap), T7 promoter | This study |
| pETDuetPlu419091 | *plu*4191, His6:*plu*4190 | pBR322 ori, *bla* (Ap), T7 promoters | This study |
| pACYCPlu4188 | *plu*4188 | p15A ori, *cat* (Cm^R^), T7 promoters | This study |
| pACYCPlu418893 | *plu*4188, *plu*4193 |  | This study |
| pACYCDuetPlu4192-94 | *plu*4194, 93, 92 |  | This study |
| pACYCAnthraquinone | *plu*4194, 93, 92, and *plu*4091, 90, 89, 88, 87, 86 |  | This study |
| pACYCAnt**ref**KR | *plu*4193, 92, and *plu*4091, 90, 89, 88, 87, 86, (Δ94), and refactored *Sco*5086, |  | This study |
| pACYCAnt**wt**KR | *plu*4193, 92, and *plu*4091, 90, 89, 88, 87, 86, (Δ94), and wild type *Sco*5086 |  | This study |
| pACYCAntΔAntA | *plu*4193, 92, and *plu*4091, 90, 89, 88, 87, 86, (Δ94) |  | This study |
| pACYCAnt**ref**CYC | *plu*4194, 93, 92, and *plu*4091, 90, 89, 88, 86, (Δ87) refactored *Sco*5090 |  | This study |
| pACYCAnt**wt**CYC | *plu*4194, 93, 92, and *plu*4091, 90, 89, 88, 86, (Δ87) wild type *Sco*5090 |  | This study |
| pACYCAntΔAntH | *plu*4194, 93, 92, and *plu*4091, 90, 89, 88, 86, (Δ87) |  | This study |
| pACYCAntΔAntC | *plu*4194, 93 and *plu*4091, 90, 89, 88, 87 86, (Δ92) |  | This study |
| pACYCAntΔAntI | *plu*4194, 93 and *plu*4091, 90, 89, 88, 87, (Δ86) |  | This study |
| pETIFMT | Isoflavone *O-*methyltransferase *Medicago truncatula* | ColE1 ori, Kan^R^, T7 promoter | Provided by Binuraj Menon |
| pET28b-RadH | *radH,* *Chaetomium chiversii* |  |  |
| pETM-11b AntB | *plu4193* (AntB, PPTase) |  | This study |
| pHOLDKraAB | Refactored *kra* KS and CLF | ColE1 ori, *bla* (Ap), holding vector: no promoters | This study |
| pHOLDDacAB | Refactored d*ac*KS and CLF |  | This study |
| pHOLDSspAB | Refactored *ssp* KS and CLF |  | This study |
| pHOLDDauAB | Refactored *dau* KS and CLF |  | This study |
| pHOLDBendAB | Refactored *bend* KS and CLF |  | This study |
| pHOLDPluAB | Refactored *plu* KS and CLF |  | This study |
| pHOLDRemAB | Refactored *rem* KS and CLF |  | This study |
| pHOLDOvmPK | Refactored *ovm* KS and CLF |  | This study |
| pETDacAB | Refactored d*ac*KS and His^6^CLF | pBR322 ori, *bla* (Ap), T7 promoters | This study |
| pETDacB | Refactored d*ac* His^6^CLF |  | This study |
| pETSspAB | Refactored *ssp* His^6^KS and CLF |  | This study |
| pETSspA | Refactored *ssp* His^6^KS |  | This study |
| pETPluAB | Refactored *plu* KS and His^6^CLF |  | This study |
| pETBendAB | Refactored *bend* KS and His^6^CLF |  | This study |
| pETDauAB | Refactored *dau* KS and His^6^CLF |  | This study |
| pETKraAB | Refactored *kra* KS and His^6^CLF |  | This study |
| pETKraB | Refactored *kra* His^6^CLF |  | This study |
| pETRemAB | Refactored *rem*KS and His^6^CLF |  | This study |
| pETRemB | Refactored *rem* His^6^CLF |  | This study |
| pETOvmPK | Refactored *ovm* KS and His^6^CLF |  | This study |
| pETOvmK | Refactored *ovm* His^6^CLF |  | This study |
