# Supplementary material for: Assembling a plug-and-play production line for combinatorial biosynthesis of aromatic polyketides in Escherichia coli
Source: PLoS Biol. 2019 Jul 18;17(7):e3000347. doi: 10.1371/journal.pbio.3000347 (PMC6638757; doi:10.1371/journal.pbio.3000347)
Supplement: S1 Text — Detailed bioinformatic analysis of the actinobacterial KS and CLF. (DOCX) [file pbio.3000347.s028.docx]

## S1 Text. Bioinformatics analysis

Detailed bioinformatic analysis of the actinobacterial ketosynthase (KS) and chain length factor (CLF).

## AntE; a partner for AntD

AntE does not comply with two commonly observed properties of chain length factors defined in the literature. Gatekeeper residues in the CLF, which define the volume of the solvent excluded cavity at the dimer interface into which the polyketide chain grows, do not map onto AntE, and the highly conserved glutamine (Q161) residue important in decarboxylation of the malonyl-ACP starter unit substrate initiating biosynthesis is substituted for an aspartic acid residue (S2 Fig). Together, these two deviations raise questions regarding AntE’s function as a component of the anthraquinones minimal polyketide synthase.

To gain a deeper insight into the secondary and tertiary structure of AntE, a homology model was constructed using an actinorhodin (*act*) CLF backbone (S1 Fig). The homology model maintains the significant dissimilarity to ActI ORFII at the C-terminus, as expected, and while this does not comprise the region proposed to form the amphipathic tunnel, into which the polyketide grows, it does encompass a disordered region at the hetereodimer interface. The region of dissimilarity may be important for ACP:CLF protein-protein interactions which, in canonical systems, is responsible for delivering acylated ACPs to the glutamine active residue catalysing decarboxylation of the polyketide starter unit. In agreement with this, residues shown to be important in ACP:FabF protein–protein interactions[[1](#_ENREF_1)] do not map to AntE (S1 Fig). The anthraquinone cluster also harbours its own putative CoA ligase, this is a common feature of biosynthetic pathways which utilise a non-acetate starter unit suggesting the starter-unit-primed ACP may not require decarboxylation, or may be decarboxylated via an alternative as yet unknown mechanism. If substrate decarboxylation is not catalyzed by AntE the significantly different C-terminus may reduce competitive and unfavourable interactions between AntE and AntF promoting end compound biosynthesis.

Interestingly, protein secondary structure predictions (Jpred4[[2](#_ENREF_2)]) show secondary structural motifs of AntE to have a similar organization to that observed in FabF, despite significant amino acid sequence dissimilarity at the C-terminus. The second beta sheet, β13 which form part of the major structural αβαβα thiolase fold common to fatty acid synthases and polyketide synthases, is not predicted in AntE homology models; rather an open solvent exposed structure is proposed with low confidence. In contrast, secondary structure predictions using Jpred4 indicate this structure to be maintained; the AntE AA sequence is predicted to form beta strands at positions corresponding to β8, β13 (QIIIQR) and β14 indicating the αβαβα fold topology of FabF, *act* CLF and other KSs and CLFs to be consistent in AntE (S2 Fig). Unlike the homology models, protein secondary structure predictions were not modelled on existing crystal structures.

### Identification of KS/CLF sequences from underexplored phyla

All available prokaryotic genomes were downloaded from the NCBI Reference Sequence Database (RefSeq) assembly summary for bacteria (August, 2016) for BGC mining. The RefSeq summary comprises 7809 complete genomes, 48,197 contigs, 45,782 scaffolds and 1443 chromosomes. To reduce computational burden, 1 in 3 ‘complete genomes’ accession numbers were chosen randomly, resulting in a dataset of 2556 nucleotide sequences. This dataset was further refined by removing any entries defined as ‘Contigs’ or ‘Chromosome’. Four entries were removed (Contig titles: *Escherichia coli* NO114, *Escherichia coli* and *Clostridum sporogenes*. Chromosome titles: *Leptospira borgpetersenii* str. 4E) resulting in a dataset comprising 2552 nucleotide sequences corresponding to prokaryotic genomes. These sequences were analysed by antiSMASH 3.0.5 locally. Genomes within the dataset provided good taxonomic coverage including, but not limited to, 333 representative sequences from the actinobacterial phylum, 568 from Firmicutes, and 1086 from Proteobacteria. NRPS containing clusters were the most abundant single class of BGC, representing more than 17% of all BGCs identified, and more than 26% when hybrid clusters were considered. In all, 13,183 BGCs were predicted from the 2552 genomes analysed, comprising a vast sequence space totalling 7,050,113,326 bp, predicting 1.87 BGCs per Mb of genomic DNA.

Phylogenetic trees were constructed using a multi-locus sequence approach. Gene sequences for 16S RNA, *recA* and *gyrB* were aligned individually using MUSCLE before being clipped and concatenated manually to generate one hybrid sequence representative of all three genes, per organism. Concatenated sequences were aligned as above and used to construct maximum likelihood phylogenetic trees using MEGA7. Phylogenetic trees were bootstrapped with 1000 resampling iterations. iTOL and MEGA7 were both used for tree visualisation. (S1 Fig).
