# Supplementary material for: Assembling a plug-and-play production line for combinatorial biosynthesis of aromatic polyketides in Escherichia coli
Source: PLoS Biol. 2019 Jul 18;17(7):e3000347. doi: 10.1371/journal.pbio.3000347 (PMC6638757; doi:10.1371/journal.pbio.3000347)
Supplement: S2 Text — (DOCX) [file pbio.3000347.s029.docx]

**S2 Text. Elucidation of the major metabolites produced by the anthraquinone biosynthetic gene cluster**

The first steps of the anthraquinones biosynthetic pathway are predicted to be congruent to the biosynthesis of actinorhodin, the archetypal octaketide from *Streptomyces coelicolor*[[1](#_ENREF_3)]. Identification of expected octaketide shunt metabolites after each biosynthetic step enables interrogation of the AntA-I biosynthetic logic, either confirming or refuting the biosynthetic congruence shown in Fig 2. Additionally, identification of shunt metabolites at each biosynthetic step provides a snapshot of any metabolic bottlenecks within the biosynthetic pathway which can guide pathway engineering to optimise end compound biosynthesis. Several octaketide shunt metabolites are isomeric and therefore discrimination by parent mass alone is not reliable; here retention time, MS^2^ and HPLC-UV-Vis-MS aided characterisation when compared with existing literature[[2](#_ENREF_4),3].Masses corresponding to each modified octaketide shunt metabolite **(2-11)** were identified exclusively in the AntA-I expressing host, however at extremely low ion intensities preventing reliable identified using a targeted LC/MS approach; only protonated or deprotonated adducts were observed for each mass and isotopic masses were not visible (Fig 5, S10 Fig). The reduced ion intensity of shunt metabolites may indicate either the flux through the anthraquinone biosynthetic pathway is sufficient to avoid accumulation of intermediates or accumulated metabolites are being further derivatized to unknown end products which are not identified by our targeted MS analysis. To improve confidence when assigning MS peaks to expected shunt metabolites the corresponding extracted ion chromatogram (EIC) of AntA-I expressing hosts was compared to counterparts with individual ketoreductase, cyclase and cyclase/aromatase deletions (S11-12 Fig); here, the absence of sequential biosynthetic enzymes results in accumulation of shunt metabolites after each biosynthetic step. The high accumulation of shunt metabolites in hosts deficient in one biosynthetic enzyme enabled characterization of intermediates at each step by HPLC-UV-Vis-MS (S11-12 Fig). Intermediates were then used for comparative analysis of shunt metabolites from the AntA–I producing host.

## SEK4 and SEK4b

Should a bottleneck arise at the C9 ketoreduction, directly after the biosynthesis of the nascent polyketide chain, accumulation of two cyclised octaketides, SEK4 **(2)** and SEK4b **(3)**, would be expected (Fig 2). In the *antA–I* expressing *E. coli* BL21(DE3) cultures the unreduced isomeric octaketides SEK4 and SEK4b were observed at t_R_ 527 sec and t_R_ 569.4 sec by positive ionization and t_R_ 516, 527, 569.4 and 619.8 sec by negative ionization (t_R_ = 527 sec, ES^+^ [M+H]^+^ 319.08084, theoretical [M+H]^+^ 319.08123 and ES^-^ [M-H]^–^ 317.06610, theoretical [M-H]^–^ 317.0667, tR = 569.4 sec ES^+^ [M+H]^+^ 319.08005, ES^-^ [M-H]^–^ 317.06589, t_R_ = 516 sec ES^-^ [M-H]^–^ 317.06604 and t_R_ = 619.8 sec, ES^-^ [M-H]^–^ 317.06579) (S10 Fig). Theoretical exact, protonated and deprotonated masses for each shunt metabolite can be found in S3 Table.

Extracted ion chromatograms from Δketoreductase (KR) hosts also show that masses corresponding to SEK4 and 4b elute at 516 and 527 Sec, in good agreement with the AntA-I expressing strain and elution patterns of SEK4 and SEK4b in the literature[[4](#_ENREF_6)] (S19 Fig). Protonated and deprotonated adducts of each parent mass were observed along with a comprehensive list of additional adducts in the ΔKR strain. Interestingly, the dehydrated octaketides AUR367 and B26 were not observed reliably: the mass corresponding to a dehydrated octaketide eluting at 515.4 / 516 sec is more likely to be SEK4 where the predominant ion observed is [M-H_2_O+H]^+^ and not [M+H]^+^, consistent with octaketide metabolite adduct profile from *E. coli* BL21 expressing *antDEFBG* (Fig 2). Additional adducts at this retention time correspond to the hydrated octaketide rather than adducts of AUR367 or B26. This is a previously described characteristic of SEK4[[5](#_ENREF_7)] and, together with the adducts described above, enables assignment of the first elution as SEK4, the second as SEK4b and the third as AUR367. Furthermore, UV-Vis spectra for SEK4 and SEK4b from ΔAntA show λ_max_ of 231 and 279 nm in agreement with previously published data[2,3] (S12 Fig). Chromatographic peaks corresponding to SEK4 and SEK4b were not detected in the *antA-I* expressing host, at 279 nm, consistent with the both metabolites being present at extremely low concentrations.

## Mutactin, SEK34 and their dehydrated counterparts

The next enzymatic step in the proposed biosynthesis of AQ256 is stereospecific reduction of C9 carbonyl group to a hydroxyl group catalyzed by a ketoreductase (Fig 2). Intramolecular aldol condensation of the ACP-linked polyketide chain results in formation of the first six-membered carbocyclic ring which is subsequently aromatised by a bifunctional aromatase/cyclase (ARO/CYC), or in the case of the anthraquinone cluster a tridomain aromatase/cyclase. Inactivation of the ARO/CYC during biosynthesis of actinorhodin or other aromatic octaketides results in the accumulation of mutactin **(6)** and its dehydrated form dehydromutactin **(7)**. The aromatic nascent chain undergoes a second intramolecular aldol condensation between C5 and C14 catalyzed by an additional cyclase forming a common bicyclic intermediate[[4](#_ENREF_8)]. Inactivation of this cyclase results in accumulation of SEK34 **(8)** and SEK34b **(19)**. Importantly, it is from the common bicyclic intermeditate that the anthraquinone biosynthetic pathway diverges from benzoisochromanequinones.

Identification of mutactin and SEK34, and their dehydrated counterparts, poses a challenge in the AntA-I expressing cultures using LCMS/MS due to their isomeric nature. Comparative analysis of the exometabolome from ΔAro/Cyc (AntH) and ΔCyc (AntC) enabled identification and disambiguation of trace amounts of mutactin, dehydromutactin, SEK34 and SEK34b, however. Accumulation of mutactin is expected in the ΔAntH strain and a mass pertaining to mutactin adducts are present at t_R_ 582.6 sec ([M–H]^–^ 301.0712, [M+H]^+^ 303.0861, theoretical masses [M–H]^–^ 301.0718, [M+H]^+^ 303.0863) (S11-12 Fig) which is in agreement with masses for the octaketide shunt product observed in the AntA-I expressing host (t_R_ = 582.6, ES^-^ [M–H]^–^ 301.0713, theoretical mass [M–H]^–^ 301.0717) (S20 Fig). Similarly, masses corresponding to SEK34 accumulated at t_R_ 574.2 sec in ΔAntC, ([M–H]^-^ 301.0711, [M+H]^+^ 303.0856) matching an additional retention time corresponding to the ambiguous modified octaketide in the AntA-I expressing host exometabolome (t_R_ = 574.2 [M–H]^–^ 301.0713, [M+H]^+^ 303.0858) (S17 Fig), enabling annotation of masses eluting at 582.6 sec as mutactin and those eluting at 574.2 sec as SEK34. Mutactin and SEK34 from ΔAntH and ΔAntC cultures were further characterized by HPLC-UV-Vis-MS where absorbance maxima and masses were in good agreement with previous publications[[2,3](#_ENREF_4)] (S17 Fig, S21 Fig) further validating the function of AntA and AntH.

By sequentially knocking out one gene at each biosynthetic step of anthraquinone biosynthesis, with the exception of AntI, we show the biosynthetic pathway to be congruent with actinorhodin. Metabolites were normalised to final cell optical density (OD_600_) and exometabolome was analysed in an unbiased manner using both positive and negative electrospray ionisation.

## Identification of trihydroxyanthrone (12) end product

The rationalised end compound of the anthraquinones cluster is proposed to be a trihydroxylated anthrone, as no monooxygenase is present within the anthraquinone BGC from *P. luminescens* TT01. Non-cluster associated monooxygenases are proposed to be involved in modification of this anthrone to the anthraquinones observed in the native producer[[1](#_ENREF_3)].

Masses corresponding to 1,3,8-trihydroxyanthrone **(12)** were observed in trace amounts exclusively in the supernatant of *E. coli* BL21(DE3) cultures expressing *antA-I* at one main retention time (t_R_ 537 sec) (S10 Fig). The observed mass at t_R_ 537 sec, [M+H]^+^ 243.06481, is within 1.6 ppm of theoretical mass [M+H]^+^ 243.06519 of **(12)**; however, only the protonated adduct was identified. Additional analysis using negative ionization corroborated the presence of C_14_H_10_O_4_ at t_R_ 537 sec, albeit at extremely low ion intensity rendering the annotation of 1,3,8-trihydroxyanthrone tentative (S10 Fig). As mentioned in the main text anthrones have been shown to form cognate anthraquinones and dianthrones via various oxidative mechanisms which may occur during cultivation or during sample preparation. Therefore the corresponding anthraquinones and dianthrones were targeted in subsequent analysis.

## Dianthrone elucidation

Masses corresponding to 1,3,8 trihydroxydianthrone **(13)** were present exclusively in *antA–I* expressing cultures at two retention times presumably corresponding to *trans* and *meso* forms (dianthrone 1: t_R_ 838.2 sec, [M+H]^+^ 483.1074, [M-H]^-^ 481.0918 and dianthrone 2: t_R_ 864.6 sec [M+H]^+^ 483.1071, [M-H]^-^ 481.0919, all masses within 2.5 ppm of the theoretical *m/z* [M+H]^+^ 483.1074 and [M-H]^-^ 481.0928) (S10 Fig). Furthermore, MS^2^ spectra of both dianthrone 1 and 2 indicate the putatively assigned 1,3,8 trihydroxydianthrones to fragment to the respective anthrone radical C_14_H_10_O_4_^●–^ (S12 Fig): fragmentation of the C10 – 10’ bond is a hallmark of MS^2^ spectra from a wide variety of glycosylated and aglycone dianthrones[[7](#_ENREF_9)]. UV-Vis absorbance of putative diathrones 1 and 2 showed similarities to emodin dianthrone[8] and the dianthrone sennosides[9] with λ_max_  at 359, 263, 217 nm and λ_max_  358, 275, respectively (S13 Fig) collectively indicating that both masses correspond to authentic dianthrones. Full characterization by NMR was not possible as neither compound was present in sufficient quantities.

## Modified anthraquinone identification

AQ256 appeared to be the main anthraquinone detected from *E. coli* BL21(DE3) expressing *antA-I* however masses corresponding to the methylated AQ270a and b identified in *P. luminescens* were also detected with significantly reduced ion intensities (S10 Fig, S22 Fig). Whilst addition or substitution of auxochromic substituents is well documented to cause small batho- or hypsochromic shifts in λ_max_ of anthraquinones, no other intense peaks were identified at 434 nm suggesting the masses in question do not correspond to modified AQs. Should the concentration of methylated anthraquinones drop below the detection limit, and the masses observed are authentic AQs, the low level of methylation is more likely performed by an enzyme endogenous to *E. coli* which can accept AQ256 as a poor substrate than a cluster-associated enzyme, as methoxy-substituted AQs are not expected intermediates in AQ256 biosynthesis.

The more extensively modified anthraquinones, AQ300 and AQ314 observed in *P. luminescens* TT01[[1](#_ENREF_3)], were not detected here. A mass corresponding to the actinorhodin shunt metabolites aloesaponarin II **(11)** was observed but was also present with diminished ion intensity in the exometabolome of *E. coli* BL21(DE3) and *E. coli* BL21(DE3) pACYCDuet-1 at the same retention time and was therefore discarded. Interestingly, masses corresponding to DMAC **(10)**, the carboxylated precursor of aloesaponarin II, were observed at two retention times (t_R_ = 702 sec, ES^-^ [M-H]^-^ 297.0397 and t_R_ = 720 sec, ES^-^ [M-H]^-^ 297.0398) (S10 Fig), and fragmentation of the mass eluting at 702 sec is consistent with decarboxylation of the protonated parent ion to that of aloesaponarin II (data not shown). Similarly, MS^2^ of the mass at the second retention time shows decarboxylation of the parent mass at decreased ion intensity as well as a loss of water (data not shown). Identification of DMAC but not aloesaponarin II has been previously reported during the biosynthesis of bhimamycins form *Streptomyces* sp. AK671[[1](#_ENREF_12)0].

**References**:

1. Brachmann AO, Joyce SA, Jenke-Kodama H, Schwar G, Clarke DJ, et al. (2007) A type II polyketide synthase is responsible for anthraquinone biosynthesis in Photorhabdus luminescens. Chembiochem 8: 1721-1728.

2. Zhang WJ, Li YR, Tang Y (2008) Engineered biosynthesis of bacterial aromatic polyketides in Escherichia coli. Proceedings of the National Academy of Sciences of the United States of America 105: 20683-20688.

3. Ma SM, Zhan JX, Xie XK, Watanabe KJ, Tang Y, et al. (2008) Redirecting the cyclization steps of fungal polyketide synthase. Journal of the American Chemical Society 130: 38-+.

4. Nicholson TP, Winfield C, Westcott J, Crosby J, Simpson TJ, et al. (2003) First in vitro directed biosynthesis of new compounds by a minimal type II polyketide synthase: evidence for the mechanism of chain length determination. Chemical Communications: 686-687.

5. Hong H, Spiteller D, Spencer JB (2008) Incorporation of fluoroacetate into an aromatic polyketide and its influence on the mode of cyclization. Angewandte Chemie-International Edition 47: 6028-6032.

6. Taguchi T, Awakawa T, Nishihara Y, Kawamura M, Ohnishi Y, et al. (2017) Bifunctionality of ActIV as a Cyclase-Thioesterase Revealed by in Vitro Reconstitution of Actinorhodin Biosynthesis in Streptomyces coelicolor A3(2). Chembiochem 18: 316-323.

7. Xu W, Zhang J, Huang ZH, Qiu XH (2012) Identification of new dianthrone glycosides from Polygonum multiflorum Thunb. using high-performance liquid chromatography coupled with LTQ-Orbitrap mass spectrometry detection: a strategy for the rapid detection of new low abundant metabolites from traditional Chinese medicines. Analytical Methods 4: 1806-1812.

8. Falk H, Schoppel G (1992) ON THE SYNTHESIS OF HYPERICIN BY OXIDATIVE TRIMETHYL-EMODIN ANTHRONE AND EMODIN ANTHRONE DIMERIZATION - ISOHYPERICIN. Monatshefte Fur Chemie 123: 931-938.

9. Park SB, Kim YS (2015) Simultaneous separation of three isomeric sennosides from senna leaf (Cassia acutifolia) using counter-current chromatography. Journal of Separation Science 38: 3502-3507.

10. Jetter P, Steinert C, Knauer M, Zhang GL, Bruhn T, et al. (2013) New bhimamycins from Streptomyces sp AK 671. Journal of Antibiotics 66: 719-726.
