# Supplementary material for: Assembling a plug-and-play production line for combinatorial biosynthesis of aromatic polyketides in Escherichia coli
Source: PLoS Biol. 2019 Jul 18;17(7):e3000347. doi: 10.1371/journal.pbio.3000347 (PMC6638757; doi:10.1371/journal.pbio.3000347)
Supplement: S3 Text — (DOCX) [file pbio.3000347.s030.docx]

**S3 Text. Elucidation of Neomedicamycin (1,3-dihydroxy-8-methoxyanthraquinone) and neochaetomycin (1,3,8-trihydroxy-monochloroanthraquinone)**

Through ‘bolting-on’ an additional secondary tailoring enzyme to the AntA-I BGC in *E. coli* BL21(DE3) AQ256 was derivatised, forming two new chemical entities, neomedicamycin and neochaetomycin.

Supplementation of the AntA-I pathway with the gene for IFMT (also known as IOMT 3, SAM dependent isoflavone 7-O-methyltransferase, GenBank: AAY18582.1) from the legume *Medicago truncatula* resulted in biosynthesis of a methylated AQ256 derivative as identified firstly by LC-ESI-MS (Fig 7): observed mass of [M-H^-^]^-^: 296.04568, 0.5 ppm from theoretical mass of a methylated anthraquinone [M-H^-^]^-^ 269.04555 (S18 Fig). Anthraquinones were purified from *E. coli* BL21(DE3) expressing *antA-I* and *ifmt* and analysed by HPLC as described in S2 Text. Eluents were monitored at 434 nm; two peaks were detected opposed to the single peak observed for *E. coli* BL21(DE3) expressing the anthraquinone BGC alone. Both peaks were collected, the first of which (tR: 15.6 min) corresponds to neomedicamycin and the latter peak (tR: 28.9 min) corresponded to AQ256. neomedicamycin showed λmax at 214, 245, 282 and 429 nm and λmin at 236, 256 and 316 nm (S18 Fig), a similar absorbance spectrum to AQ256 (S15 Fig). Additionally, neomedicamycin was characterised by NMR (^1^H, COSY and HSQC – S19 Fig). The ^1^H NMR spectra is similar to that for AQ256 (S14 Fig); however, a single peak can be observed at ~4 ppm corresponding to the methoxy group hydrogens. Using NMR alone, assignment of the C8 methoxy-group was difficult; the crystal structure for neomedicamycin was solved to disambiguate this conclusively (S19 Fig).

Calculation of the amount of neomedicamycin present in the NMR sample were as follows:

- - The neomedicamycin is dissolved in 600 μL of methanol-d_4_.
  - Also present in the methanol-d_4_ is tetramethylsilane (TMS) as a standard at 0.03% vol/vol. This corresponds to 0.18 μL of TMS in the NMR sample.
  - Integration of the NMR spectrum, with the resonance from the methoxy proton of neomedicamycin (observed at 4 ppm) set to a value of 3.00, gave an integration of 12.94 for the 12 equivalent methyl protons from TMS (observed at 0 ppm).

The molar ratio of tetramethylsilane (TMS) to neomedicamycin in the NMR sample can be calculated using the following relationship:

$$\frac{\mathbf{12.94 integral for TMS}}{\mathbf{12.00 protons}}\boldsymbol{:}\frac{\mathbf{3.00 integral for methoxy}}{\mathbf{3.00 protons}}$$

This gives a 1.08:1 molar ratio of TMS to neomedicamycin.

The number of mols of TMS (*n*(TMS)) present can be calculated using the density of TMS (0.6488 g/mL) and the molecular weight (88.23 g/mol):

$$\boldsymbol{n(}\mathbf{TMS}\boldsymbol{)}\mathbf{=}\frac{\boldsymbol{0.18 \mu L\cdot0.6488}\frac{\mathbf{mg}}{\boldsymbol{\mu}\mathbf{L}}}{\mathbf{88.23}\frac{\mathbf{mg}}{\mathbf{mmol}}}\boldsymbol{=0.001324}\mathbf{mmol}\boldsymbol{=1.324}\boldsymbol{\mu mol}$$

Therefore the number of mols of neomedicamycin present in the NMR sample is:

$$\boldsymbol{n(}\mathbf{neomedicamycin}\boldsymbol{)}\mathbf{=}\frac{\boldsymbol{1.324 \mu mol of TMS}}{\mathbf{1.08}}\boldsymbol{=1.228}\boldsymbol{\mu}\mathbf{mol}$$

The molecular weight of neomedicamycin is 270.24 g/mol, so the mass (*M*) of neomedicamycin in the NMR sample is:

$$\boldsymbol{M}\mathbf{=270.24}\frac{\boldsymbol{\mu}\mathbf{g}}{\boldsymbol{\mu}\mathbf{mol}}\boldsymbol{\cdot1.228}\boldsymbol{\mu}\mathbf{mol=331.806}\boldsymbol{\mu}\mathbf{g=0.33 mg}$$

Therefore the final concentration in 600 μL is 0.55 g/L.

Supplementation of the AntA-I plug-and-play scaffold with RadH, from *Chaetomium chiversii* produced the monochlorinated anthraquinone 1,3,8-trihydroxy-monochloroanthraquinone, or neochaetomycin. As with IFMT, hydroxyl- anthraquinones are not previously characterised substrates for RadH, despite this masses corresponding to neochaetomycin were detected exclusively in *E. coli* BL21(DE3) expressing *antA-I* and *radH* indicating RadH to accept anthraquinone substrates. Culture supernatant from *E. coli* BL21(DE3) expressing *antA-I* and co-expressing the *antA-I* pathway and *radH* were analysed by LC-ESI-MS as described in S2 Text, however used an extended linear gradient from 5% to 95% B over 15 min. Neochaetomycin was detectable only in culture extracts expressing *radH* with an observable mass of [M-H]^-^ 288.99066, 0.9 ppm for the theoretical mass of [M-H]^-^ 288.99092 (S19 Fig) and isotope pattern consistent with chlorination: the deprotonated ^37^Cl neochaetomycin adduct ([M-H]^-^ 290.9875) was observable at a 1:3 ratio to ^35^Cl neochaetomycin (data not shown). Anthraquinones were purified from *E. coli* BL21(DE3) expressing *antA-I* and *radH* and analysed by HPLC as described in S2 Text. Once more, eluents were monitored at 434 nm, where two peaks (tR: 25.15 min and 33.8 min, respectively) were detected opposed to the single peak observed for *E. coli* BL21(DE3) expressing *antA-I*. Both metabolites were collected, the first to elute corresponded to AQ256 and the latter to the monochlorinated AQ256 derivative, neochaetomycin. The UV-visible spectrum of neochaetomycin was similar to that of AQ256: λ_max_: 213, 243, 276 and 432 nm, λ_min_: 238, 253, 335 and 353 nm (S19 Fig).

## NMR spectroscopic and X-ray crystallographic analysis of neomedicamycin and neochaetomycin.

Neomedicamycin containing fractions were combined and evaporated to dryness before dissolution in 600 μL deuterated methanol (0.03% tetramethylsilane (TMS)) for characterisation by NMR spectroscopy. A 400 MHz Bruker NMR spectrometer was used to record ^1^H and COSY NMR spectra, whilst a 500 MHz Bruker NMR spectrometer was used to obtain HSQC and HMBC NMR spectra (S19 Fig). Assignment of the peaks was performed by analysing chemical shift, coupling constant, COSY, HSQC and HMBC data.

^1^H NMR (500 MHz, methanol-*d*_4_), δ ppm: 4.02 (s, 3 H, CH_3_), 6.53 (d, *J* = 2.3 Hz, 1 H, H-2), 7.13 (d, *J* = 2.4 Hz, 1 H, H-4), 7.54 (d, *J* = 8.5 Hz, 1 H, H-7), 7.78 (t, *J* = 8.0 Hz, 1 H, H-6), 7.89 (d, *J* = 7.6 Hz, 1 H, H-5).

## CCDC 1813180 contains the supplementary crystallographic data for neomedicamycin. These data can be obtained free of charge via www.ccdc.cam.ac.uk/conts/retrieving.html (or from the Cambridge Crystallographic Data Centre, 12 Union Road, Cambridge CB21EZ, UK; fax: (+44)1223-336-033; or deposit@ccdc.cam.ac.uk).

Neochaetomycin containing fractions were evaporated to dryness before dissolution in 600 µL of deuterated methanol (0.03% TMS) for characterisation by NMR spectroscopy. Low yields of this compound prevented full characterisation by NMR spectroscopy and only a ^1^H NMR spectrum was obtained (S19 Fig)**,** which showed that chlorination occurred at either the C2 or the C4 positions.

^1^H NMR (600 MHz, methanol-*d*_4_), δ ppm: 7.10 (s, 1 H, H-2 or H-4), 7.23 (dd, *J* = 7.5, 1.0 Hz, 1 H, H-7), 7.61 (t, *J* = 7.8 Hz with additional splitting unresolved, 1 H, H-6), 7.70 (dd, *J* = 7.6, 0.9 Hz, 1 H, H-5).
